# The key to improving the beauty of the giant retaining wall in valleys: Increasing visual extension

**DOI:** 10.1371/journal.pone.0287251

**Published:** 2023-06-29

**Authors:** Jialu Song, Yanzuo Zhou, Weiyang Xiao, Qin Zeng, Yixuan Wu, Huixing Song

**Affiliations:** 1 Department of Environmental Design, College of Landscape Architecture, Sichuan Agricultural University, Chengdu, China; 2 Jiuzhaigou National Nature Reserve Administration, Aba Tibetan and Qiang Autonomous Prefecture, Aba, China; University of Veterinary and Animal Sciences, PAKISTAN

## Abstract

The retaining wall is a passive engineering measure to prevent and control unsafe factors caused by rock collapse in the valleys. Existing studies have mainly focused on its functional robustness and safety features, with few exploring its visual quality in the landscape. A multiple regression analysis was applied to evaluate the Scenic Beauty Estimation (SBE) of the giant retaining wall in Jiuzhaigou’s (a world natural heritage site) Heye Village, then the factors affecting SBE were analyzed. It is found that enhancing the sense of perspective and spatial hierarchy of retaining-wall murals in narrow roads contributes to the extension of observers’ sight, which is the key to improving SBE. Furthermore, the showcase of folk culture in murals can realize the beautification function of the giant retaining walls. In addition, the SBE of giant retaining walls is also linked to coordination, where the walls embellished with the natural landscape and folk culture murals have better SBE performance than those with local stones. This study provides a reference for constructing scenic beauty after fulfilling the safety function of retaining wall engineering.

## 1. Introduction

Located in the southeastern margin of the Qinghai-Tibet Plateau, the western part of Sichuan province is a typical mountainous area covered by plateau mountains and hills [1]. In the process of tectonic evolution, this area has experienced many tectonic movements and is still one of the most seismically active areas in China [2]. Consequently, frequent earthquakes expose residents living in the mountains and valleys to multiple geological hazards and potential dangers such as mountain collapse, landslides, and mudslides [3].

The giant retaining wall [4] is a crucial engineering approach for warding off falling rocks and rolling stones on mountain slopes in western Sichuan. For the residents in the mountains and valleys, the giant retaining wall is a passive protection measure of pile-slab concrete structure, which serves as a safety barrier, and its stability and safety determine its irreplaceability in function. A large number of studies have examined the safety design [4], reliability design [5], and structural design [6] of retaining wall engineering technology. Nevertheless, retaining walls are often gigantic, solid, and bulky, causing severe visual pollution to local landscapes. Furthermore, in the valley, the retaining wall is commonly adjacent to roads, forming a narrow passage space, affecting the visual quality of the environment for pedestrians [7]. Besides, the visual perception of similar structures, such as highway bridges, noise barriers, and other engineering structures, is also not well received [8, 9].

Administrators, engineers, and designers have attempted to dissipate the negative visual impact of these structures on the environment. Although some studies point out that retaining walls can achieve positive visual effects in urban streets and improve the scenic appearance [10], its effect is unclear in the mountains and valleys. Currently, there are two main types of retaining wall beautification: one is to apply flat murals to improve the single and rigid color composition and forms [11], and the other is to employ three-dimensional materials like stones, timbers, and ceramics to decorate the retaining walls [12]. Although a unique visual effect can be presented through the combination of materials, the latter is seldom adopted in practice because of financial costs and safety concerns. In the mountainous areas of western Sichuan, giant retaining walls are plentiful for securing villages and roads against natural disasters. However, there is a lack of research on the SBE of giant retaining walls in geological engineering, environmental science, or landscape gardening. Therefore, it is vital to identify the aesthetic preferences of the public to enhance the SBE of giant retaining walls after fulfilling their safety mission [13].

Existing landscape assessment methods include “Landtype Hypothesis” [14], “Landcover Model” [15], “Psychophysical Model” [16], “Ecological Models” [17], and “Prospect-Refuge Theory [18]”. Scenic Beauty Estimation(SBE), proposed by the American environmental psychologists Daniel and Boster in 1976, is the most commonly used method [19]. This approach is based on psychophysics and is a way of evaluating landscape samples by the public, and has certain practical value [20]. Psychophysical evaluation of landscape aims at study the consistency and difference of landscape in people’s eyes and to analyze the main factors affecting landscape evaluation in an in-depth manner [21]. The psychophysics school advocates that landscape aesthetics is the result of the interaction between the evaluator’s perception and cognition. It is assumed that observers have a preferred perception of the landscape. Therefore, the observers’ visual perception is rated through a survey and then quantified as their cognitive evaluation of the environment.

The landscape visual quality of retaining walls is assessed through the sensitivity, universality, and applicability of SBE [22]. This is based on extensive empirical studies on SBE’s application in landscape architectures (such as forest parks [23], wetland parks [20], highway landscapes [24], natural heritage sites [25], and waterfront plant landscapes [26]). With the further development of SBE in recent years, it has also been applied to the evaluation of other types of objects, such as tea gardens [27], stone landscapes [28], and outer ring roads [13], as well as watershed management projects [7]. However, the assessment by SBE for giant retaining walls in the valley has not been done before.

Therefore, this paper adopts the SBE method to examine the giant retaining walls in Jiuzhaigou’s Heye Village, Sichuan Province. Based on the evaluation indexes of regional characteristics, the giant retaining walls with natural scenery murals, folk culture murals, and local stones are evaluated. This paper attempts to answer the following questions: 1) What are the main factors affecting the aesthetic value of giant retaining walls in valleys? 2) What effects do different beautification approaches have on the aesthetic value of giant retaining walls?

## 2. Materials and method

### 2.1 Overview of the study area

**Fig 1:**
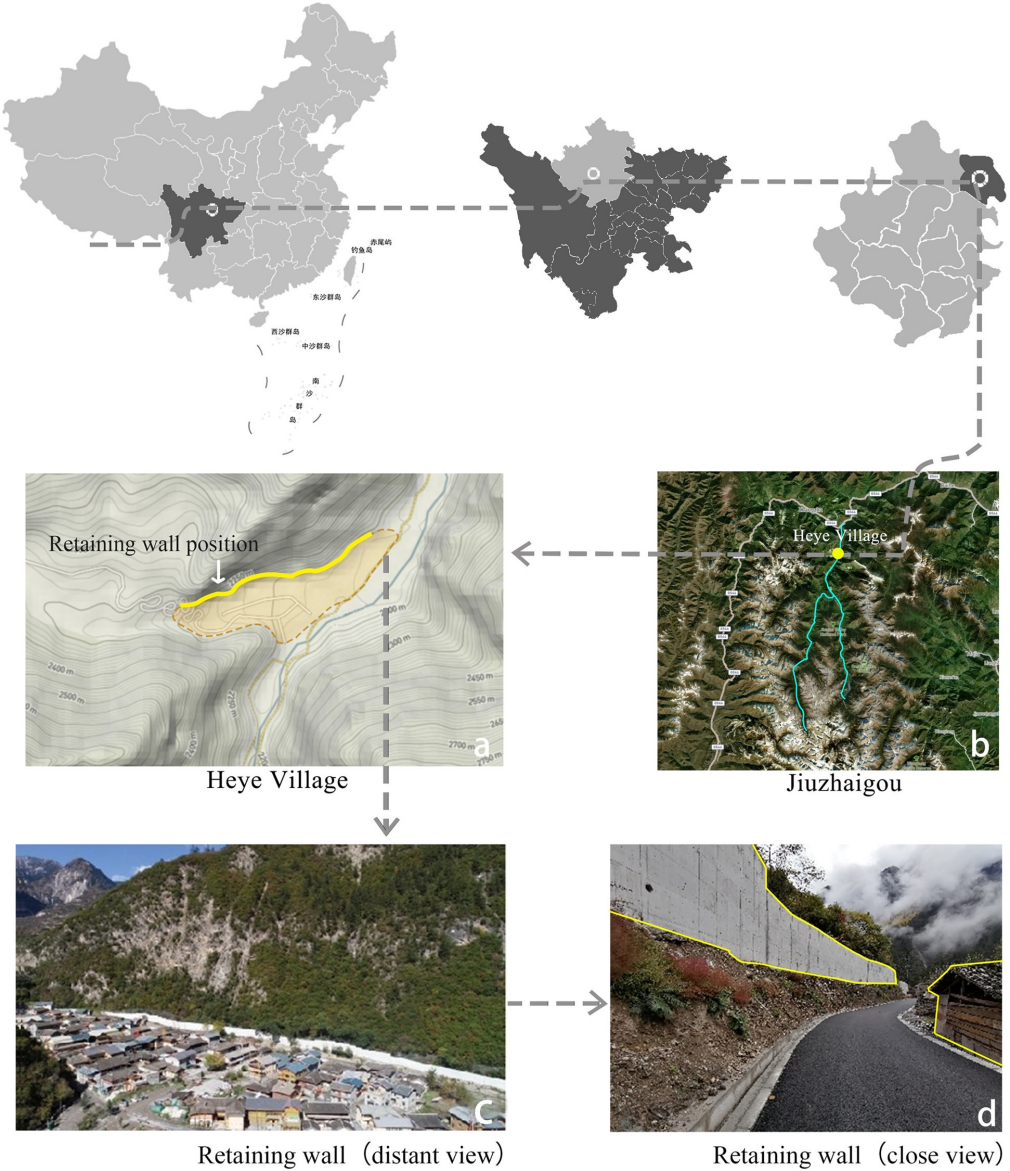
Location of Heye Village and photos of retaining wall. (a, b, Reprinted from map data Open Street Map contributors, map layer by Mapbox, under a CC BY 4.0 license). https://doi.org/10.6084/m9.figshare.21715967.v1.

The research object of this study is the giant retaining wall of Heye Village, a typical mountain village in Jiuzhaigou Scenic Area, Aba Prefecture, Sichuan Province, China. (33°N, 103°E) (Fig 1A). In 1992, Jiuzhaigou Scenic Area was included in the World Natural Heritage List for its “Outstanding Universal Value”. It is also the first nature reserve in China with the primary objective of protecting nature scenery. The reserve is a valley spanning over 50 kilometers (Fig 1B). Glacier erosion resulted in high slope fractures, forming a U-shaped valley with steep slope angles on both sides (Fig 1C) [29]. The local villagers’ houses were built on the foot of the mountains (Fig 1C). Heye Village is the largest village in this area. Due to the special geographical conditions, this region has been in a state of self-isolation for a long time. Thus, it has retained the original Amdo Tibetan folk culture, containing rich natural and cultural resources.

A 7.0-magnitude earthquake struck Jiuzhaigou on August 8, 2017. The earthquake not only caused severe infrastructure damage and casualties but also triggered a large number of landslides [30]. Furthermore, due to the loosening of the rock mass, landslides would occur anytime after the earthquake, which would bring severe safety hazards to villagers and tourists. In light of this, the local government built a retaining wall (Fig 1C and 1D) along the edge of Heye village. The gigantic wall is 558 meters long, 5–7 meters high, and 1.2 meters thick. The giant retaining wall ensures the safety of the village residents, but it is so large that it blocks the sight of pedestrians, and the landscape beauty is adversely affected (Fig 1D).

In reality, there are several ways to decorate the giant retaining wall, such as planting greeneries, painting murals, and embedding materials. Constrained by budget, Heye village settled on murals and local stones. Jiuzhaigou’s unique landscape and regional culture (local traditions, customs, festivals, and beliefs) were painted on the wall. Local stones were embedded in certain parts of the wall. The purpose was to reduce the adverse effects of the giant retaining wall by shifting visual attention.

### 2.2 Experimental design

The index construction of the SBE for retaining walls is multi-dimensional. For such unique engineering structures as giant retaining walls, no clear evaluation metrics have been proposed in the existing evaluation methods. Therefore, new evaluation indexes must be developed based on available visualization resources. Furthermore, the giant retaining walls in valleys are ubiquitous in the mountainous areas of western Sichuan and have a unique regional cultural background. Accordingly, this study innovates the evaluation indexes with “regionalization” under the mature SBE method system.

First, the retaining-wall SBE needs to follow the basic principles of landscape aesthetics, in which the evaluator’s visual perception is the most significant [31]. Second, there is previous evidence that perceptual processes are impacted by culture [32]. Therefore, the retaining-wall SBE should reflect not only the requirements of landscape aesthetics, but also the cultural characteristics nurtured in different regions. Third, this study deals with the fundamental concept of landscape ecology: landscape aesthetics, which is a part of the ecological service function [33] and emphasizes coordination. Hence, from the landscape ecology perspective, the giant retaining wall is seen as a whole, and the overall coordination of the environment should be fully considered. Many previous studies have proved that the overall coordination with the landscape environment is of great significance to the visual quality of the landscape [22, 34]. In short, it is necessary to establish an evaluation indicator system based on multi-dimensional features to evaluate landscape visual quality comprehensively and accurately.

Regarding the previous research results on influencing factors of SBE value [31-33], five perceptual indicators are identified (Table 1).

**Table 1:** Perception evaluation index of retaining walls and their description.

Evaluation index	Description of index
**Color richness(*X***_**1**_ **)**	The color richness of the murals on the giant retaining wall.
**Visual effect(*X***_**2**_ **)**	The perspective visual effect and visual extension of the retaining-wall murals.
**Cultural connotation(*X***_**3**_ **)**	The local folk culture of Jiuzhaigou showed by the murals on the giant retaining wall.
**Vegetation coverage(*X***_**4**_ **)**	Vegetation coverage of the giant retaining wall.
**Overall coordination(*X***_**5**_ **)**	the giant retaining wall adopts murals (natural scenery murals and folk culture murals) and natural materials (timbers and stones) to decorate the wall, which is in harmony with the natural ecological environment of the village.

Previous studies showed no significant difference between photos-based landscape quality evaluation and on-site evaluation [35]. However, when taking real on-site photos, it is necessary to avoid the influence of non-landscape factors and ensure the same shooting conditions (lens, aperture, and focal length). The sample photos were taken horizontally with a Nikon D5100 from the perspective of a pedestrian walking toward the giant retaining wall. The photos were taken from 9:00∼15:00 on July 28, 2021, in clear weather with high visibility. Twenty photos containing all the beautified scenes of the giant retaining wall in Heye Village were used as the evaluation samples.

Each evaluator rated the five perceptual indicators (independent variables) for each photo; then, the mean score of each indicator of each photo was taken as the quantitative value. The dependent variable is the SBE value calculated by the standardized formula after the evaluator scores the five perceptual indicators of the retaining-wall photos [19, 22]. To obtain valid independent variables, their relationship with the dependent variable must be significant, and collinearity between independent variables should be controlled. Therefore, the variance inflation factor (VIF) and tolerance value [34] are calculated for testing. A multivariate linear regression is adopted to evaluate the impact of each perceptual index on the SBE of the giant retaining wall in Heye Village.

### 2.3 Sampling and scoring

Previous studies also showed no significant differences in landscape evaluations between students and the general public [36, 37]. In terms of evaluators, experts and professional students outperform the general public in making aesthetic evaluations [38], and young students are less utilitarian in landscape appreciation [26], usually demonstrated as the pursuit of beauty and a kind of aesthetic value orientation [26]. Undergraduate students (both professionals and non-professionals) were also selected as evaluators due to various factors such as efficiency and convenience [39]. The evaluation of the image samples was conducted online. The questionnaire contains standardized instructions, a rating form, and relevant information on the gender, age, education, and professional background of the evaluators. A seven-point Likert scale was adopted [40], with the degree ranging from very poor (-3 points) to very good (3 points). Assigning positive and negative numbers on a 7-point scale (-3 to 3 points) is in line with people’s habits in that negative numbers are often used to indicate something unattractive, unappealing, or unfavorable, while positive numbers are used to represent something beautiful, positive, or favorable [41]. In addition, the evaluators were given background information to read before making judgments so that they were fully aware of the giant retaining wall construction and the local cultural knowledge of Jiuzhaigou to ensure a comprehensive understanding of the context. The evaluators were required to rate the five major indicators one by one with each sample photo. Eventually, there were 269 valid questionnaires obtained from 75 males and 194 females. Among them, 5.58% were less than 18 years old, 83.27% were 18–20 years old, 10.41% were 21–23 years old, and 0.74% were 24–26 years old. There were 159 students in the professional group, majoring in landscape architecture, environmental design, and landscape engineering. There were 110 students in the non-professional group, majoring in aquaculture science, land resource management, and agricultural and forestry economic management.

### 2.4 Calculation of SBE value

The evaluation value of each sample photo is standardized [42] to eliminate the random error brought by aesthetic differences among 268 evaluators. The calculation formula is

$$Z_{\text{ij}}=(R_{\text{ij}}-{\bar{R}}_{\text{j}})/S_{j}$$ (1)

$$SBE_{\text{i}}=\sum \text{j}Z_{i\text{j}}/N_{j}$$ (2)

Z_*ij*_ represents the standardized value of image *i* by evaluator *j*. R_*ij*_ is the evaluation value of evaluator *j* for image *i*, while ${\bar{R}}_{j}$ is the mean value of all images by evaluator *j*. S_*j*_ is the standard deviation of the evaluation value of all images by evaluator *j*(1).

SBE_*i*_ stands for the standardized value of image *i* by all evaluators. The average of all standardized scores for each image is the standardized value of SBE for each image. Z_*ij*_ is the standardized value of evaluator *j* for image *i*, and N_*j*_ is the number of total evaluators for image *i*(2).

### 2.5 Data processing

This paper assumes that there is a linear relationship between the five perceptual evaluation indicators of *X*_1_ (color richness), *X*_2_ (visual effect), *X*_3_ (cultural connotation), *X*_4_ (vegetation coverage), and *X*_5_ (overall coordination), and SBE value of the giant retaining wall in Heye Village. Multivariate linear regression analysis was adopted, and the retaining-wall SBE model was established. The validity of the model was verified through the multicollinearity test, degree of fitting (*R*^2^) test, *t*-test, and *F*-test. The Data processing adopted EXCEL and IBM SPSS 23.0 (SPSS, Inc., Chicago, Illinois, USA), including standardization of SBE values, analysis of variance, and regression analysis.

## 3. Research results

### 3.1 SBE value of retaining wall under different alleviation methods

**Fig 2:**
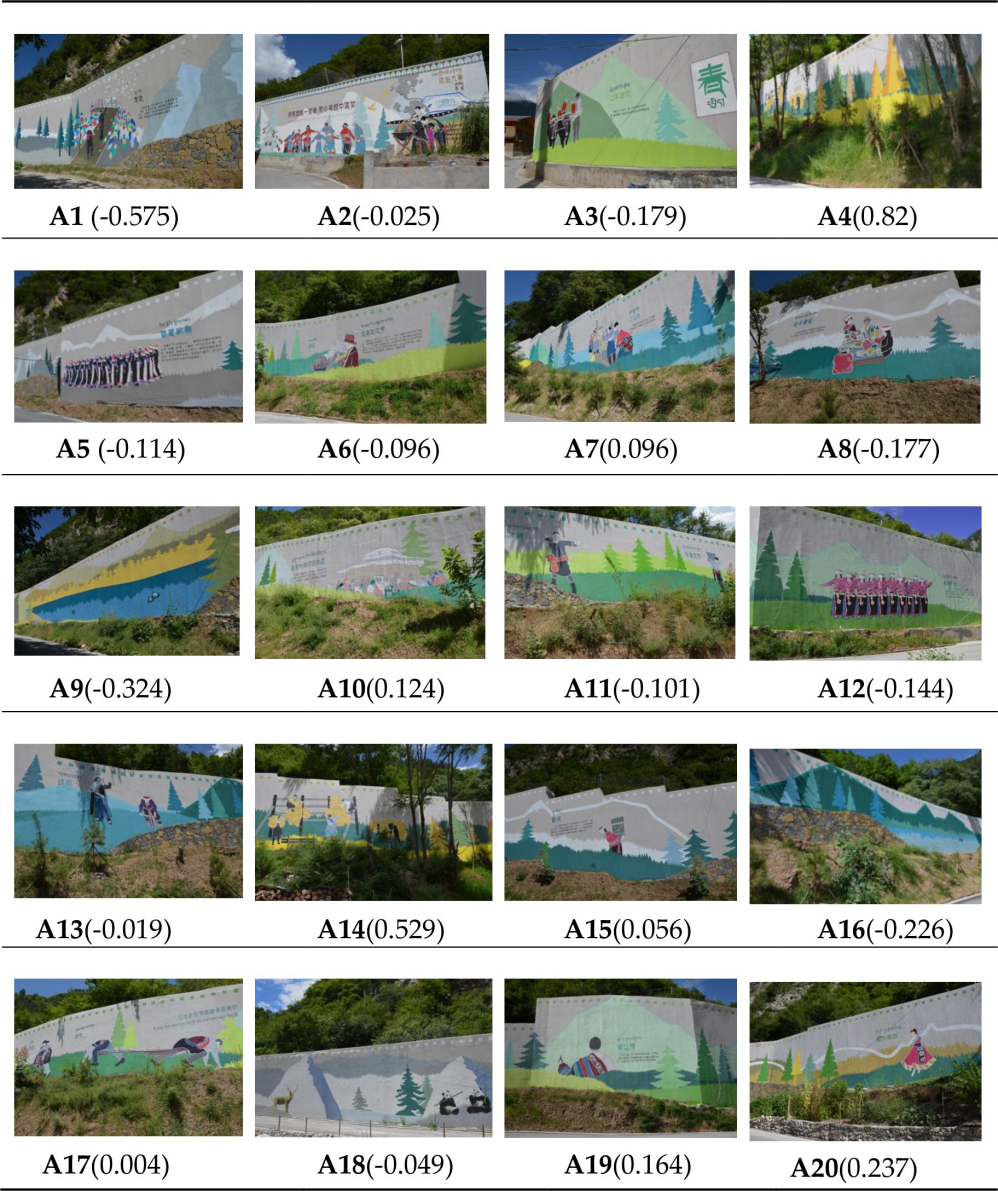
SBE value of sample photo. (Reprinted from *Graphic design collection of Jiuzhaigou folk culture with its post-disaster reconstruction projects* under a CC BY license, with permission from Jie Du, original copyright 2021). https://doi.org/10.6084/m9.figshare.21700970.v2.

The results indicated that the SBE values of the 20 sample photos of the giant retaining wall in Jiuzhaigou’s Heye Village range from 0.82 to -0.575 (Fig 2), of which 8 are positive (40%), and 12 are negative (60%). The SBE values are classified into three groups of low, medium, and high statistics: -0.65 ≤ SBE ≤ -0.15, -0.15 ≤ SBE ≤ 0.35, and 0.35 ≤ SBE ≤ 0.85. The low-value groups are A8 (-0.177), A3 (-0.179), A16 (-0.226), A9 (-0.324), and A1 (-0.575); the medium-value groups are A20 (0.237), A19 (0.164), A10 (0.124), A7 (0.096), A15 (0.056), A17 (0.004), A13 (-0.019), A2 (-0.025), A18 (-0.049), A6 (-0.096), A11 (-0.101), A5 (-0.114) and A12 (-0.144); the high-value groups are A4 (0.82) and A14 (0.529).

### 3.2 Impact of the background of evaluators on SBE value of retaining wall

The standardized value of each sample photo was analyzed by ANOVA to obtain the significance test value (*P* value) (Table 2). The results showed that, except for sample image A4 (*P*<0.05), the *P* values of the sample images were all greater than 0.05. This indicated that the professional background of elevators had no significant impact on the SBE of the giant retaining wall of Heye Village.

**Table 2:** ANOVA of standardized scores of sample images.

Image No.	*P* value	Image No.	*P* value	Image No.	*P* value	Image No.	*P* value
A1	0.477	A6	0.164	A11	0.192	A16	0.853
A2	0.562	A7	0.335	A12	0.082	A17	0.839
A3	0.5	A8	0.838	A13	0.362	A18	0.362
A4	0.02*	A9	0.837	A14	0.179	A19	0.943
A5	0.298	A10	0.371	A15	0.28	A20	0.691

(Note: * indicates *P* <0.05, meaning the difference is statistically significant)

**Fig 3:**
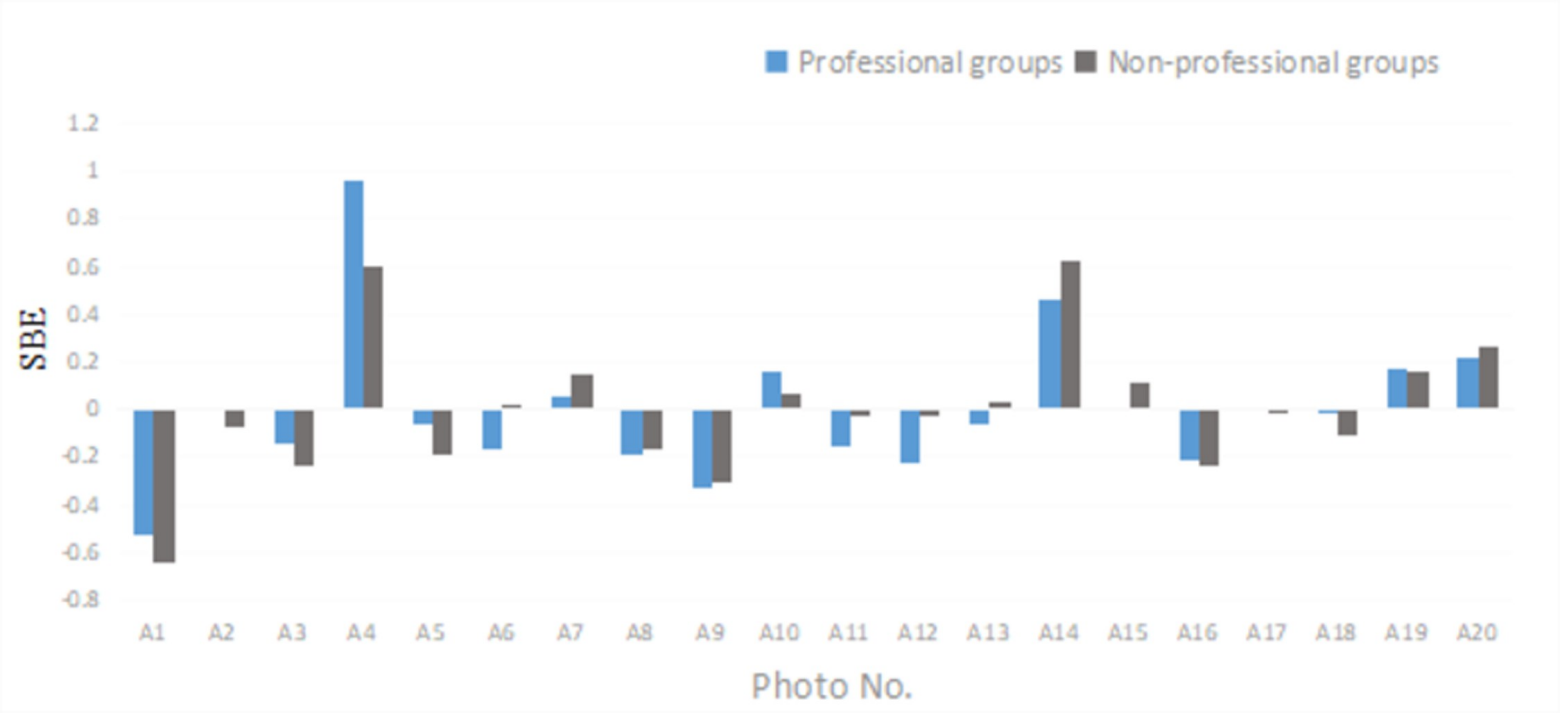
A comparison of SBE values in professional and non-professional groups.

By comparing the evaluation results of the giant retaining wall of the Heye Village between the professional and the non-professional groups (Fig 3), it was found that the fluctuation trends of the two lines were roughly the same, indicating no significant differences in the aesthetic preference between the professional and non-professional groups. Furthermore, the top three SBE values, A4 (0.82), A14 (0.529), and A20 (0.237), and the last three A1 (-0.575), and A9 (-0.324), A16 (-0.226) were compared between the professional and non-professional groups (the smaller the difference value, the more convergent the evaluation perception; otherwise, the more deviated). The results showed that the assimilation of the professional and non-professional groups in the evaluation of “beautiful” landscapes (the top three SBE values) was weaker than that of the “ugly” landscape (the bottom three SBE values).

### 3.3 Analysis of influencing factors of retaining wall SBE

The stepwise method in linear regression excluded the less important factors, *X*_1_ color richness and *X*_4_ vegetation coverage. The factors *X*_2_ visual effect (*sig* = 0.000<0.05), *X*_3_ cultural connotation (*sig* = 0.000<0.05), and *X*_5_ overall coordination (*sig* = 0.000<0.05), which contributed more to the SBE value, were retained. A regression equation for predicting the SBE of the giant retaining wall of Heye Village was formulated: *y* = -3.297+0.904*X*_2_+0.508*X*_3_+0.746*X*_5_. The absolute value of the standardized regression coefficient reflects the weight of the indicators’ contribution to SBE, where *X*_2_ is the most important, followed by *X*_5_ and then *X*_3_. The positive and negative signs reflect the direction of the contribution to SBE.

### 3.4 Model verification

First, the regression model was tested for multicollinearity through the variance inflation factor (VIF). According to the results, the VIF ranged within 1.064∼8.282 (VIF<10), and the tolerance was above 0.1, indicating no collinearity in the independent variables of the regression equation. The results of partial correlation coefficients were all significant by t-test (Table 3). The coefficient of determination *R*^2^ reflected the effect of linear regression. The closer the value of *R*^2^ to 1, the better the fitting degree [43]. The *R*^*2*^ of the regression model was 0.984 (Table 4), and the adjusted *R*^2^ was 0.981, indicating a high prediction accuracy of the regression model. The Durbin-Watson test [44] value was 1.582. The closer it was to 2, the worse the autocorrelation. Thus, the autocorrelation of the regression model was roughly good (Table 4). The validity of the regression model was tested by *F* (Table 5), and *F* was calculated to be 324.274(*P* = 0.000), indicating that the dependent variable *y* had a significant linear regression effect on the independent variables. Therefore, the three perceptual evaluation indicators selected were significantly correlated with the SBE value of the giant retaining wall. This model can be applied to predict the SBE value of the giant retaining wall of Heye Village.

**Table 3:** Regression analysis.

	Unstandardized coefficient		Standardized coefficient	t-test	Significance (*sig*)	Collinearity Statistics	
Mode	B	Standard error	Beta			Tolerance	VIF
**(Contant)**	-3.279	0.122		-26.814	0.000		
***X***_**2**_ **Visual effect**	0.904	0.144	0.576	6.293	0.000	0.121	8.277
***X***_**3**_ **Cultural connotation**	0.508	0.045	0.372	11.335	0.000	0.940	1.064
***X***_**5**_ **Overall coordination**	0.746	0.150	0.456	4.977	0.000	0.121	8.282

**Table 4:** Major parameters of the model (Reflecting superior accuracy of the model).

Model	*R*	*R* ^2^	Adjusted *R*^2^	Error of standard estimation	Durbin-Watson
3	0.992	0.984	0.981	0.41091	1.566

**Table 5:** ANOVA (The significance of the fitted equation reflects that the screened indicators have a remarkable effect on the SBE of the giant retaining walls in Heye Village).

Model	Sum of squares	Degree of freedom	Mean square	*F*	Significance
**Regression**	1.643	3	0.548	324.274	0.000
**Residual**	0.027	16	0.002		
**Total**	1.670	19			

## 4. Discussion

The SBE results of the giant retaining wall agreed with previous studies, indicating that groups with different backgrounds are roughly consistent in natural landscape aesthetics [45, 46]. The assimilation of evaluators in the evaluation of the “beautiful” landscape was weaker than that in the evaluation of the “ugly” landscape, which is inconsistent with the research results of Xuehong Tan [47]. This may be because the giant retaining wall’s unique topography and regional culture made the evaluators with different professional backgrounds easy to agree on the “ugly” ones. Educational background and cultural attainment will affect the evaluator’s cognition, leading to differences in the evaluation of scenic beauty. The beautification of the giant retaining wall in Heye Village is exploratory. It can be concluded that evaluators with professional backgrounds have greater ecological awareness and that non-professional evaluators have a more intuitive aesthetic reflection.

The purpose of psychophysical SBE is to study the consistency and difference of landscape in people’s eyes and to analyze the main factors affecting landscape evaluation [21]. In previous studies, many scholars have proved that color richness (*X*_1_) can significantly improve the visual environment quality of landscapes [21, 26]. In addition, vegetation coverage is significantly positively correlated with the visual environment quality of the landscape [48], and diverse vegetation types have a substantially positive impact on improving the visual environment quality of landscapes [49, 50]. However, the multiple regression results of this paper showed that the color richness (*X*_1_) and vegetation coverage (*X*_4_) of the giant retaining wall had no significant effect on the SBE value of the giant retaining wall, which is inconsistent with the findings of previous research. The possible reason is that designers are reserved about the color properties (hue, brightness, saturation) for murals on the giant retaining wall. The excessive color expression may strengthen the difference between murals and the surrounding environment and thus affect visual perception. The giant retaining wall intersections with the mountain in the valley, forming a steep and undulating narrow slope (average slope above 50°) at the roadside. In addition, the soil and water loss is severe, and the soil layer of the slope is thin, which makes it difficult to form a coordinated, green space. So the impact of natural vegetation coverage on the overall visual aesthetics is weak.

**Fig 4:**
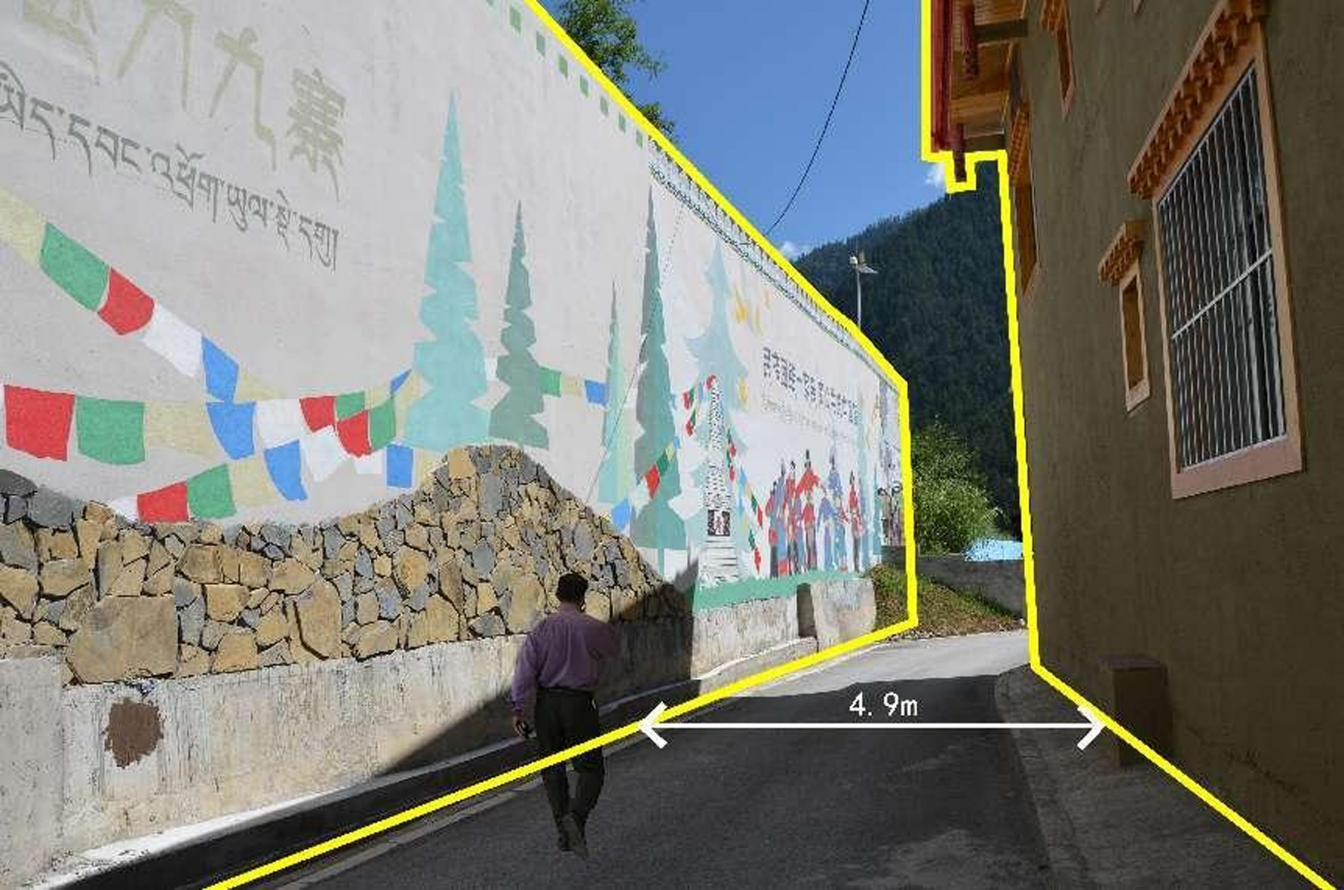
Mural scene of the giant retaining wall. (Reprinted from *Graphic design collection of Jiuzhaigou folk culture with its post-disaster reconstruction projects* under a CC BY license, with permission from Jie Du, original copyright 2021). https://doi.org/10.6084/m9.figshare.21716252.v1.

The SBE values of the giant retaining wall agreed with the results of multiple regression analysis. The visual effect (*X*_2_) of the retaining-wall mural is the most significant factor affecting the SBE of the giant retaining wall. The giant retaining wall at the edge of Heye Village forms a narrow space with the road, with the narrowest being less than 5 meters (Fig 4). The wall occupies most of the visible area, creating a sense of oppression for pedestrians. The high SBE value groups A4 (0.82) and A14 (0.529) depict Jiuzhaigou’s natural sceneries and cultural scenes. As a mural-filled space, the walls carry memories and create memories, enhancing the community’s charm [51]. Murals with natural sceneries and cultural scenes make the giant retaining walls more ornamental, which agrees with previous studies [20]. In addition, the overlapping method, oblique perspective, and color perspective were used in the formation of the murals, which created a sense of perspective and spatial hierarchy of “front-people, middle-water, back-mountain,” attracting visitors’ attention and reducing the stiffness and bleakness of the wall. The sense of perspective and spatial hierarchy embodied in the vertical beautification of the mural extends pedestrians’ sight on the narrow roads, enabling the mural to be visually extended in the spatial construction [11, 52]. Scenes in the medium-value group also employ natural scenery murals or folk culture murals with perspective effects. The murals would attract the pedestrians’ attention to offset the negative feeling brought by the giant wall. At the same time, the application of murals makes visual communication clearer and reality more vivid [53]. The murals utilize composition and stylistic design to infuse natural scenes with specific meaning and give full play to the benefits of visual extension, playing an essential role in beautifying the giant retaining wall.

According to the results, 9 out of the top 10 sample photos with the highest SBE values exhibited folk culture scenes. The results of multiple regression analysis also showed that the evaluators’ awareness of Jiuzhaigou folk culture (*X*_3_) also significantly impacted the SBE value of the retaining wall. Previous research has shown that the mural consists not only of an artistic aspect but also depicts the culture and way of life at the time it was created [54]. For instance, the mural of image A14 (0.529) depicts a harvesting activity in Jiuzhaigou in autumn; A20 (0.237) depicts the scene of a Tibetan girl wearing Hada dancing gracefully (a dance unique to Tibetan areas); A19 (0.164) depicts the scene of “Inviting the Mountain God” a unique folk culture activity in Jiuzhaigou. The rich cultural connotation of murals also diverts the negative perception of the giant walls from pedestrians and allows people to immerse themselves in the spirit conveyed by cultural symbols. Research shows that the role of murals in constructing cultural contexts is to strengthen the regional style and enhance the psychological implication [11]. The mural shows the folk culture based on its aesthetic features and realizes its environmental function by unveiling the folk culture content. It creates a unique spatial perception experience, arousing the viewer’s spiritual sense of belonging and cultural identity. This visual communication emphasizes various characteristics of the cultural process [55], including historical, social, material, ideological, and regional features.

As an engineering protection measure made of reinforced concrete, the giant retaining wall is poorly coordinated with the surrounding environment, reflecting the inhomogeneity and complexity of the spatial distribution of landscape elements and their properties, that is, the heterogeneity of landscapes. The murals with different contents (natural scenery murals, folk culture murals) and local stones alleviate the heterogeneity between the giant retaining wall and the surrounding environment, promote the visual coordination of the landscape (*X*_5_), and contribute to SBE improvement. Nevertheless, A16 (-0.575), A9 (-0.324), and A1 (-0.226) gain the lowest SBE values, and these samples all use the local stones to embellish some parts of the murals. In other words, murals with local stones are less effective than natural scenery murals and folk culture murals in beautifying the giant retaining wall. The reason may be that the local stones only cover a smaller area compared to the mural area, which is less likely to attract the viewer in their visual perception. Furthermore, if large numbers of local stones are used to upgrade the texture of the vertical wall, it will increase the load of the giant retaining wall and thus affect its safety and stability. Another point of concern is that, from the perspective of engineering cost, the giant retaining walls in mountain streams and valleys are often built at the steep slopes at the foot of the mountain. Therefore, the transportation and construction of local stones invariably consume plenty of resources and funds. Nevertheless, the enhancement of the SBE is less effective. This reminds the administrators and designers to make wise selections of materials for retaining wall beautification.

While our research has addressed some regional issues, some problems remain to be solved: What are the differences in the perceived beautification of engineering structures in different countries and cultures? How to incorporate beauty evaluation into the management and comprehensive evaluation system of large engineering structures? Future studies may explore the weights of evaluation indicators for engineering structures, such as safety, beautification, and economic and cultural value, from an interdisciplinary perspective.

## 5. Conclusions

This study explores the factors affecting the scenic beauty of giant retaining walls in the valleys of western Sichuan. It is found that visual effect, cultural connotation, and coordination are all positively related to the SBE of giant retaining walls. Focusing on the sense of perspective and spatial hierarchy of retaining-wall murals in narrow roads contributes to the extension of observers’sight, which is the key to improving SBE. Moreover, the display of folk culture elements in murals can realize the beautification function of the giant retaining walls. In addition, the SBE of giant retaining walls is also linked to coordination, where the walls embellished with the natural landscape and folk culture murals have better SBE performance than those with local stones.

It is recommended that designers may include murals with local cultural elements when they design giant retaining walls in valleys. If the road is narrow along the giant retaining wall, it is essential to enhance the sense of perspective and spatial hierarchy of the murals to extend the viewers’ sight. In addition, special attention should be paid to the overall coordination of the giant retaining wall with the surrounding environment.

The findings of this study may be applied to the construction, management, and development assessment of geological and landscape engineering, which is vital to human well-being.

## Supporting information

S1 File Original data of Scenic Beauty Estimation (SBE) scores of 20 samples. (PDF)
