# Supplementary material for: The key to improving the beauty of the giant retaining wall in valleys: Increasing visual extension
Source: PLoS One. 2023 Jun 29;18(6):e0287251. doi: 10.1371/journal.pone.0287251 (PMC10309985; doi:10.1371/journal.pone.0287251)
Supplement: S1 File — (PDF) [file pone.0287251.s001.pdf]

Sequence number	1.Gender	2.Degree education	3.Professional background	1-1.Color richness	1-2.Visual effect
1	2	1	1	5	5
2	2	1	1	7	7
3	2	1	1	6	6
4	1	1	1	6	5
5	1	1	1	7	6
6	1	1	1	7	7
7	2	1	1	6	6
8	2	1	1	4	2
9	1	1	1	2	3
10	1	1	1	6	6
11	2	1	1	6	6
12	2	1	1	4	4
13	1	1	1	5	4
14	1	1	1	6	6
15	2	1	1	4	5
16	2	1	1	5	5
17	2	1	1	6	5
18	2	1	1	6	5
19	2	1	1	6	6
20	2	1	1	5	5
21	2	1	1	6	5
22	2	1	1	5	3
23	2	1	1	5	5
24	2	1	1	2	2
25	2	1	2	6	6
26	1	1	1	6	6
27	1	1	1	5	5
28	2	1	1	6	5
29	2	1	1	6	3
30	2	1	1	5	3
31	1	1	1	5	6
32	2	1	1	4	3
33	2	1	1	7	6
34	1	1	1	6	5
35	1	1	1	7	7
36	2	1	1	5	5
37	1	1	1	3	3
38	2	1	1	5	5
39	2	1	1	2	2
40	2	1	1	6	5
41	2	1	1	6	5
42	2	1	1	5	5
43	2	1	1	6	6
44	2	1	1	6	7
45	2	1	1	5	4
46	2	1	1	6	4
47	2	1	1	6	6
48	2	1	1	5	6
49	2	1	1	5	2
50	2	1	1	6	5
51	2	1	1	3	3
52	2	1	1	6	5
53	2	1	1	6	5

54	2	1	1	5	4
55	2	1	1	6	5
56	2	1	1	6	5
57	2	1	1	6	6
58	2	1	1	5	4
59	1	1	1	6	3
60	2	1	1	6	2
61	2	1	1	6	5
62	2	1	1	4	4
63	2	1	1	6	6
64	2	1	1	5	5
65	2	1	1	6	5
66	2	1	1	5	5
67	2	1	1	7	7
68	2	1	1	6	5
69	2	1	1	4	4
70	2	1	1	6	5
71	1	1	1	4	4
72	1	1	1	6	5
73	2	1	1	5	4
74	2	1	1	5	5
75	2	1	1	6	6
76	2	1	1	5	5
77	2	1	1	5	6
78	2	1	1	5	5
79	2	1	1	5	4
80	2	1	1	6	4
81	1	1	2	6	6
82	1	1	2	4	5
83	1	1	2	7	7
84	1	1	2	7	6
85	2	1	2	5	5
86	1	1	2	5	5
87	1	1	2	5	3
88	1	1	2	5	5
89	2	1	2	6	6
90	1	1	2	7	7
91	2	1	2	7	7
92	1	1	2	6	5
93	2	1	2	7	7
94	2	1	2	6	7
95	2	1	2	7	7
96	2	1	2	5	5
97	1	1	2	6	6
98	1	1	2	7	7
99	1	1	2	3	4
100	2	1	2	6	6
101	2	1	2	5	5
102	2	1	2	7	7
103	2	1	2	5	5
104	1	1	2	6	5
105	1	1	2	6	6
106	2	1	2	5	5
107	2	1	2	6	6
108	2	1	2	6	6
109	1	1	2	3	6

110	2	1	2	5	6
111	2	1	2	6	6
112	2	1	2	6	6
113	2	1	2	5	5
114	2	1	2	5	6
115	2	1	2	4	4
116	2	1	2	7	7
117	2	1	2	6	5
118	2	1	2	4	3
119	1	1	2	6	6
120	2	1	2	5	5
121	2	1	2	5	6
122	2	1	2	6	4
123	1	1	2	5	5
124	1	1	2	5	5
125	1	1	2	5	5
126	2	1	2	6	5
127	2	1	1	4	5
128	2	1	2	6	5
129	1	1	2	6	6
130	1	1	2	6	6
131	2	1	2	5	5
132	2	1	2	6	6
133	2	1	2	5	4
134	2	1	2	5	5
135	2	1	2	6	5
136	1	1	2	7	6
137	1	1	2	5	4
138	2	1	2	6	7
139	2	1	2	6	6
140	2	1	2	5	5
141	2	1	2	7	6
142	2	1	2	5	5
143	2	1	2	5	5
144	2	1	2	4	5
145	2	1	2	6	5
146	2	1	2	7	7
147	1	1	2	7	7
148	2	1	2	4	4
149	1	1	2	5	5
150	1	1	2	5	5
151	2	1	2	6	5
152	2	1	2	6	6
153	1	2	2	5	4
154	2	1	2	6	6
155	2	1	2	5	4
156	2	1	2	6	6
157	2	1	2	6	6
158	2	1	1	6	5
159	2	1	1	1	3
160	2	1	1	6	6
161	2	1	1	6	6
162	2	1	1	6	6
163	1	1	1	6	6
164	2	1	1	7	7
165	2	1	1	6	6

166	2	1	1	6	6
167	1	1	1	6	6
168	2	1	1	5	5
169	2	1	1	5	5
170	2	1	1	5	5
171	1	1	1	5	5
172	1	1	1	6	6
173	2	1	1	6	5
174	2	1	1	6	5
175	2	1	1	6	5
176	2	1	1	6	5
177	1	1	1	6	6
178	2	1	1	5	5
179	1	1	1	5	3
180	2	1	1	6	6
181	2	1	1	6	5
182	2	1	1	4	5
183	1	1	1	3	2
184	2	1	1	7	7
185	2	1	1	6	6
186	2	1	1	5	5
187	2	1	1	5	5
188	2	1	1	6	5
189	2	1	1	4	4
190	2	1	1	5	5
191	2	1	1	5	5
192	2	1	1	7	2
193	2	1	1	5	6
194	2	1	1	4	3
195	2	1	1	6	6
196	2	1	1	5	6
197	2	1	1	5	3
198	2	1	1	4	3
199	1	1	1	6	5
200	1	1	1	6	7
201	2	1	1	3	4
202	2	1	1	3	3
203	2	1	1	6	4
204	2	1	1	6	6
205	1	1	1	5	5
206	1	1	2	5	5
207	2	1	1	3	4
208	1	1	1	6	6
209	1	1	1	7	7
210	2	1	1	6	5
211	2	1	1	6	6
212	2	1	1	5	4
213	1	1	1	3	2
214	1	1	1	6	7
215	2	1	1	5	5
216	2	1	1	5	5
217	2	1	1	5	4
218	1	1	1	7	7
219	2	1	1	4	5
220	2	1	1	5	5
221	2	1	1	2	3

222	1	1	1	4	4
223	2	1	1	5	5
224	2	1	1	3	3
225	2	1	1	5	6
226	2	1	1	7	7
227	2	1	1	2	2
228	2	1	1	5	4
229	2	1	1	6	5
230	1	1	1	5	5
231	1	1	1	5	6
232	2	1	1	2	2
233	1	1	1	5	4
234	2	1	1	5	5
235	2	1	1	2	2
236	2	1	2	5	6
237	2	1	1	3	3
238	2	1	1	3	3
239	1	1	2	6	6
240	1	2	2	6	7
241	1	1	2	5	4
242	2	1	2	5	6
243	2	1	2	5	5
244	2	1	2	6	5
245	1	1	2	5	5
246	1	1	2	1	1
247	2	1	2	5	5
248	2	1	2	6	5
249	1	1	2	6	6
250	2	1	2	5	5
251	1	1	2	4	3
252	2	1	2	6	5
253	2	1	2	6	6
254	1	1	2	5	1
255	2	1	2	5	5
256	2	1	2	6	6
257	2	1	2	4	4
258	1	1	2	7	7
259	1	1	2	5	6
260	2	1	2	5	5
261	2	1	2	6	6
262	1	1	2	4	5
263	2	1	2	3	3
264	2	1	2	7	7
265	2	2	2	6	6
266	2	1	2	5	6
267	2	1	2	5	6
268	1	2	2	6	6
269	2	1	2	5	5

1-3.Cultural connotation	1-4.Vegetation coverage	1-5.Overall coordination	2-1.Color richness	2-2.Visual effect	2-3.Cultural connotation
6	5	5	5	5	7
7	7	7	7	7	7
5	5	5	6	6	5
5	6	6	6	6	6
7	7	6	7	7	7
7	7	7	7	7	7
6	6	6	5	5	5
4	2	3	5	3	4
5	3	4	6	5	6
6	6	6	6	6	6
6	6	6	5	5	5
5	4	4	5	4	6
6	3	3	5	4	6
6	6	6	7	7	7
6	3	4	4	4	6
6	4	5	5	6	5
5	4	5	6	5	6
6	6	6	7	6	6
6	6	6	6	6	6
6	6	6	3	4	6
7	4	5	6	6	7
5	6	5	6	5	6
5	5	5	5	5	6
4	2	2	4	4	4
7	5	6	3	4	4
7	6	6	6	6	7
6	5	6	5	4	6
4	5	5	6	5	7
2	6	6	6	6	6
3	4	5	5	5	6
4	6	5	6	4	6
4	5	5	5	4	5
6	6	6	7	7	7
3	5	5	5	5	6
7	4	6	6	6	7
5	2	6	5	5	6
5	3	4	4	3	6
5	4	4	5	5	5
4	2	2	5	5	6
7	6	6	7	6	5
5	4	5	6	5	5
6	5	5	5	5	6
7	5	6	7	6	7
5	5	6	7	6	6
6	5	5	6	4	5
5	5	5	5	5	6
5	2	4	7	7	7
6	5	6	6	6	7
5	3	5	6	5	7
7	6	6	7	7	7
4	4	6	5	5	5
6	5	4	5	4	5
5	4	5	6	5	5

6	4	4	5	4	6
5	5	5	6	6	7
5	5	5	6	5	5
7	6	6	6	6	7
6	6	6	6	5	6
4	3	3	6	5	6
5	4	3	6	5	6
6	5	6	5	5	6
6	4	5	5	5	5
7	6	5	7	7	7
6	4	5	6	5	6
6	4	5	6	5	6
5	5	5	6	5	5
7	7	7	7	6	7
6	4	5	5	5	7
5	4	4	6	5	6
6	4	4	5	5	6
2	2	2	2	2	2
5	5	6	5	4	5
3	3	5	5	4	7
5	6	6	6	6	6
7	6	6	7	7	7
6	6	6	6	7	7
6	5	5	6	6	6
6	5	5	6	6	6
5	3	3	5	5	7
5	5	4	6	4	5
6	6	6	6	6	6
6	5	6	5	5	7
7	7	7	7	7	7
6	6	7	6	6	6
5	5	5	5	5	6
5	5	5	5	6	5
3	4	5	5	4	4
5	5	5	5	5	5
6	6	6	6	6	6
7	7	7	7	6	7
7	6	7	7	7	7
6	6	5	6	4	6
7	7	7	7	7	7
7	6	7	7	6	7
7	7	7	7	7	7
7	6	5	6	5	6
7	6	4	6	6	6
7	7	7	7	7	7
5	3	3	5	4	4
6	7	7	7	5	7
6	5	5	6	6	6
6	6	6	6	6	7
6	5	5	5	5	6
5	5	5	5	6	6
3	5	4	3	4	6
6	5	5	6	6	7
6	6	6	5	5	6
6	6	6	6	6	6
7	6	5	5	5	7

6	4	5	5	5	6
6	5	5	5	5	6
6	6	6	6	5	6
5	4	4	4	4	6
6	5	5	6	7	6
5	3	4	5	5	5
5	5	5	7	7	6
7	4	4	7	7	7
5	5	5	5	4	5
5	5	5	6	4	6
6	6	5	6	6	7
5	6	6	6	6	6
5	4	5	6	6	6
3	4	3	6	4	5
6	5	6	6	5	6
5	4	4	5	4	5
6	6	5	6	4	6
5	3	3	4	3	4
6	6	6	6	6	7
6	5	5	7	6	6
7	7	6	6	6	6
5	5	5	5	5	5
5	5	5	6	6	6
4	4	4	6	6	6
5	5	5	6	5	6
7	5	5	7	6	7
6	6	7	7	7	7
6	5	5	6	5	6
7	6	5	7	7	7
7	7	7	7	5	7
5	4	5	5	6	6
6	6	6	7	6	6
4	4	4	7	7	7
5	5	5	6	6	6
4	4	4	5	5	6
5	5	3	6	5	6
6	6	7	7	7	7
7	7	7	4	4	7
4	4	2	5	5	6
6	3	4	5	5	4
5	6	5	5	5	6
6	6	5	6	6	6
7	7	7	7	7	7
4	3	3	6	5	6
5	5	6	6	6	6
6	5	5	6	5	6
6	6	6	7	7	7
6	6	6	7	7	7
6	6	6	4	4	5
4	4	4	5	4	5
6	6	6	7	6	7
7	6	6	7	7	7
6	6	6	7	7	7
6	6	6	6	6	6
7	7	7	7	7	7
6	6	6	6	6	6

6	5	6	7	6	7
6	6	6	6	5	6
6	4	5	5	5	5
4	4	5	5	5	5
5	5	5	6	6	6
5	5	5	5	5	5
6	6	6	6	6	7
6	5	6	5	5	6
5	5	5	6	6	6
6	5	5	6	4	5
4	3	3	6	5	5
7	6	4	6	6	7
5	5	6	7	6	7
5	5	5	6	6	5
7	6	6	7	6	7
6	4	4	6	5	6
3	5	4	6	6	7
3	4	2	5	6	6
7	6	6	7	7	7
7	6	7	6	6	7
4	4	5	5	4	5
5	5	4	6	6	6
6	5	4	5	5	6
5	5	4	5	5	6
4	4	5	6	6	6
7	6	6	6	6	6
7	1	1	7	3	7
6	4	4	5	4	4
3	4	5	6	3	6
6	6	6	5	5	5
6	7	7	7	6	7
4	5	4	6	5	5
5	3	3	5	5	6
6	6	6	5	5	5
6	6	7	5	6	5
5	5	3	6	6	7
4	3	3	5	4	6
6	6	6	6	5	7
6	5	5	5	5	7
6	3	2	2	3	6
5	4	5	5	5	5
5	3	5	6	5	6
6	6	4	5	6	5
7	6	6	7	6	7
7	5	5	7	7	7
6	6	6	6	5	6
5	2	2	5	4	5
5	2	2	4	2	5
5	5	4	7	6	7
7	6	6	6	6	7
6	5	5	5	5	6
6	4	5	5	5	6
7	7	7	7	7	7
6	5	5	4	5	5
3	5	4	5	5	3
4	4	4	3	3	5

7	4	4	5	5	7
5	4	6	6	4	6
6	3	4	3	5	6
6	6	6	7	6	6
7	7	7	7	7	7
3	5	2	2	2	5
7	4	5	5	6	7
7	5	5	6	5	7
7	4	4	4	4	7
7	7	6	6	6	7
4	2	4	3	3	5
3	5	4	4	4	7
6	4	3	5	5	6
2	2	2	3	2	3
6	6	7	6	5	6
6	5	5	3	5	7
5	3	3	5	5	5
5	5	5	6	6	6
7	7	7	7	7	7
6	3	3	4	3	2
6	5	6	6	6	7
6	6	6	6	6	7
5	6	5	6	6	6
3	3	3	4	4	4
5	3	3	5	6	6
4	5	6	6	6	6
6	5	6	6	5	7
6	4	5	6	6	6
6	5	5	6	5	6
5	2	5	5	5	6
7	5	5	6	6	7
6	5	6	7	7	7
5	1	1	5	4	1
6	5	4	6	6	7
7	5	6	6	5	7
5	5	6	5	5	6
7	5	5	6	7	6
7	7	7	5	5	6
6	4	5	6	6	7
4	5	5	6	6	6
5	5	6	6	6	7
5	3	5	5	5	5
7	7	7	6	6	7
6	5	6	6	6	6
5	5	5	6	5	6
6	6	6	5	5	7
7	6	6	7	5	7
6	4	4	6	7	7

2-4.Vegetation coverage	2-5.Overall coordination	3-1.Color richness	3-2.Visual effect	3-3.Cultural connotation
5	5	5	5	7
7	6	7	7	7
5	5	6	6	5
6	6	5	4	6
7	7	7	7	7
7	7	7	7	7
5	5	5	5	5
4	3	4	3	4
5	5	3	4	4
6	6	6	6	6
5	5	5	5	5
4	4	4	5	5
3	3	5	4	6
6	6	6	6	6
4	3	5	4	6
6	7	5	6	5
6	5	5	5	6
6	6	5	5	6
6	6	6	6	6
5	5	5	6	7
5	5	6	7	7
5	5	5	3	5
5	4	5	4	5
4	4	3	3	4
4	4	4	4	5
6	6	5	6	6
5	4	4	5	5
5	5	5	5	5
5	4	6	6	5
3	5	4	3	4
5	4	5	5	6
4	5	4	5	5
7	7	6	5	5
3	5	6	4	6
6	7	1	1	1
3	5	5	5	5
5	3	3	4	6
5	5	4	5	5
4	5	3	2	4
5	6	5	4	5
4	4	5	4	5
5	6	5	6	6
5	6	6	7	7
6	6	6	7	7
6	5	4	4	5
5	5	5	5	6
6	6	5	3	5
6	6	6	6	6
4	6	6	5	6
6	6	5	4	6
5	5	5	5	5
4	4	5	5	6
4	5	6	6	5

5	4	3	4	5
5	5	5	5	5
5	5	6	5	6
5	6	6	6	6
5	6	5	6	6
5	6	4	3	5
2	5	6	6	6
5	6	5	5	6
5	5	4	5	5
6	6	5	6	6
5	6	6	6	6
5	5	6	6	6
5	5	5	5	5
6	6	6	5	6
6	6	6	5	6
4	3	4	5	6
5	5	7	6	6
2	2	3	3	3
5	5	5	4	5
5	4	4	5	7
6	6	6	7	6
6	6	6	6	7
6	7	6	6	7
6	6	6	6	6
5	6	5	6	6
4	6	6	6	7
4	4	6	5	5
6	6	5	5	5
6	5	6	6	6
7	7	7	7	7
6	6	6	6	6
6	6	6	6	6
5	5	5	5	5
4	4	5	4	4
5	5	5	6	5
6	6	6	6	7
6	7	7	6	7
6	7	7	7	6
6	5	5	4	5
7	7	7	7	7
6	6	7	6	7
7	7	7	7	7
6	6	7	6	6
6	6	5	6	6
7	6	6	6	7
4	4	3	4	5
6	6	7	7	7
6	7	6	6	6
6	5	6	6	6
5	5	5	6	5
5	5	5	6	6
2	2	3	4	5
5	5	7	5	6
5	5	6	6	6
6	6	5	6	7
5	5	6	5	7

3	4	5	4	6
5	5	5	6	6
5	6	6	6	6
4	4	4	4	5
6	5	6	5	6
4	5	4	4	4
5	6	7	7	7
5	5	7	7	7
5	4	5	4	5
4	6	6	5	6
4	4	5	5	5
5	5	5	6	6
5	5	5	5	5
3	3	4	5	6
5	5	6	5	6
4	4	4	6	6
6	3	5	3	6
2	2	4	3	4
5	5	6	6	6
6	6	3	2	5
6	6	6	6	6
5	5	5	5	5
6	6	6	6	6
4	5	6	6	6
5	5	4	4	6
6	6	5	6	7
6	7	6	6	7
5	5	4	5	6
7	6	6	6	7
6	6	4	5	7
5	5	5	6	6
6	6	7	6	7
6	5	6	6	6
5	5	5	5	6
5	5	5	5	6
4	4	3	3	6
7	7	6	6	7
4	4	7	4	7
4	3	4	4	4
4	4	5	5	3
6	5	5	5	5
6	6	6	6	6
7	7	7	6	7
4	5	5	4	5
6	6	6	5	5
5	5	4	5	5
7	7	6	6	6
6	6	7	7	6
4	4	4	4	5
3	5	3	2	2
6	6	6	6	6
6	6	7	7	7
6	6	7	6	6
6	6	5	5	6
7	7	7	7	7
6	5	6	6	6

5	6	5	5	6
6	6	6	6	6
5	5	6	6	6
5	5	5	6	6
6	6	5	4	5
5	5	5	5	5
6	6	6	6	7
4	5	6	6	6
5	6	6	6	6
5	4	6	5	6
4	4	5	5	4
4	4	6	5	6
7	6	5	6	6
4	5	5	6	7
6	7	5	5	6
5	4	5	5	6
5	6	5	5	6
4	5	5	6	6
5	6	7	7	7
7	6	6	6	7
4	4	5	5	5
4	3	5	5	6
5	4	5	4	4
4	5	6	5	5
4	5	5	6	5
6	6	7	6	6
2	2	2	2	7
4	3	6	6	5
2	4	4	3	5
5	5	6	6	6
7	7	5	6	6
4	5	6	6	7
4	4	4	4	5
5	5	5	5	6
7	6	6	7	5
6	6	5	5	5
5	5	4	4	6
4	4	4	4	6
5	5	5	5	5
4	4	5	3	6
5	5	5	5	5
4	5	6	7	7
6	5	6	6	5
6	6	7	7	7
6	5	6	5	6
5	5	5	5	6
3	3	5	4	5
2	2	3	2	5
6	7	7	6	6
6	6	7	7	7
5	6	7	7	6
5	4	5	5	6
7	7	7	7	7
5	5	5	5	5
5	3	5	5	5
2	2	3	3	3

5	5	4	4	7
4	5	6	6	6
3	3	6	6	6
5	6	6	6	6
5	7	7	7	7
6	2	2	2	6
4	5	6	4	7
4	4	5	5	7
6	4	4	5	7
7	6	7	6	7
4	4	2	2	5
2	4	6	7	6
3	3	6	5	6
1	2	3	3	3
5	5	5	5	6
5	6	6	5	4
3	3	3	3	5
6	6	6	6	6
7	7	6	6	6
2	2	7	5	7
6	6	7	6	6
5	6	6	6	7
6	6	6	6	6
5	5	4	4	3
6	6	7	7	5
6	6	6	5	6
6	5	5	6	6
5	5	6	6	6
6	6	6	6	6
6	5	6	6	5
6	6	6	5	7
6	6	6	6	7
1	1	6	6	6
6	6	5	5	6
5	5	7	7	7
5	6	5	6	6
5	6	7	6	5
7	7	6	6	7
5	6	7	5	6
6	5	6	6	6
5	4	4	4	5
5	5	5	3	5
5	4	7	6	7
5	6	6	6	6
6	5	6	5	5
6	3	6	6	6
7	7	6	5	7
6	6	7	5	7

3-4.Vegetation coverage	3-5.Overall coordination	4-1.Color richness	4-2.Visual effect	4-3.Cultural connotation
5	5	6	6	5
7	7	7	7	6
5	6	6	6	6
4	4	6	6	5
7	6	6	6	6
7	7	7	7	7
5	5	5	5	5
4	4	6	5	3
5	5	5	6	5
6	6	7	7	7
5	5	6	7	5
4	5	5	4	5
4	4	6	6	4
6	6	7	7	6
4	4	6	6	7
6	6	6	5	6
4	4	7	7	5
6	6	7	7	7
6	6	6	6	6
4	5	4	4	2
4	6	6	6	5
4	4	6	4	5
5	5	6	6	6
3	3	4	4	4
5	5	7	6	5
6	6	7	7	6
5	5	5	5	4
5	5	6	6	7
6	5	6	6	6
5	5	5	6	4
5	5	6	6	5
4	5	4	6	4
5	6	7	7	7
4	5	7	6	2
1	1	7	7	4
5	5	6	6	5
4	5	5	6	7
4	4	6	7	6
2	1	4	4	2
6	5	6	6	5
4	4	6	6	5
5	5	6	7	6
6	6	7	7	6
7	7	6	6	5
4	4	4	4	4
5	5	5	5	5
4	3	7	7	4
5	6	7	7	5
5	6	5	6	6
2	3	7	7	4
5	5	5	5	5
6	6	6	6	6
4	5	6	5	4

4	4	7	7	4
5	5	6	6	4
5	5	7	7	5
6	6	7	7	6
6	6	6	6	5
5	6	7	6	4
2	5	6	6	2
5	5	5	5	4
5	5	6	6	4
6	5	6	7	5
5	6	5	4	4
3	3	6	6	4
5	5	5	5	4
5	5	7	6	5
4	6	7	7	5
4	4	6	6	5
5	5	6	6	5
3	3	3	3	3
4	4	5	4	4
5	5	7	7	5
5	6	6	6	6
7	7	7	6	6
6	6	6	7	6
6	6	6	7	6
5	6	6	6	5
5	6	7	7	4
4	4	6	5	5
5	5	7	7	7
6	6	6	6	6
7	7	7	7	7
6	6	6	6	6
6	6	7	7	7
5	5	4	7	7
4	4	6	6	4
5	5	7	6	5
6	6	7	7	7
5	6	7	6	6
7	7	7	7	7
6	5	5	6	5
7	7	7	7	7
6	6	7	7	6
7	7	7	7	7
5	6	6	6	5
6	7	5	5	6
7	7	7	7	7
4	3	5	5	4
6	6	7	7	6
6	6	7	7	7
6	6	7	7	6
5	5	6	6	5
6	5	6	5	6
4	4	2	2	3
5	5	7	7	5
5	6	6	6	6
7	7	6	5	6
6	6	7	7	5

4	4	5	4	4
6	6	5	5	5
6	6	6	6	6
4	4	6	6	4
6	5	7	6	5
4	4	5	5	5
5	7	7	5	5
7	6	7	7	6
5	5	6	6	4
5	5	7	7	3
4	4	6	5	4
5	5	6	6	5
5	5	7	7	6
5	4	6	6	4
5	6	7	6	7
5	4	6	6	5
6	4	6	5	6
3	2	5	5	3
6	6	6	7	6
6	3	3	4	3
6	7	7	7	7
5	5	7	7	7
6	6	6	6	6
5	5	5	5	4
5	5	6	6	5
6	6	5	6	6
6	7	7	7	6
5	4	7	7	7
7	7	7	6	7
6	5	7	7	5
6	6	6	6	6
6	6	7	6	7
6	6	7	6	5
5	5	5	5	4
6	6	7	6	5
3	3	6	7	4
7	7	7	6	6
7	7	7	7	7
4	4	6	6	4
3	3	6	6	2
5	5	6	6	6
6	6	6	6	5
7	7	7	7	7
3	3	6	6	3
5	5	6	6	6
6	6	6	6	5
6	6	6	6	6
6	6	6	6	6
4	4	6	6	6
2	4	7	7	7
6	6	7	7	7
7	7	7	7	7
6	6	7	7	7
5	6	6	6	6
7	7	7	7	7
6	6	6	6	6

5	6	5	4	4
6	6	6	6	6
5	5	6	6	6
6	5	6	6	5
5	5	6	6	6
5	5	6	6	6
6	6	7	7	6
5	6	6	6	5
6	6	6	6	5
6	4	7	7	6
4	4	6	6	5
6	5	7	7	7
7	6	5	6	7
5	5	6	5	5
5	5	6	6	5
4	5	6	5	4
6	6	7	7	6
7	6	7	6	6
6	7	7	7	6
6	6	6	6	7
4	5	5	5	4
4	4	7	6	4
4	4	6	6	6
6	6	5	5	5
5	5	6	6	4
6	6	7	7	6
2	3	7	4	7
6	6	4	4	4
5	5	5	6	3
6	6	6	6	6
6	6	7	7	5
7	7	7	7	7
4	4	6	5	5
6	6	6	6	5
7	6	7	6	7
7	5	6	6	5
6	6	6	6	5
5	4	7	6	6
6	5	6	6	5
4	4	6	6	6
5	5	6	5	5
7	7	6	6	5
6	6	6	6	5
7	7	7	7	7
5	5	6	7	6
5	6	6	6	5
3	4	5	4	4
2	2	5	5	5
5	5	6	6	6
6	6	7	7	6
6	6	6	7	5
4	4	5	5	4
7	7	7	7	7
5	5	5	5	5
6	3	6	5	4
3	3	5	5	5

4	4	5	5	7
6	7	6	6	6
6	6	6	6	6
6	7	6	6	4
5	6	7	7	7
6	2	5	5	4
4	5	6	5	6
4	4	7	7	6
6	4	6	6	7
7	7	7	7	6
4	4	5	5	5
5	7	7	7	4
5	4	6	6	3
2	2	5	6	5
5	5	5	6	6
4	3	7	7	4
4	3	5	5	6
6	6	6	6	5
6	6	7	7	7
6	5	7	7	7
6	6	7	7	6
6	6	6	7	7
6	6	6	6	6
4	5	5	4	6
6	6	7	6	4
5	5	6	4	4
5	5	6	6	6
5	5	6	6	6
6	6	6	6	6
5	5	7	7	5
6	5	6	7	7
6	6	7	7	7
6	6	7	7	7
5	5	6	7	6
5	6	7	5	7
6	6	5	5	4
5	5	7	7	7
7	7	7	7	7
6	6	6	6	4
5	6	6	4	5
4	4	6	7	5
5	5	6	6	6
4	5	6	7	5
5	6	6	6	5
6	6	6	6	4
6	6	4	5	5
7	7	5	6	7
7	6	5	5	4

4-4.Vegetation coverage	4-5.Overall coordination	5.—1.Color richness	5-2.Visual effect	5-3.Cultural connotation
5	6	5	5	6
6	7	7	7	6
6	6	5	5	4
6	6	6	5	6
7	6	7	6	6
7	7	7	7	7
5	5	5	5	6
5	5	5	4	4
4	5	3	5	6
6	7	6	6	6
6	5	5	5	5
5	4	5	5	4
6	6	4	4	6
7	7	6	7	7
5	5	4	5	6
6	7	5	6	6
7	7	6	6	7
7	7	6	6	6
6	6	6	6	6
4	5	3	2	6
7	6	6	6	7
5	4	5	5	4
7	6	4	4	5
4	4	3	3	4
7	6	4	4	4
7	7	6	6	7
5	5	5	4	4
6	6	5	6	7
6	6	6	6	6
5	5	5	5	5
7	7	5	5	6
5	5	5	5	4
7	7	4	5	5
6	6	6	5	7
7	7	5	3	7
6	6	5	5	6
7	5	3	4	4
7	6	5	4	5
5	5	5	5	5
6	6	7	6	6
5	5	5	4	5
6	6	6	7	7
7	7	6	6	7
6	7	6	6	6
4	4	5	5	5
6	5	5	5	6
7	7	6	5	6
6	6	7	7	7
6	6	5	4	6
7	7	5	6	6
5	4	4	5	4
6	6	5	5	5
5	5	5	5	5

7	7	4	1	4
6	6	5	5	6
6	6	5	5	5
7	7	6	6	7
5	6	5	6	6
6	7	4	5	5
5	6	5	2	6
6	6	6	6	6
6	6	5	5	5
6	6	6	6	7
6	5	4	4	4
5	5	5	5	6
5	5	5	5	5
6	6	5	6	7
6	6	6	6	7
6	6	6	5	7
5	5	6	6	6
3	3	3	3	3
4	4	5	4	5
7	7	6	5	7
7	6	6	6	6
6	6	7	7	7
7	7	6	6	6
7	7	7	7	7
6	6	6	6	6
6	6	5	5	7
4	4	5	4	5
7	6	4	5	6
6	6	6	6	6
7	7	7	7	7
6	6	6	6	6
7	7	6	6	6
6	6	4	4	5
3	3	5	5	5
6	7	5	6	7
7	7	6	6	6
7	6	7	7	7
7	7	6	7	7
5	6	5	5	4
7	7	7	7	7
7	7	6	5	7
7	7	7	7	7
6	6	6	6	7
4	6	5	4	6
7	6	7	7	6
5	5	5	4	5
7	7	7	6	7
7	7	5	7	7
7	7	6	6	7
5	5	5	5	5
5	5	6	6	5
3	4	4	4	4
7	7	5	5	5
6	6	5	5	6
7	6	6	6	5
7	7	5	6	7

4	4	5	4	6
5	5	6	6	6
6	6	6	6	6
5	5	4	4	6
7	6	7	6	7
5	5	3	3	3
7	5	7	7	7
7	7	7	5	5
6	6	5	5	5
6	6	6	5	7
5	4	5	5	6
6	7	5	6	7
7	7	4	4	6
6	6	3	3	6
6	7	6	6	6
6	6	4	4	5
6	5	6	4	6
6	6	5	4	4
6	6	5	6	6
2	2	5	5	7
7	7	6	4	5
7	7	7	7	7
6	6	6	6	6
4	5	6	5	6
6	6	5	5	5
7	7	5	6	7
7	6	7	7	7
7	7	6	6	6
7	7	7	7	7
7	7	6	6	7
6	6	5	5	5
7	6	7	7	7
6	6	6	6	7
4	4	6	5	6
6	7	5	5	5
5	6	5	5	7
6	6	7	6	7
7	7	7	7	7
7	6	4	4	4
5	5	5	5	5
6	6	5	5	6
6	6	6	6	6
7	7	6	6	7
7	5	4	4	6
6	6	6	5	7
6	7	5	5	6
6	6	6	6	6
6	6	6	6	7
6	5	5	5	5
7	7	4	4	4
7	7	6	7	7
7	7	7	7	7
7	7	7	7	7
6	6	6	6	6
7	7	7	7	7
6	6	6	6	6

5	5	5	5	6
6	6	6	6	6
6	6	4	4	6
6	6	5	5	5
6	6	6	5	5
5	6	5	5	5
6	7	5	5	6
6	6	6	5	6
6	6	6	6	6
7	7	6	6	7
5	5	6	5	5
7	7	6	6	5
6	6	6	6	5
5	6	6	6	6
6	5	5	6	7
5	5	4	4	5
7	7	5	5	7
7	6	6	5	4
7	7	6	7	7
6	6	6	5	6
4	4	5	5	5
7	7	5	5	6
6	6	5	5	6
6	6	5	4	6
6	5	5	5	7
7	6	6	5	6
4	5	5	3	6
4	4	6	6	6
6	5	4	4	5
6	6	6	6	6
7	7	5	6	6
7	7	5	4	6
5	6	5	4	5
7	6	6	5	7
6	6	5	6	5
7	6	5	6	7
7	7	4	5	6
6	7	5	5	7
6	6	5	4	6
5	4	5	4	6
5	5	5	5	6
5	5	5	5	7
7	7	6	5	6
7	7	7	6	7
7	7	6	5	6
6	6	5	5	6
4	4	5	5	5
4	4	3	2	5
5	6	5	5	7
7	7	6	5	7
6	6	5	6	6
6	6	5	5	6
7	7	7	7	7
5	5	5	5	5
5	6	4	6	4
5	5	3	3	3

5	5	4	4	7
7	7	5	5	5
6	6	5	5	6
6	6	6	7	6
7	7	7	7	7
6	6	2	2	5
6	6	5	5	7
6	6	6	5	7
6	6	6	6	7
7	7	7	7	7
5	5	4	3	5
5	5	6	5	7
5	5	5	5	6
5	6	4	4	5
6	5	5	6	6
7	7	6	5	4
6	5	4	4	5
6	5	6	6	5
7	7	7	7	7
7	7	5	3	7
7	7	5	6	6
6	6	6	7	7
6	6	6	6	7
5	3	5	4	4
5	7	4	5	7
5	5	7	6	7
6	6	4	5	6
6	6	6	6	6
6	6	5	5	5
5	6	5	6	5
6	6	6	6	7
7	6	6	6	7
7	7	3	3	4
7	6	5	6	7
6	5	6	6	7
5	5	6	6	6
7	7	7	7	5
7	7	6	7	7
5	5	5	4	5
6	7	5	6	7
7	7	6	6	7
4	5	3	3	5
7	6	7	7	7
6	6	6	6	6
6	6	6	6	6
4	3	6	6	6
7	7	6	4	7
7	7	5	5	7

5-4.Vegetation coverage	5-5.Overall coordination	6-1.Color richness	6-2.Visual effect	6-3.Cultural connotation
5	5	5	5	6
7	7	7	7	6
4	5	5	5	5
6	6	6	6	6
7	7	7	7	6
7	7	7	7	7
6	6	6	6	6
4	5	5	4	4
4	4	3	4	5
5	5	5	5	6
5	5	4	4	4
5	5	6	6	4
3	3	5	4	6
6	5	6	6	6
5	5	4	5	4
5	6	5	5	5
5	5	7	6	7
6	7	6	7	7
6	6	5	6	5
3	3	6	5	5
5	5	5	5	5
6	6	5	5	6
4	4	5	5	5
3	3	2	2	2
4	4	6	5	5
6	6	6	6	6
4	5	4	4	4
6	5	5	6	4
6	6	6	6	6
4	4	6	5	6
4	4	5	4	5
4	5	4	5	4
3	3	4	4	4
3	4	6	6	6
4	4	4	4	7
6	6	5	5	6
5	3	4	4	6
5	5	5	5	5
3	5	5	5	5
7	6	5	4	6
5	5	5	4	5
7	7	5	5	5
5	6	6	6	6
6	6	6	6	6
4	4	4	4	5
5	5	5	5	6
4	4	5	5	6
5	6	7	6	6
5	5	6	6	6
4	5	6	5	5
5	5	4	5	4
5	5	5	5	5
4	5	5	5	5

2	4	3	3	3
5	5	6	6	6
5	5	5	5	5
6	6	6	6	7
5	6	6	5	6
5	3	4	3	4
2	4	6	5	7
5	5	5	5	6
5	5	5	5	5
6	6	6	6	5
5	5	6	5	6
3	3	5	5	5
5	5	5	5	5
6	6	6	6	6
5	5	6	5	5
5	5	5	5	6
5	5	4	4	5
3	3	3	3	3
4	4	5	5	5
6	4	6	7	7
6	6	5	5	5
6	6	6	7	6
6	6	6	6	6
7	7	6	7	7
6	6	6	6	6
5	6	5	5	5
4	4	5	4	4
6	4	5	6	6
6	6	6	6	6
7	7	7	7	7
6	6	6	6	6
6	6	6	6	6
5	5	6	3	6
5	5	5	4	5
5	6	5	5	5
6	6	6	6	6
7	7	7	7	7
7	7	7	7	7
4	4	5	4	4
7	7	7	7	7
6	6	5	5	7
7	7	7	7	7
6	5	5	6	5
6	5	6	6	6
6	7	7	7	6
5	5	4	4	5
6	6	7	6	7
6	5	7	7	6
6	6	5	5	7
5	5	5	5	5
6	6	6	5	5
4	4	4	4	4
5	5	5	5	5
5	5	6	6	6
6	7	5	5	6
5	5	7	7	7

4	4	5	5	5
5	5	5	6	6
6	6	6	6	6
4	4	5	4	5
6	6	5	5	5
3	3	4	4	5
5	7	7	5	7
4	5	7	7	7
4	4	4	4	5
1	1	6	5	7
4	4	5	4	5
6	7	6	5	6
6	6	5	5	6
3	2	5	6	6
5	6	6	5	6
4	4	5	4	5
5	5	6	4	6
3	3	5	5	5
5	6	6	6	6
5	5	6	5	5
5	5	7	7	7
7	7	7	7	7
6	6	6	6	6
5	5	6	7	6
5	5	6	5	6
6	6	6	5	6
6	6	7	6	7
6	7	5	6	5
7	7	7	7	7
7	6	6	5	6
6	4	5	6	5
6	6	7	7	7
5	5	7	6	5
5	5	6	6	6
5	5	5	5	5
3	3	5	5	5
6	7	7	6	6
7	7	4	4	7
4	4	4	4	4
4	3	6	6	6
6	5	5	5	5
5	6	6	6	6
7	7	6	7	7
4	4	5	4	5
6	6	6	6	6
5	5	6	5	6
6	6	6	6	6
6	6	7	6	6
5	5	4	4	4
4	4	6	6	6
7	7	7	7	7
7	7	7	7	7
7	7	6	6	6
6	6	6	5	6
7	7	7	7	7
6	6	6	5	6

6	5	6	6	6
6	6	6	6	5
4	5	5	5	5
5	5	5	5	5
4	4	5	4	4
5	5	6	6	6
5	5	7	7	7
5	6	5	5	6
6	6	6	6	6
6	6	7	7	7
4	5	5	4	4
3	4	5	5	4
6	5	5	5	5
6	6	6	6	6
6	5	5	5	6
5	4	5	5	5
6	6	6	6	5
6	4	5	5	3
5	5	6	6	6
6	6	6	6	7
4	5	5	5	5
4	5	5	5	5
5	5	6	5	6
6	5	6	5	5
5	5	4	4	4
4	6	6	6	6
3	2	5	2	7
6	6	4	4	4
3	3	5	3	5
6	6	6	6	6
6	5	5	5	5
5	5	5	5	5
4	4	5	4	5
6	6	5	6	7
7	6	5	6	5
7	6	4	5	6
4	3	5	5	6
4	4	5	4	6
5	6	5	5	6
4	4	6	6	6
5	6	5	5	5
4	5	6	5	6
6	5	6	5	5
6	6	7	7	7
6	7	6	7	6
5	5	5	5	6
5	5	3	3	3
3	3	3	3	5
3	5	4	5	5
6	6	6	6	6
6	6	5	4	5
4	4	4	5	5
7	7	7	7	7
5	5	5	5	5
6	4	4	6	5
3	3	3	3	3

4	4	5	5	7
5	5	5	4	6
4	4	6	6	6
6	6	6	6	7
7	7	7	7	7
6	3	2	2	5
3	5	6	5	6
4	4	6	6	7
6	6	7	7	7
7	7	7	7	7
4	4	3	3	5
6	6	6	7	6
3	3	6	6	6
5	4	4	4	3
6	6	5	5	6
3	4	4	5	6
4	4	4	4	5
6	6	5	6	6
7	7	6	6	6
3	3	7	7	7
6	6	6	6	6
6	6	7	6	7
6	6	7	7	7
4	4	4	4	6
5	5	7	6	6
6	6	7	6	5
5	6	6	6	6
6	6	7	7	6
5	5	6	6	5
6	5	6	5	6
6	5	6	6	7
6	6	7	7	7
3	4	5	5	5
6	6	5	6	6
5	5	6	6	7
4	6	5	5	5
7	7	7	7	6
7	7	6	7	7
4	4	5	6	6
4	5	6	6	6
6	6	5	5	6
5	5	5	5	5
3	5	6	5	7
6	6	6	6	5
6	6	6	5	6
6	6	6	6	6
7	7	5	6	6
6	6	7	6	7

6-4.Vegetation coverage	6-5.Overall coordination	7-1.Color richness	7-2.Visual effect	7-3.Cultural connotation
5	5	5	5	6
7	6	7	7	7
5	4	5	5	5
6	6	7	5	7
7	6	6	7	6
7	7	7	7	7
6	6	6	6	6
5	4	4	5	5
4	5	5	3	5
6	6	6	6	7
4	4	5	6	5
4	4	4	5	5
3	3	5	4	5
6	6	7	6	6
4	6	5	6	4
5	5	6	5	6
7	7	7	7	7
7	6	6	6	7
5	6	6	5	5
5	4	4	5	6
4	4	6	6	6
5	5	6	5	6
5	5	5	5	5
2	2	2	2	2
5	5	6	5	4
6	7	6	7	7
4	4	4	5	5
4	5	6	5	5
6	5	6	5	7
5	5	5	5	4
4	4	5	5	5
5	4	5	5	4
4	5	5	5	3
6	6	5	6	5
3	3	7	7	6
5	3	5	5	6
5	3	4	5	3
5	5	6	6	6
5	5	6	5	6
5	5	6	6	6
4	5	5	5	5
5	5	6	6	5
6	6	6	6	7
7	7	6	6	6
4	4	4	4	5
5	5	5	5	5
5	6	7	6	5
6	6	7	7	7
6	6	6	7	7
5	5	6	6	7
4	5	4	5	4
5	5	5	5	5
4	5	6	5	5

3	3	6	6	6
5	5	6	5	6
4	5	5	5	5
7	6	7	6	6
5	6	6	6	5
2	2	5	4	5
5	5	4	4	5
5	5	5	4	5
5	5	5	5	5
6	6	6	6	6
5	5	6	5	6
3	3	5	5	6
5	5	5	5	5
6	6	7	6	7
4	5	6	5	6
5	5	6	5	6
5	4	4	4	5
3	3	3	3	3
6	5	4	4	5
7	6	7	6	6
6	5	6	5	6
6	7	6	6	6
6	6	7	6	7
7	7	7	7	7
6	6	6	6	6
5	5	5	5	5
5	4	5	4	5
5	5	6	6	6
6	6	6	6	6
7	7	7	7	7
6	6	6	6	6
6	6	6	6	6
3	4	5	5	4
4	4	4	4	4
5	5	6	6	5
6	6	6	6	6
7	7	7	6	7
7	7	7	7	7
5	4	6	4	4
7	7	7	7	7
5	6	7	7	7
7	6	7	7	7
5	6	5	6	5
6	6	6	6	6
7	7	7	7	7
4	4	5	4	5
7	6	7	6	7
7	6	7	6	6
6	6	6	6	6
6	5	5	5	5
6	6	6	6	5
4	4	4	4	4
5	5	5	5	5
6	6	6	6	6
5	6	7	7	6
7	7	7	7	7

4	5	5	5	6
5	5	5	5	5
6	6	6	6	6
4	4	4	4	5
6	6	6	5	6
6	5	5	4	5
6	6	7	6	7
5	5	7	7	7
4	4	5	5	5
7	7	4	4	7
4	4	5	4	5
5	7	6	7	6
6	6	6	6	6
5	5	5	5	6
6	5	6	7	6
4	4	5	5	5
6	5	6	3	5
5	6	5	4	4
6	6	6	6	6
6	5	5	6	6
7	7	6	6	6
7	7	6	6	6
6	6	6	6	6
6	5	6	6	6
5	5	6	5	6
6	6	7	5	7
7	7	7	7	7
6	5	5	5	5
6	6	7	6	7
6	5	6	5	7
4	5	5	5	6
6	6	7	7	7
5	5	7	6	6
6	6	6	6	6
5	5	5	5	5
4	5	7	7	6
7	6	6	6	6
7	5	7	4	7
4	4	4	4	4
6	2	6	5	4
5	5	5	5	5
6	6	6	6	6
7	7	7	7	7
5	4	5	4	5
6	6	6	6	6
5	6	6	6	6
6	6	6	6	6
6	6	7	6	6
4	5	4	5	4
6	6	4	4	4
7	7	7	7	7
7	7	7	7	7
6	6	6	6	6
6	6	6	5	6
7	7	7	7	7
5	6	5	6	6

5	5	7	6	5
5	5	6	6	6
5	4	5	4	5
5	5	5	5	5
4	5	5	5	4
6	6	5	6	5
6	6	6	6	6
6	6	5	5	6
6	6	6	6	6
7	7	6	6	6
5	4	6	5	4
5	5	5	5	6
5	5	5	5	5
6	5	6	5	5
5	6	6	6	6
4	4	5	5	5
6	5	6	5	6
5	3	5	6	6
6	6	7	6	7
5	6	6	6	7
5	5	5	4	5
5	4	5	5	4
4	4	4	5	5
6	6	6	6	6
4	4	5	4	5
6	6	6	6	6
3	3	3	3	7
4	4	4	4	4
4	4	5	5	6
6	6	6	6	6
5	5	5	5	6
6	6	6	6	6
4	4	5	5	5
6	5	5	6	7
7	6	6	7	5
6	5	6	5	7
6	6	6	5	6
5	4	5	6	6
6	6	5	5	6
5	5	4	5	6
5	5	5	5	5
5	5	6	5	7
6	6	6	5	6
7	7	6	7	7
7	6	7	6	6
6	6	6	5	6
3	3	4	4	4
3	3	3	3	5
6	5	6	6	7
5	6	5	5	6
5	5	5	5	6
5	4	4	5	6
7	7	7	7	7
5	5	5	5	5
6	6	6	6	5
3	3	2	2	2

5	5	5	5	7
4	6	5	5	4
6	6	6	6	6
6	6	6	7	7
7	7	6	7	7
4	2	2	2	5
4	5	5	5	6
6	5	7	6	7
6	6	6	5	7
7	7	7	7	7
4	4	3	3	5
6	5	6	5	6
5	5	5	5	6
3	5	4	3	5
6	6	5	6	6
3	3	5	4	5
4	4	3	3	5
6	6	6	6	6
6	6	6	6	6
7	7	7	7	7
6	6	5	5	6
6	6	6	6	7
7	7	7	7	7
3	3	5	5	3
6	5	7	7	6
6	6	7	6	6
5	6	6	5	6
7	6	7	6	6
6	6	6	6	6
5	5	6	5	6
6	6	6	6	7
7	7	7	7	6
5	5	1	1	4
5	5	6	6	7
5	5	7	6	7
5	5	6	5	5
6	6	7	7	7
7	7	6	7	7
5	6	5	6	6
6	6	6	6	6
6	5	6	6	6
5	5	5	5	5
4	5	6	6	7
5	6	6	6	5
6	6	6	6	6
6	6	6	6	6
7	7	6	6	7
7	7	6	5	7

7-4.Vegetation coverage	7-5.Overall coordination	8-1.Color richness	8-2.Visual effect	8-3.Cultural connotation
5	5	5	5	7
7	7	7	7	7
5	5	5	6	5
6	6	5	5	5
7	6	7	7	6
7	7	7	7	6
6	6	6	5	6
4	4	4	4	4
4	5	5	6	4
6	6	6	6	6
6	5	5	5	5
5	4	5	5	5
3	3	5	3	5
6	6	6	6	6
5	5	5	5	4
5	6	6	5	5
7	7	7	7	7
7	7	6	6	7
5	5	5	5	6
4	5	4	5	6
5	6	6	5	6
5	5	6	6	5
5	5	5	5	5
2	2	3	3	3
5	4	4	4	4
6	6	6	6	7
4	5	4	5	4
5	6	5	6	5
5	4	6	6	6
4	5	5	4	4
4	4	5	5	6
4	5	5	4	5
3	6	5	4	2
3	6	5	6	4
7	6	6	5	5
3	5	5	6	6
4	5	4	5	6
5	5	6	6	6
6	6	5	4	4
6	6	7	6	6
4	5	6	5	5
6	5	5	6	6
6	6	6	6	7
5	6	6	6	5
4	4	4	4	4
5	5	5	5	6
5	5	6	5	6
6	6	6	6	6
6	7	6	6	6
5	5	6	6	6
5	5	5	4	5
5	5	5	5	5
4	5	5	5	4

6	6	7	7	7
5	5	6	6	6
5	5	5	5	5
6	7	6	6	6
6	6	5	5	6
3	3	3	3	5
5	5	6	5	6
5	5	6	6	6
5	5	5	6	5
6	6	6	6	6
5	6	6	6	6
4	4	5	5	5
5	5	4	4	5
6	6	7	6	7
5	6	6	6	7
5	5	6	6	6
4	4	5	5	5
3	3	3	3	3
5	4	5	5	6
6	6	7	4	5
6	6	6	7	6
7	6	6	6	7
6	6	6	6	6
7	6	7	6	6
6	6	6	6	6
4	4	4	5	4
5	5	5	5	5
6	6	6	6	6
6	6	6	6	6
7	7	7	7	7
6	6	6	6	6
6	6	7	7	7
4	6	4	5	5
4	4	4	4	4
5	6	5	5	6
6	6	6	6	6
6	7	7	6	7
7	7	6	7	7
5	5	5	4	4
7	7	7	7	7
6	7	7	6	7
7	7	7	7	7
6	5	6	5	6
6	6	6	6	6
7	6	7	7	7
4	4	4	4	5
6	6	7	6	7
5	5	6	6	6
6	5	6	6	5
5	5	5	5	5
5	6	6	6	6
4	4	4	4	4
5	5	5	5	5
6	6	6	6	6
6	7	6	7	6
6	6	5	6	7

5	4	5	5	6
5	5	5	5	5
6	6	6	5	6
5	5	4	4	6
6	6	6	6	7
5	4	3	3	3
6	5	7	6	7
6	6	7	7	7
4	4	5	4	5
5	6	6	6	5
5	4	5	4	5
6	6	5	5	6
6	6	7	7	5
3	3	5	4	5
7	6	6	7	6
4	4	5	4	5
5	5	6	4	6
5	4	5	4	4
6	6	6	6	6
6	5	6	6	6
6	6	7	5	4
6	6	6	6	6
6	6	6	6	6
5	5	5	5	6
6	5	6	5	6
5	6	6	4	7
7	7	7	6	7
6	5	5	5	6
6	6	6	7	7
6	6	5	7	7
4	5	5	5	6
6	6	7	7	7
6	6	7	6	7
6	6	6	6	6
5	5	5	5	6
5	5	4	5	6
6	6	7	7	7
7	7	4	4	4
4	3	3	2	3
4	4	5	4	5
5	5	5	5	5
6	6	6	6	6
7	7	7	7	7
5	4	5	4	5
6	6	6	6	7
5	6	5	6	6
6	6	6	6	6
6	6	6	6	6
5	5	4	4	4
4	4	5	5	5
7	7	7	7	7
5	7	7	7	7
6	6	7	7	7
6	6	6	5	6
7	7	6	6	6
5	6	6	6	6

5	6	6	6	6
5	6	6	6	5
4	4	6	6	6
5	5	5	5	5
4	4	4	4	4
6	6	5	5	5
6	6	5	5	6
5	6	6	6	6
6	6	6	6	6
5	6	6	5	6
4	4	5	5	4
6	5	6	6	5
5	6	3	4	4
6	5	4	5	6
5	5	7	6	6
6	5	5	5	5
6	5	6	6	5
3	4	5	4	5
6	6	6	6	7
5	6	6	5	6
5	4	5	5	5
4	5	5	5	4
5	5	4	4	5
6	6	5	5	6
4	5	4	4	5
6	7	6	6	6
3	3	3	3	7
4	4	5	5	5
5	6	6	5	6
6	6	6	6	6
6	6	4	4	4
7	7	5	5	6
5	5	5	5	5
6	5	5	6	6
7	6	6	7	5
6	6	6	5	6
5	5	5	5	6
5	4	5	5	6
5	6	6	6	6
5	5	5	5	6
5	5	5	5	5
5	5	1	1	6
6	5	6	6	5
6	6	7	7	6
7	5	6	7	7
5	6	6	6	6
4	4	3	3	3
3	3	3	3	5
5	4	3	5	7
5	5	5	5	6
5	5	6	5	5
6	6	4	4	5
7	7	6	6	7
5	5	6	5	5
5	6	5	6	5
3	3	4	4	4

5	5	4	4	7
4	5	5	5	5
6	6	6	6	6
6	6	6	6	6
7	7	7	7	7
3	2	2	2	2
5	5	5	5	7
5	5	5	6	7
6	6	6	6	7
7	7	7	7	7
5	4	3	3	5
5	6	4	5	6
4	4	6	5	6
5	4	5	5	6
6	6	5	6	6
4	5	4	3	5
3	3	3	3	5
6	6	6	6	7
6	6	7	7	7
7	7	4	5	3
6	6	6	6	6
6	6	6	6	7
7	7	6	6	7
5	3	5	4	4
6	6	6	5	6
6	6	5	5	6
5	5	5	5	6
6	6	6	6	6
6	6	6	5	6
6	5	6	5	6
6	6	6	5	7
7	7	7	7	6
5	5	1	1	5
5	5	6	6	6
5	6	5	5	7
5	5	5	5	6
6	6	6	6	6
7	7	6	7	7
6	6	6	5	6
6	6	6	6	6
5	6	6	5	5
5	5	4	4	3
4	6	6	7	7
6	6	6	6	6
6	6	5	6	6
6	6	6	6	6
6	7	5	5	7
5	5	7	7	7

8-4.Vegetation coverage	8-5.Overall coordination	9-1.Color richness	9-2.Visual effect	9-3.Cultural connotation
6	5	5	5	6
7	7	7	7	7
5	6	5	5	6
5	5	6	5	5
7	7	6	6	7
5	7	4	6	7
5	6	5	5	6
4	4	5	4	5
5	3	2	5	4
6	6	6	6	6
5	5	5	4	4
5	5	5	5	5
3	3	5	4	5
6	6	6	6	6
5	4	4	5	6
5	6	6	6	5
7	7	7	7	5
5	6	7	6	4
5	5	6	6	5
3	4	5	5	3
5	5	6	6	5
6	5	6	5	6
5	5	5	5	5
3	3	2	2	2
5	5	5	5	5
6	6	6	7	6
4	5	4	3	4
6	5	5	7	7
6	6	5	5	4
5	5	5	4	3
5	5	6	6	6
4	4	5	6	5
3	4	6	6	3
4	6	4	6	3
5	5	6	5	4
3	5	6	5	3
3	5	4	5	6
5	5	5	5	5
4	5	4	5	3
6	6	5	5	5
4	5	5	5	5
5	5	5	6	6
5	6	6	6	6
5	5	5	6	5
4	4	4	4	4
5	5	5	6	5
3	3	5	5	5
5	6	6	6	5
6	6	7	7	6
6	5	4	4	2
5	5	4	5	5
5	5	7	6	6
4	4	6	5	5

4	7	4	2	4
4	4	5	5	5
5	5	4	5	5
6	6	5	6	6
5	6	5	6	5
4	5	6	5	5
3	6	7	7	2
4	4	6	6	5
5	4	4	4	4
6	6	6	6	6
4	5	4	4	3
4	4	6	5	4
5	5	5	5	5
6	6	6	6	5
5	5	7	6	3
6	6	6	6	5
5	4	4	5	4
3	3	3	3	3
6	6	4	5	5
4	4	7	7	3
6	6	7	7	6
6	6	6	6	6
6	6	5	6	6
7	7	7	7	7
6	6	6	6	5
4	4	4	4	4
5	5	5	4	4
6	6	6	6	7
6	6	6	6	6
7	7	4	4	4
6	6	6	6	6
7	7	7	7	7
4	4	5	6	5
4	4	6	5	3
5	5	6	6	5
6	5	6	6	6
6	7	7	6	7
7	7	7	7	7
5	5	5	4	4
7	6	7	7	7
6	6	7	7	5
7	7	7	7	7
6	5	5	5	5
6	6	6	5	6
7	7	7	7	7
4	4	6	6	5
6	6	7	7	6
6	6	5	6	6
6	6	5	5	5
5	5	6	5	5
5	6	6	6	6
4	4	4	4	4
5	5	5	5	4
6	6	6	6	6
6	6	6	5	6
7	5	6	5	4

4	5	5	5	4
5	5	6	6	6
6	6	6	6	6
4	4	5	5	4
5	6	7	7	5
3	3	4	4	4
6	6	7	6	5
7	7	7	7	7
4	4	4	3	4
4	6	4	6	6
5	4	5	5	4
6	6	6	7	5
6	7	5	5	4
3	3	4	4	2
6	7	6	7	6
4	4	6	6	5
5	5	6	4	6
5	4	5	5	5
6	6	7	7	6
5	6	6	6	6
5	5	7	7	7
6	6	6	6	6
7	7	7	7	7
5	5	6	5	4
5	5	5	5	5
6	6	5	6	5
7	6	6	7	6
5	5	5	4	5
6	7	6	5	5
7	7	6	6	7
5	4	6	6	6
6	6	6	7	7
6	6	7	6	4
5	5	5	5	4
5	5	5	5	4
5	5	3	5	4
6	6	7	7	6
7	7	7	7	7
3	3	3	3	4
4	3	5	5	4
5	5	7	7	6
6	6	6	6	6
7	7	7	7	6
4	4	5	4	5
5	6	6	6	4
5	6	5	5	6
6	6	6	6	6
7	6	6	6	6
4	4	4	3	4
4	4	7	7	7
7	7	7	7	7
7	7	7	7	7
7	7	7	7	7
6	6	6	5	6
6	6	6	6	6
5	6	6	6	5

5	5	4	5	5
5	6	5	6	6
6	5	6	6	5
5	5	5	5	5
4	4	5	4	4
6	5	5	6	5
5	5	5	5	5
5	6	6	5	6
6	6	6	6	5
5	5	5	5	5
4	4	5	5	4
5	4	6	6	4
4	4	4	5	5
5	5	6	5	5
6	6	5	4	4
5	5	5	6	5
6	5	7	6	6
5	4	6	6	6
6	6	6	6	5
5	6	6	6	6
4	4	4	4	6
5	5	6	6	4
4	4	5	5	4
5	6	6	5	5
5	4	3	5	5
4	6	7	6	7
7	3	3	4	6
5	5	5	4	4
4	5	5	5	6
6	6	6	6	6
4	4	5	5	6
5	3	7	7	7
5	5	5	4	4
5	6	5	6	5
7	6	6	6	7
6	6	4	5	5
5	5	4	4	3
4	4	6	6	6
6	6	5	4	4
4	4	5	4	6
5	5	5	5	6
2	1	6	7	6
6	6	6	5	6
6	6	7	6	6
6	5	7	7	6
5	5	5	5	5
3	3	4	4	4
3	3	4	4	5
5	5	5	5	4
5	5	6	6	5
5	5	6	5	6
4	4	4	4	4
7	7	7	7	7
5	6	5	5	5
7	6	5	6	7
4	4	4	4	4

4	4	4	4	7
4	5	5	4	5
6	6	6	6	6
6	6	6	5	5
7	7	6	7	7
2	2	2	2	2
3	5	5	6	6
5	5	6	5	6
6	6	5	6	7
7	7	7	7	6
4	4	2	2	4
5	6	7	7	2
4	4	6	5	6
5	5	5	5	4
6	6	5	6	6
3	3	3	3	5
3	3	5	4	5
6	6	6	6	4
7	7	6	6	6
5	3	7	7	7
6	6	6	5	6
6	6	6	6	7
6	6	6	6	6
4	3	4	3	5
5	5	7	7	4
5	6	5	5	5
5	5	5	6	4
5	5	6	6	6
6	6	6	6	4
6	6	6	6	5
6	6	6	6	6
6	6	6	7	6
5	5	5	5	5
5	6	5	5	4
6	5	6	6	4
5	5	6	6	5
6	5	5	5	5
7	7	6	6	7
6	6	5	5	4
5	6	6	5	4
4	4	4	5	3
4	4	5	5	5
4	5	6	7	2
5	6	6	6	4
6	6	6	6	5
6	6	4	5	4
5	5	5	4	6
7	7	5	5	5

9-4.Vegetation coverage	9-5.Overall coordination	10-1.Color richness	10-2.Visual effect	10-3.Cultural connotation
5	5	5	5	5
6	7	7	7	7
5	5	5	5	5
5	5	6	6	6
6	6	7	6	7
4	6	6	5	6
5	6	5	6	5
4	5	5	5	5
5	6	3	5	3
6	6	6	7	6
4	4	5	5	5
5	5	5	5	5
4	4	5	4	5
6	6	6	6	6
5	5	4	4	3
5	6	6	5	6
7	7	7	6	7
5	6	7	7	7
5	6	6	5	5
3	5	4	2	5
5	6	5	5	6
6	6	6	5	5
5	5	5	5	5
2	2	2	2	2
5	5	4	4	4
6	6	7	6	7
6	4	3	4	5
5	5	7	6	6
2	3	7	7	7
3	3	5	5	5
6	6	5	6	5
6	6	6	5	5
6	6	7	7	7
5	5	6	5	7
5	5	5	4	6
5	5	6	6	6
3	4	3	5	6
5	5	5	5	5
3	5	4	4	5
6	5	6	6	6
4	5	5	5	5
5	5	5	5	5
7	6	6	6	7
6	6	7	7	7
4	4	5	4	5
5	5	5	5	6
5	5	6	6	5
6	5	7	7	7
7	7	6	5	7
2	2	7	7	7
5	5	4	5	5
6	6	6	5	6
4	5	5	5	5

4	4	7	7	7
5	4	6	6	6
5	5	5	5	5
6	6	6	6	7
6	6	5	6	6
6	6	6	6	7
7	7	6	6	6
5	5	5	5	6
5	4	5	5	5
6	6	6	6	6
5	5	6	6	6
4	4	5	5	5
4	4	4	4	5
6	6	5	6	6
6	5	6	5	6
6	6	6	6	6
4	4	5	5	5
3	3	4	4	4
5	5	5	5	5
7	5	6	5	5
6	6	6	5	7
6	6	6	6	7
6	6	5	6	6
7	7	7	7	6
6	6	6	6	6
4	4	5	5	6
4	4	5	4	4
6	6	6	6	6
6	6	6	6	6
4	4	5	5	5
6	6	6	6	6
7	7	7	7	7
5	5	3	5	5
3	3	4	4	4
5	6	5	5	5
6	6	6	6	6
6	7	7	6	7
6	7	7	6	7
5	5	5	5	5
7	7	7	7	7
7	7	6	6	7
7	7	7	7	7
5	5	5	6	7
6	6	6	6	6
7	7	7	6	7
6	6	4	5	5
6	6	7	6	7
6	6	5	6	6
6	6	5	6	6
6	5	5	5	6
5	6	5	6	5
4	4	4	4	4
5	6	5	5	6
6	6	6	6	6
6	6	6	6	6
6	6	6	5	7

4	6	5	5	6
6	6	5	5	5
6	6	6	6	6
4	4	4	5	5
6	6	5	5	6
4	4	4	4	4
7	6	7	6	6
5	7	7	6	7
4	4	5	5	4
4	6	6	6	4
4	5	6	6	5
6	7	4	6	7
6	5	5	5	6
4	4	4	3	6
6	7	7	6	7
5	5	5	4	5
5	5	5	3	5
5	5	5	5	5
7	7	6	6	7
3	3	6	6	6
7	7	7	7	7
6	6	7	7	7
7	7	7	7	7
5	5	5	5	6
5	5	6	5	5
6	6	6	5	7
7	6	7	7	7
4	4	5	6	6
7	7	7	7	7
6	5	6	6	7
6	6	5	6	7
6	6	7	7	7
6	6	7	6	6
5	5	6	6	6
5	4	5	5	5
5	5	6	5	6
6	6	7	6	6
7	7	7	4	7
4	3	3	4	5
6	6	5	4	5
7	7	6	6	6
6	6	6	6	6
7	6	6	6	7
4	4	4	4	5
6	5	5	5	6
6	5	6	6	6
6	6	6	6	6
6	7	7	6	6
3	4	4	4	4
7	6	7	7	7
7	7	7	7	7
7	7	7	7	7
7	7	7	7	7
6	6	6	5	6
6	6	7	7	7
5	6	6	5	6

5	5	5	5	5
6	5	6	5	6
5	5	5	5	5
5	5	5	5	5
5	4	4	4	4
6	5	6	6	6
5	5	5	5	6
6	6	6	6	6
6	6	6	6	6
5	5	6	5	5
5	4	5	5	5
6	5	5	5	6
4	5	5	5	5
5	5	5	4	5
4	4	5	6	6
6	5	5	5	6
7	6	6	6	5
7	6	6	5	5
6	7	6	6	7
5	6	6	5	5
4	5	5	5	5
3	5	6	5	6
4	5	4	4	5
6	5	6	6	5
3	3	6	5	7
6	4	6	6	6
5	4	5	4	7
4	4	4	4	4
6	7	4	4	6
6	6	6	6	6
5	5	6	5	6
7	7	7	7	7
4	4	5	5	5
6	6	6	5	7
5	6	5	7	5
5	5	5	6	4
3	2	4	4	6
4	4	5	5	6
5	5	5	5	6
4	4	5	4	6
5	5	6	6	6
6	6	6	6	6
6	6	6	5	6
6	7	6	6	7
6	6	6	6	6
6	5	5	5	6
4	4	3	3	3
3	3	3	3	5
3	2	7	6	7
6	5	5	6	7
5	5	5	6	5
4	4	4	5	4
7	7	6	6	7
5	5	5	5	6
4	6	5	6	5
4	4	4	4	4

4	4	5	5	7
4	4	5	5	5
6	6	6	6	6
5	6	6	6	7
7	7	7	7	7
2	2	2	2	2
4	4	5	4	7
6	6	6	6	6
5	5	5	5	6
6	7	7	7	7
4	4	4	4	4
7	7	7	7	7
5	5	5	5	6
5	5	3	3	5
5	5	5	6	6
3	3	6	5	6
4	4	6	5	5
6	6	6	6	7
6	6	7	7	7
7	7	3	6	3
6	6	6	6	6
6	6	6	6	7
6	6	7	6	6
4	4	5	4	3
6	5	5	5	6
6	6	6	5	6
5	5	6	6	7
6	6	7	6	6
6	6	6	6	6
5	6	6	6	6
6	5	6	6	7
7	7	7	6	7
5	5	5	5	5
5	6	6	6	7
5	6	6	6	7
5	6	5	5	6
5	5	6	7	7
7	7	6	7	7
5	4	5	6	6
6	6	6	6	6
3	3	5	5	6
5	5	3	3	3
3	3	6	6	7
6	6	6	6	6
6	6	6	6	6
5	3	6	6	6
6	5	6	6	6
5	5	6	6	6

10-4.Vegetation coverage	10-5.Overall coordination	11-1.Color richness	11-2.Visual effect	11-3.Cultural connotation
5	5	5	5	5
7	7	7	7	7
5	5	5	5	5
6	6	6	6	6
6	6	6	7	6
7	5	4	6	6
5	6	6	5	6
5	5	5	5	5
5	4	3	4	5
5	6	6	6	6
5	5	5	5	4
5	5	6	5	5
4	4	5	4	5
6	6	6	6	6
4	4	4	4	4
6	5	6	5	6
7	7	7	7	7
7	7	7	7	7
6	5	5	5	5
6	4	3	3	5
5	5	5	5	6
6	6	5	6	6
5	5	4	4	4
2	2	2	2	2
4	4	5	5	5
6	7	5	6	7
4	6	4	4	4
5	7	5	5	7
7	7	6	6	6
4	5	5	4	4
5	6	4	4	6
5	6	5	4	5
7	7	6	5	4
5	6	6	4	6
5	5	4	4	4
5	6	6	6	6
4	3	4	3	6
5	5	5	5	5
3	4	4	4	3
6	6	6	6	6
4	5	5	5	5
5	5	5	5	5
7	7	6	6	7
7	7	6	6	6
4	4	4	4	4
5	5	5	5	6
6	5	5	6	6
6	7	6	6	7
5	5	6	6	6
7	6	5	5	6
5	4	5	5	4
6	6	6	6	6
4	5	5	4	5

6	7	3	2	4
5	6	5	5	5
4	4	5	5	5
6	6	6	6	7
5	6	5	6	5
7	6	5	4	5
2	5	6	6	6
5	5	6	5	5
5	5	5	5	5
6	6	6	6	6
5	6	5	5	6
5	4	5	4	4
5	5	5	4	5
6	6	6	6	6
6	5	6	6	6
6	5	5	5	7
5	5	5	5	5
4	4	4	4	5
5	5	5	5	5
5	4	4	4	5
6	6	7	5	6
6	6	6	6	6
6	6	6	6	6
6	6	7	7	7
6	6	6	6	6
6	6	5	5	5
5	5	5	4	4
6	6	6	6	6
6	5	6	5	6
5	5	6	6	6
6	6	6	6	6
7	7	7	7	7
5	3	4	4	5
4	4	5	5	5
5	5	6	6	5
6	6	6	6	6
6	7	7	6	7
7	7	7	7	7
4	4	5	4	5
7	7	7	7	7
6	6	7	6	7
7	7	7	7	7
5	5	5	6	5
5	6	6	6	6
7	6	7	7	7
5	4	4	4	5
7	7	7	6	7
6	6	5	5	5
6	6	5	6	6
6	5	5	6	5
5	6	6	6	5
4	4	4	4	4
5	5	5	5	6
6	6	6	6	6
5	6	6	6	5
6	6	6	5	7

5	5	5	5	5
5	5	6	6	6
6	6	6	6	6
5	4	4	4	5
6	6	6	6	7
5	4	4	4	5
7	6	7	7	7
6	6	6	6	6
4	4	5	4	5
6	6	5	6	6
5	5	6	5	6
5	7	6	6	5
5	5	5	5	6
5	5	5	4	4
7	7	6	6	7
4	4	5	4	5
5	5	5	3	5
5	5	5	5	5
6	6	6	6	6
6	6	6	6	6
7	7	6	6	6
7	7	7	7	7
7	7	7	7	7
5	5	5	5	6
6	6	6	5	6
6	5	6	5	7
6	7	6	6	7
6	5	5	5	6
6	6	6	6	7
7	6	6	7	6
5	4	5	6	5
6	6	7	7	7
6	6	6	6	5
6	6	6	6	6
5	5	5	4	5
5	6	4	5	5
6	6	7	7	7
6	5	7	4	7
3	3	4	3	3
5	5	5	5	5
6	6	5	5	6
6	6	6	6	6
7	7	7	7	7
4	4	5	4	5
5	6	5	5	6
6	6	5	5	6
6	6	6	6	6
6	6	6	6	7
4	4	4	3	5
7	7	7	7	7
7	7	7	7	7
7	7	7	7	7
7	7	6	6	6
6	6	6	5	6
7	7	7	7	7
6	5	6	6	6

6	5	6	6	5
6	5	5	6	6
6	5	5	4	6
5	5	5	5	5
4	4	5	5	5
6	6	6	6	6
5	5	5	6	5
5	5	6	5	6
6	6	6	6	6
5	5	5	3	6
4	4	5	4	5
5	6	6	6	6
5	6	5	5	5
6	5	5	6	5
5	6	4	4	6
6	6	5	6	5
6	7	6	6	7
6	5	7	6	6
6	5	6	7	7
4	6	6	6	5
5	5	5	5	5
6	6	4	4	5
4	5	4	4	4
6	6	5	5	5
5	5	5	4	5
6	6	7	7	6
4	3	3	3	6
4	4	4	4	4
5	6	5	5	6
6	7	6	6	6
5	5	5	6	6
7	7	6	6	4
5	6	5	4	5
6	6	6	6	6
6	7	6	5	7
5	6	4	5	6
4	3	4	4	6
6	6	5	4	7
5	5	5	4	5
4	4	4	6	6
5	5	5	6	5
6	6	6	5	6
6	5	6	6	5
7	7	7	6	6
7	6	7	6	6
5	5	6	5	6
3	3	4	4	4
3	3	3	3	5
6	5	5	6	7
6	6	5	5	6
5	6	6	5	5
5	4	5	4	5
7	7	7	7	7
4	5	5	5	5
5	6	4	5	6
4	4	4	4	4

5	5	4	4	6
5	5	4	5	6
6	6	6	6	6
6	6	6	6	6
7	7	7	7	7
5	2	2	2	2
4	4	5	5	7
5	5	6	6	6
6	6	4	4	6
7	7	7	7	7
4	4	4	4	4
7	7	6	5	7
5	4	5	5	5
5	7	5	5	5
6	6	6	6	6
3	3	5	5	3
4	4	3	3	5
6	6	5	6	6
7	7	7	7	7
4	4	7	5	7
6	6	6	6	6
6	6	7	6	7
6	6	7	7	7
5	4	4	3	5
5	5	4	4	6
5	6	6	6	6
6	6	5	5	6
6	6	7	6	6
6	6	6	6	5
5	4	6	5	5
6	6	6	6	7
7	7	7	6	6
5	5	5	5	5
6	6	5	5	6
5	5	7	6	7
6	6	6	6	6
6	6	7	6	5
7	7	6	7	7
5	6	5	5	6
5	5	6	6	6
5	5	6	6	5
5	4	5	5	5
5	5	6	6	7
5	6	6	6	5
5	6	6	6	6
6	6	5	5	6
7	7	6	5	7
6	6	6	6	7

11-4.Vegetation coverage	11-5.Overall coordination	12-1.Color richness	12-2.Visual effect	12-3.Cultural connotation
5	5	5	5	5
7	7	6	6	7
5	5	5	5	5
6	6	6	6	6
7	7	7	7	6
4	6	4	6	6
6	5	5	5	5
5	5	5	5	5
3	5	3	2	5
6	6	6	6	6
5	5	5	4	5
6	6	6	5	6
4	4	5	4	5
6	6	6	6	6
4	4	4	4	4
6	6	6	5	5
7	7	7	5	7
7	7	6	6	7
5	5	5	5	5
4	4	4	5	6
5	5	6	5	6
5	5	6	6	5
4	4	5	5	5
2	2	2	3	2
5	5	5	5	5
5	6	6	6	6
5	4	5	3	5
5	6	6	6	6
5	6	6	4	6
4	4	4	3	4
4	5	4	4	6
5	4	6	4	6
6	6	4	4	5
4	6	4	2	7
4	4	4	4	6
5	6	6	6	6
4	4	4	5	7
5	5	4	4	5
5	5	6	4	4
6	6	5	5	5
4	5	5	5	5
5	5	5	5	5
7	6	6	5	7
6	6	6	7	7
4	4	4	4	4
5	5	5	5	6
6	7	6	6	5
6	6	6	6	7
6	6	6	6	6
5	5	5	6	6
5	5	5	5	4
6	6	7	6	6
4	5	5	5	5

4	4	2	2	5
5	5	5	4	5
5	5	4	4	5
6	6	6	6	7
5	6	6	5	6
6	6	5	5	6
5	5	5	2	5
4	4	4	5	5
5	5	5	5	5
6	6	6	6	6
5	5	5	6	5
4	4	5	5	5
4	4	4	4	4
6	6	5	5	6
5	6	7	7	7
5	5	6	6	7
5	5	5	5	6
4	4	4	4	5
5	5	4	5	5
4	5	5	4	6
5	6	7	6	5
6	6	6	6	6
6	6	6	6	6
7	7	7	6	6
6	6	6	6	6
5	5	5	5	5
4	4	5	5	4
6	6	6	6	6
6	6	6	6	7
6	6	6	6	6
6	6	6	6	6
7	7	5	5	6
5	6	5	4	5
5	5	5	5	6
5	5	5	5	6
6	6	6	6	6
6	7	7	7	7
7	7	7	7	7
4	5	5	5	4
7	7	7	7	6
7	7	6	7	7
7	7	7	7	7
5	5	6	5	5
5	6	6	6	6
7	7	6	6	7
4	4	4	5	5
6	6	7	7	7
5	5	6	6	6
5	6	6	6	6
5	5	5	5	6
5	6	7	6	5
4	4	4	4	4
5	5	5	5	6
6	6	6	6	6
6	6	6	5	6
6	6	6	7	7

5	5	5	4	6
6	5	6	5	5
6	6	6	6	6
4	4	4	5	5
6	6	6	6	7
4	4	5	4	5
7	6	7	7	7
6	6	7	6	6
5	5	4	4	5
5	5	5	6	4
5	5	5	5	5
6	6	5	6	7
5	5	5	6	5
4	4	4	4	6
7	6	6	7	7
4	4	5	5	5
5	4	5	3	5
5	5	5	4	5
6	6	5	6	6
6	6	6	5	6
6	6	6	6	6
7	7	7	7	7
7	7	6	6	7
5	5	6	5	6
5	5	5	6	6
5	5	6	6	7
7	6	6	6	7
5	5	4	4	6
6	6	6	7	7
5	7	7	7	7
5	6	5	5	6
6	6	7	7	7
6	6	6	6	6
5	5	6	5	6
5	4	5	4	4
3	4	5	5	6
6	6	7	6	7
7	6	7	6	7
4	3	2	4	5
4	3	5	6	5
5	5	7	7	7
6	6	6	6	6
7	7	7	7	7
5	4	5	4	5
5	5	5	5	6
5	5	6	6	6
6	6	6	6	6
6	6	7	6	6
4	4	5	4	5
7	7	7	6	6
7	7	7	7	7
7	7	7	7	7
6	6	6	6	6
6	6	5	5	6
7	7	6	6	6
6	6	5	5	6

5	5	5	5	5
5	5	5	6	6
5	5	5	6	6
5	5	5	5	5
5	5	5	4	4
6	6	6	6	6
5	5	5	5	6
5	6	6	6	6
6	6	6	6	6
4	4	5	5	5
4	5	5	4	5
5	4	5	5	7
5	6	6	6	6
5	5	5	6	6
5	5	6	6	6
5	5	5	6	6
6	7	6	7	7
6	6	6	5	6
6	6	6	6	6
6	5	6	5	6
5	5	5	5	5
4	4	5	4	5
4	4	4	4	5
6	5	5	5	5
5	5	3	4	6
6	6	6	7	6
3	5	3	3	6
4	4	4	4	4
6	6	5	6	6
6	6	6	6	6
5	5	4	6	6
6	7	6	6	7
5	5	4	4	5
6	7	6	6	6
6	7	6	6	5
5	5	5	6	6
4	3	5	5	7
5	6	5	4	6
5	5	5	5	6
5	5	4	6	6
5	4	5	4	5
5	6	6	6	6
7	5	7	7	7
6	6	7	6	6
7	7	7	6	7
6	6	6	6	6
4	4	4	4	4
3	3	3	3	5
5	6	5	4	6
6	5	5	5	7
6	5	6	5	5
4	5	5	5	4
7	7	6	6	7
5	5	5	5	5
4	6	6	4	6
4	3	4	4	4

4	4	4	4	5
5	5	4	3	6
6	6	6	6	6
5	5	6	5	6
7	7	7	7	7
5	2	2	2	5
3	5	4	4	7
5	5	5	4	6
5	5	4	4	7
7	7	7	7	7
4	4	4	4	4
6	6	5	6	7
3	3	5	6	6
6	6	5	6	6
6	6	6	5	6
5	3	5	5	6
3	3	3	3	5
5	5	6	6	6
7	7	7	7	7
3	3	7	7	7
5	6	5	6	6
6	6	6	7	6
7	7	6	6	6
5	5	5	5	4
5	5	4	5	6
6	6	6	5	6
5	5	6	5	7
6	5	7	6	6
6	5	5	5	6
6	6	6	5	6
5	5	6	7	7
7	7	7	7	7
5	5	1	1	1
5	5	5	5	7
5	6	7	6	7
6	6	6	6	6
5	6	6	6	5
7	7	6	6	7
4	5	4	4	6
6	6	6	6	6
5	5	6	6	6
5	5	5	5	5
6	6	7	7	7
5	6	6	6	6
6	6	6	5	6
5	5	7	6	7
6	6	5	5	6
6	5	7	7	7

12-4.Vegetation coverage	12-5.Overall coordination	13-1.Color richness	13-2.Visual effect	13-3.Cultural connotation
5	5	5	5	6
6	7	7	7	7
5	5	5	5	5
6	6	6	6	6
7	7	7	7	7
4	6	4	6	7
5	5	6	6	6
5	5	4	4	4
5	3	1	1	5
6	6	6	6	6
5	5	6	6	6
5	5	5	5	5
4	4	5	4	5
6	6	6	6	6
4	4	4	4	4
6	5	4	5	4
7	7	7	7	7
7	7	7	6	6
5	5	5	5	5
4	5	5	4	4
5	5	5	5	5
5	4	4	6	5
5	5	5	5	5
3	2	3	2	3
5	5	6	5	5
6	6	6	6	7
5	4	5	4	5
5	5	6	6	4
4	7	6	4	6
3	3	5	4	3
4	5	5	5	6
5	4	5	4	4
4	5	4	6	6
5	4	6	5	6
4	6	5	4	4
5	5	6	6	6
4	5	4	3	6
4	4	5	5	5
6	4	6	6	6
6	5	6	6	6
5	5	5	5	5
5	5	6	5	5
6	7	6	6	7
7	7	6	6	6
4	4	4	4	4
5	5	5	5	6
6	4	6	5	4
6	6	7	7	7
6	6	6	6	6
5	5	6	5	6
5	5	4	5	4
6	6	7	6	6
4	5	6	5	5

4	4	7	7	7
4	4	5	5	5
4	4	5	5	5
6	6	6	6	7
6	6	6	5	6
5	5	5	5	5
2	4	6	6	6
5	5	5	5	5
5	5	6	6	5
6	6	6	6	6
6	5	6	5	5
4	4	5	5	4
4	4	5	5	5
6	6	6	6	6
6	6	7	6	7
5	5	6	5	7
5	5	6	6	6
4	4	4	4	5
4	5	5	5	5
6	5	5	6	6
6	5	6	6	6
6	6	6	6	6
6	6	6	6	6
7	7	7	7	7
6	6	6	6	6
5	6	6	6	6
4	5	5	4	4
6	6	6	6	6
6	4	7	6	5
6	6	7	7	7
6	6	6	6	6
5	6	5	6	6
5	4	5	5	5
4	4	5	5	5
5	5	5	5	5
6	6	6	6	6
6	7	7	6	7
7	7	7	7	7
5	4	5	5	4
7	7	7	7	7
6	6	7	7	7
7	7	7	7	7
5	5	6	5	6
6	6	6	5	5
7	7	7	7	6
4	4	5	4	4
7	6	7	7	7
6	6	5	5	5
6	6	6	6	5
5	5	5	5	5
5	6	6	5	6
4	4	4	4	4
5	5	5	5	5
6	6	6	6	6
6	6	6	6	6
6	6	4	5	6

4	4	5	5	5
6	6	5	6	6
6	6	6	6	6
4	4	4	5	6
5	5	6	7	7
4	4	4	4	4
7	6	7	7	6
5	5	6	6	6
4	4	5	5	5
5	5	5	4	6
5	4	5	4	5
6	5	6	6	6
6	6	6	6	6
4	5	4	5	5
7	6	6	7	6
4	4	5	5	5
5	4	5	3	5
5	5	4	4	5
6	6	6	6	7
5	5	5	5	5
6	6	6	6	4
7	7	7	7	7
7	7	7	7	7
5	5	5	5	5
5	5	5	5	6
5	6	5	6	7
7	6	7	6	7
4	4	5	5	6
7	6	6	7	7
7	7	7	7	7
5	4	5	6	5
6	6	7	7	7
6	6	6	6	6
5	5	5	5	6
4	4	5	5	4
4	5	7	6	5
6	6	7	6	6
7	7	7	6	7
3	2	4	3	5
5	5	5	5	6
6	6	7	7	7
6	6	6	6	6
7	7	6	6	7
5	4	5	4	5
6	6	6	6	6
5	6	6	6	6
6	6	6	6	6
7	6	6	7	7
4	4	4	4	4
7	7	6	6	4
7	7	7	7	7
7	7	7	7	7
6	6	6	6	6
6	6	6	5	6
6	6	7	7	7
6	5	6	6	6

5	5	5	5	6
5	6	5	6	5
5	5	5	3	5
5	5	5	5	5
4	4	5	5	5
6	6	6	6	6
5	5	7	7	7
5	5	6	5	6
6	6	6	6	6
5	5	5	5	5
4	4	5	4	5
5	5	6	6	7
6	6	6	6	6
6	5	1	1	5
6	6	5	5	6
5	5	5	5	5
6	6	6	6	7
5	4	6	5	5
6	6	6	6	6
6	6	7	7	7
5	5	5	5	5
4	5	4	5	4
4	5	5	4	4
5	5	5	6	5
3	3	4	4	4
5	5	6	7	6
3	3	2	3	7
4	4	4	4	4
6	6	5	5	6
6	6	6	6	6
5	5	6	5	6
6	5	6	7	7
4	5	5	5	5
6	6	6	5	6
7	5	6	5	6
5	5	5	6	5
4	4	4	4	5
4	5	6	5	6
5	5	5	5	6
6	6	4	6	6
5	5	6	5	6
6	6	6	5	5
7	7	7	7	7
6	7	7	7	7
7	6	6	6	6
6	6	6	6	6
4	4	4	4	4
3	3	5	4	5
6	5	5	6	6
6	5	6	5	6
5	5	6	5	5
4	4	4	5	5
7	7	6	6	7
5	5	5	5	5
4	4	6	5	6
4	5	3	3	4

4	4	4	4	4
4	3	5	5	5
6	6	6	6	6
6	6	6	6	7
7	7	6	6	7
5	2	2	2	5
4	4	5	5	7
4	4	6	5	5
5	5	5	5	7
7	7	7	7	7
4	4	4	4	4
4	5	6	7	7
3	3	6	5	4
6	6	5	5	5
6	5	6	7	6
3	3	5	5	6
3	3	4	4	5
6	6	5	5	6
7	7	7	7	7
7	7	5	4	5
6	6	6	6	6
6	6	6	7	7
6	6	6	6	6
4	4	3	4	4
5	4	4	5	7
6	6	6	5	5
5	6	5	6	7
6	6	7	6	6
6	5	5	5	5
5	6	6	5	6
6	5	6	6	7
6	7	7	7	7
4	4	5	5	5
5	5	5	6	6
5	4	7	7	7
6	6	6	5	6
5	5	5	7	6
7	7	6	6	7
5	4	6	6	7
5	5	6	6	6
5	5	5	6	6
5	5	5	5	5
6	7	6	5	6
4	5	6	6	6
6	6	5	6	6
6	6	6	6	6
6	6	7	6	6
7	7	6	6	7

13-4.Vegetation coverage	13-5.Overall coordination	14-1.Color richness	14-2.Visual effect	14-3.Cultural connotation
5	5	5	5	6
7	7	7	7	7
5	5	5	5	5
6	6	6	6	6
6	7	7	7	6
6	6	6	5	6
6	6	6	6	6
4	4	4	4	4
2	2	5	6	6
5	5	6	6	6
6	6	6	6	6
4	5	6	5	5
4	4	4	5	5
6	6	6	6	6
4	4	4	4	4
6	4	4	5	4
7	6	6	6	6
6	6	7	7	7
5	5	5	5	5
3	4	5	4	5
5	5	6	6	6
4	5	5	6	5
5	5	5	5	6
2	3	3	4	3
5	4	5	4	4
6	6	6	6	6
4	5	5	3	5
6	6	4	5	6
6	7	6	6	6
4	4	5	5	3
5	5	6	6	6
4	3	7	6	5
3	4	7	7	7
6	5	5	6	6
4	4	4	4	4
5	5	6	6	6
5	3	4	6	4
5	5	5	5	5
6	6	5	5	6
6	6	6	6	6
4	4	5	5	5
5	5	5	5	5
6	6	7	7	7
6	6	6	6	5
4	4	4	4	4
5	5	5	5	6
5	5	6	5	5
6	6	7	7	7
6	6	6	6	6
5	6	7	7	7
5	5	5	5	5
6	6	7	7	7
4	5	6	5	5

7	7	5	5	5
4	4	6	6	6
4	5	6	6	6
6	6	6	6	7
6	6	6	5	5
5	5	5	6	5
2	2	6	6	6
5	5	6	6	6
6	5	7	7	6
6	6	6	6	6
6	4	4	5	6
4	4	5	5	5
5	5	5	5	5
6	6	7	6	6
5	6	7	7	6
5	5	7	7	7
5	5	6	6	6
4	4	4	4	5
5	5	6	6	6
4	4	7	7	7
5	6	7	7	7
6	6	7	7	6
6	6	6	6	6
7	7	7	7	7
6	6	6	6	6
6	6	6	5	6
4	5	5	4	4
6	6	6	6	6
5	5	6	6	6
7	7	7	7	7
6	6	6	6	6
5	6	6	6	6
6	6	6	6	6
5	5	6	5	5
5	5	5	5	5
6	6	6	6	6
6	7	7	7	7
7	7	7	7	7
5	4	5	5	5
7	7	7	7	7
6	6	7	7	7
7	7	7	7	7
5	6	5	6	6
5	6	6	6	6
7	7	7	7	7
4	4	4	4	5
6	7	7	6	7
5	5	6	5	5
6	6	6	6	6
6	5	5	5	5
6	5	6	5	5
4	4	4	4	4
5	5	5	5	6
6	6	6	5	6
7	6	6	5	7
5	5	7	6	7

4	5	5	5	6
5	5	5	5	6
6	5	5	6	6
4	4	5	4	5
5	6	7	7	7
4	4	6	6	6
6	6	7	7	7
5	6	7	7	7
5	4	4	5	5
5	5	5	5	6
5	5	5	5	6
6	6	6	7	6
6	6	6	7	6
5	3	6	6	5
6	7	7	6	7
5	5	6	7	6
5	4	6	4	5
4	5	5	5	5
6	6	7	7	7
4	4	6	6	5
6	6	7	7	7
7	7	7	7	7
7	6	7	7	7
5	5	5	5	6
5	6	6	6	6
6	6	6	6	7
6	6	7	7	7
4	4	7	7	7
6	7	7	7	6
7	7	7	7	6
6	5	6	6	6
7	6	7	7	7
6	6	7	6	6
5	5	6	6	6
5	4	6	5	6
5	4	6	5	6
6	6	7	6	6
6	6	7	7	7
4	3	4	3	5
4	5	4	6	3
7	7	7	7	7
6	6	6	6	6
7	7	7	6	7
5	5	5	5	5
6	6	6	6	5
5	6	5	5	6
6	6	6	6	6
6	7	7	7	6
4	4	4	4	4
6	6	5	4	7
7	7	7	7	7
7	7	7	7	7
6	6	6	6	6
6	6	6	5	6
7	7	7	7	7
6	5	5	6	6

6	5	6	6	6
5	6	6	5	6
4	4	6	6	5
5	5	5	5	5
5	5	6	6	5
6	6	6	6	6
7	7	7	7	7
6	6	6	5	6
6	6	6	6	6
5	5	5	5	5
4	5	4	5	4
5	5	7	7	7
6	6	6	6	6
1	1	5	5	5
5	5	5	5	6
5	5	6	5	6
7	6	6	7	6
4	4	6	5	4
6	6	6	6	6
6	6	7	6	7
5	5	5	5	5
5	4	5	5	6
4	4	5	5	5
6	5	6	6	5
4	4	4	4	4
7	7	6	5	5
3	4	3	3	6
4	4	4	4	4
5	5	6	6	6
6	6	6	6	6
5	5	5	4	5
7	7	7	7	7
5	5	6	6	6
6	6	6	5	6
7	6	6	5	6
6	5	6	5	6
4	3	4	4	5
4	4	6	6	7
5	5	5	5	6
5	5	4	5	6
4	5	7	6	6
5	5	7	7	7
7	7	7	7	7
6	6	7	6	7
7	7	7	7	7
6	6	6	6	6
4	4	4	4	4
3	4	4	4	5
5	4	6	5	6
5	5	6	5	7
5	5	6	6	6
5	5	5	5	5
7	7	7	7	7
5	5	5	5	5
7	5	3	5	5
4	4	3	4	3

4	4	4	4	4
6	6	6	6	6
6	6	6	6	6
6	6	6	6	6
7	6	7	7	7
2	2	6	6	5
3	4	5	6	7
6	6	7	7	7
5	5	6	6	7
6	6	7	7	7
4	4	4	4	4
5	4	7	7	7
5	3	5	6	6
6	6	6	6	6
6	6	6	6	5
3	3	6	4	4
4	4	5	5	5
6	6	6	6	6
7	7	7	7	7
5	6	7	6	4
6	6	6	6	6
6	6	7	7	6
6	6	6	6	6
4	4	4	5	5
3	5	7	7	7
5	6	7	6	6
5	6	6	6	6
6	6	7	6	6
5	5	6	6	5
6	6	6	5	6
6	6	6	7	6
7	7	7	7	7
5	5	5	5	5
5	5	6	7	6
6	6	6	6	7
5	5	6	6	6
5	5	6	7	7
6	6	6	7	7
6	6	6	6	6
6	6	6	6	6
5	5	6	6	5
5	5	4	4	4
6	5	7	7	7
5	5	6	6	6
5	6	6	5	6
6	6	6	6	6
6	6	6	5	6
6	6	7	7	7

14-4.Vegetation coverage	14-5.Overall coordination	15-1.Color richness	15-2.Visual effect	15-3.Cultural connotation
5	5	5	5	6
7	7	7	7	7
5	5	5	5	5
6	6	6	6	5
7	7	7	6	7
7	7	6	5	6
6	6	6	6	6
4	4	4	4	4
4	5	2	3	4
6	6	6	6	6
6	6	5	5	5
5	4	6	4	4
4	5	5	4	5
6	6	6	6	6
4	4	4	4	4
6	5	5	4	6
6	6	6	6	6
7	7	7	7	7
5	5	5	6	5
5	5	3	4	5
6	6	5	5	5
5	6	6	5	4
6	6	5	5	5
4	3	3	4	3
4	4	5	5	5
6	6	6	6	7
5	4	4	4	5
5	4	7	7	5
6	6	6	5	5
4	4	4	3	2
5	6	5	6	6
6	5	4	6	4
7	7	6	7	6
5	5	5	5	6
4	4	4	4	4
6	6	6	6	6
6	3	4	6	7
5	5	5	5	5
6	6	6	6	6
6	6	6	6	6
5	5	5	5	5
5	5	5	5	5
7	7	6	6	6
6	6	6	7	7
4	4	4	4	4
6	6	5	5	6
4	5	6	5	6
7	7	7	7	6
6	6	6	6	6
7	7	6	5	6
5	5	4	5	5
7	7	6	6	6
4	5	5	5	5

5	5	3	3	3
6	6	6	5	6
6	6	5	5	5
7	6	6	6	7
5	6	6	6	5
5	5	5	5	6
6	6	5	5	4
5	5	5	5	5
6	6	5	5	5
6	6	6	6	6
5	4	6	6	6
5	5	5	5	6
5	5	5	5	5
6	6	6	6	6
7	5	6	6	6
5	5	5	5	6
5	5	6	6	6
4	4	4	4	5
6	6	4	4	5
5	6	4	5	5
7	7	6	7	6
6	6	7	6	6
6	6	5	6	6
7	7	7	7	6
6	6	6	6	6
4	6	6	6	6
4	5	5	4	4
6	6	6	5	5
6	6	6	6	5
7	7	7	7	7
6	6	6	6	6
6	6	6	6	6
6	6	5	5	5
5	4	5	5	5
6	5	6	5	5
6	6	6	6	6
7	7	7	6	7
7	7	7	7	7
4	4	5	4	6
7	7	7	7	7
7	6	6	6	7
7	7	7	7	7
5	5	5	6	5
5	5	6	6	6
6	7	7	7	7
5	4	7	5	5
7	7	7	6	7
5	5	5	5	5
6	6	6	6	6
6	5	5	5	5
5	6	6	5	5
4	4	4	4	4
5	5	5	5	5
6	6	6	6	6
6	6	6	6	6
6	6	5	5	7

5	5	5	5	6
5	6	6	6	5
5	6	6	6	6
5	5	4	4	6
7	7	6	6	6
6	6	4	4	4
7	7	7	7	7
7	7	6	6	7
5	5	5	5	5
5	6	5	5	6
5	5	5	4	5
5	7	5	6	6
6	6	5	5	5
4	5	5	4	5
6	7	6	7	6
7	7	6	6	6
5	4	6	4	5
5	5	4	5	6
7	7	6	6	7
6	6	6	6	6
7	7	4	4	5
7	7	6	6	7
7	7	7	7	6
5	5	5	6	6
6	6	6	6	6
6	6	5	5	6
7	7	6	7	7
7	7	5	6	7
7	7	7	7	7
7	6	7	7	6
6	6	6	6	4
6	6	7	7	7
6	6	7	6	6
6	6	6	6	6
6	6	5	4	4
7	7	5	5	6
6	6	7	6	6
7	6	6	6	7
3	3	4	5	3
5	5	6	5	3
6	5	5	6	6
6	6	6	6	6
7	7	7	7	7
5	5	4	4	5
6	6	5	5	6
6	6	5	5	6
6	6	6	6	6
6	7	7	6	6
4	4	4	4	4
4	5	5	5	7
7	7	7	7	7
7	7	7	7	7
6	6	6	6	6
6	6	5	5	6
7	7	6	6	6
5	6	6	6	6

5	6	7	7	7
5	6	6	5	5
5	6	5	5	5
5	5	5	5	5
5	5	4	4	4
6	6	6	5	6
7	7	7	7	7
5	5	6	5	6
6	6	6	6	7
5	5	5	5	5
5	4	4	5	4
7	6	6	6	7
6	6	6	6	6
4	4	3	5	5
5	5	5	6	6
5	5	5	5	5
6	6	6	5	6
3	4	7	4	3
6	6	6	6	6
6	5	6	5	6
5	5	5	5	5
4	5	4	6	4
5	5	4	4	4
6	5	5	5	5
4	4	4	4	4
6	7	7	6	7
2	3	7	7	7
4	4	5	5	4
7	6	5	6	6
6	6	6	6	6
4	5	6	5	5
7	7	6	7	6
6	6	4	4	4
6	6	6	5	6
6	5	5	6	5
5	6	5	6	6
3	1	4	4	4
6	6	5	5	6
5	5	6	5	6
5	5	4	4	6
6	5	5	6	5
7	7	7	7	7
7	7	7	7	7
6	6	7	7	6
7	7	6	7	6
6	6	6	6	6
4	4	4	4	4
4	4	3	3	4
5	7	6	5	7
6	5	5	5	6
5	6	6	6	6
5	5	5	5	5
7	7	6	6	7
5	5	5	5	5
6	5	5	5	5
4	4	3	3	4

4	4	4	4	4
6	6	5	6	6
6	6	6	6	6
6	5	6	5	7
7	7	6	7	7
6	6	2	2	2
6	4	5	5	7
7	7	5	5	6
6	6	5	5	7
7	7	7	7	7
4	4	4	4	4
7	7	5	7	7
5	3	6	5	5
6	6	5	4	6
5	6	6	5	6
3	5	5	3	5
5	6	4	4	5
6	6	6	6	5
7	7	7	7	7
6	4	6	4	6
6	6	6	6	6
6	6	6	7	7
6	6	6	6	6
3	4	5	4	3
7	6	4	5	6
6	6	5	5	6
7	6	5	5	6
6	6	7	6	6
5	6	5	6	5
5	6	6	6	6
6	6	6	7	7
7	7	6	6	6
5	5	5	5	5
7	7	5	6	6
6	5	6	6	7
6	6	5	5	6
7	7	6	7	6
7	7	6	6	7
5	6	6	5	5
6	6	6	6	6
6	6	5	5	6
4	4	5	5	3
7	7	6	6	7
6	6	6	6	6
6	6	6	5	6
6	6	6	6	6
7	7	5	5	6
7	7	7	6	6

15-4.Vegetation coverage	15-5.Overall coordination	16-1.Color richness	16-2.Visual effect	16-3.Cultural connotation
5	5	5	5	6
7	7	7	7	7
5	5	5	6	6
6	6	5	6	6
6	7	7	7	6
7	5	5	6	5
6	6	6	6	6
4	5	5	4	5
3	5	2	3	1
6	6	6	6	6
5	5	5	5	5
5	5	5	5	5
5	5	5	4	5
6	6	6	6	6
4	4	4	4	4
4	5	6	4	5
6	6	6	6	6
7	7	6	6	6
5	5	5	5	5
5	5	6	5	5
6	5	5	6	6
6	5	6	5	6
5	5	5	5	5
4	4	4	4	4
5	5	6	5	5
7	6	6	7	5
5	4	5	4	5
7	5	6	6	6
5	5	5	5	5
3	3	4	4	3
6	7	5	5	5
5	6	4	6	6
7	7	7	7	3
4	4	5	4	6
4	4	4	4	4
3	6	6	6	6
4	6	4	6	4
5	5	5	5	5
6	6	4	6	6
6	6	6	6	6
5	5	5	5	5
5	5	5	5	5
6	7	6	5	5
6	6	7	7	5
4	4	4	4	4
6	5	6	5	5
5	5	6	5	4
5	6	7	7	6
6	6	6	6	6
5	5	5	5	2
5	5	4	5	5
6	6	7	7	4
4	5	6	5	5

3	3	2	2	2
5	5	6	5	4
5	5	5	4	4
6	6	7	6	6
5	6	6	5	6
5	5	6	6	5
2	5	5	4	1
5	6	5	5	4
5	5	5	5	5
6	6	6	6	6
6	6	4	5	6
4	4	4	5	4
5	5	5	5	5
6	6	6	6	6
4	5	6	7	2
5	5	5	5	6
6	6	5	5	5
4	4	4	4	4
3	3	4	4	3
4	4	7	4	4
7	6	6	6	6
6	6	6	7	7
6	6	6	6	6
7	7	7	7	7
6	6	7	6	6
6	6	5	5	2
5	4	5	4	5
6	6	6	6	6
6	5	6	6	6
7	7	7	7	7
6	6	6	6	6
6	6	6	5	5
5	6	5	5	5
5	5	5	5	5
6	5	5	6	5
6	6	6	6	6
7	7	7	6	7
7	7	7	6	7
5	6	5	5	6
7	7	7	7	7
6	6	7	7	5
7	7	7	7	7
6	5	6	5	6
6	6	6	6	6
7	7	7	7	7
5	5	5	4	4
6	6	7	7	6
5	5	6	5	6
6	6	5	6	5
6	5	5	6	6
5	6	6	5	5
4	4	4	4	4
5	5	6	6	5
6	6	6	6	6
7	7	5	6	5
7	7	5	6	7

4	5	5	5	5
6	5	5	6	5
6	6	6	6	6
4	4	4	4	4
6	6	6	6	5
4	4	4	4	4
7	7	7	6	6
6	6	5	5	5
5	4	5	5	4
5	6	6	6	5
5	5	5	5	6
6	6	6	6	5
6	6	5	4	4
3	3	5	6	3
7	6	6	7	6
5	4	6	6	6
5	4	5	3	5
5	6	4	5	5
6	6	7	7	5
5	6	6	6	4
4	4	5	6	6
6	6	7	7	7
7	6	7	7	7
5	5	5	5	5
6	6	6	6	6
5	6	5	6	5
7	6	7	7	6
6	7	7	6	7
6	7	7	7	7
7	7	7	6	6
6	5	5	6	6
6	6	7	7	7
6	6	7	6	6
6	6	5	5	4
5	5	5	5	4
5	5	6	7	5
6	6	7	6	6
7	6	4	6	6
3	4	4	3	4
5	5	6	5	4
6	5	5	6	6
6	6	6	6	6
7	7	6	6	6
5	4	5	4	3
6	6	6	6	4
5	5	5	6	5
6	6	6	6	6
6	6	7	6	6
4	4	4	4	4
6	6	7	5	7
7	6	6	6	6
7	7	7	7	7
6	6	6	6	6
6	6	6	6	5
6	6	7	7	7
6	6	6	6	6

7	7	5	5	5
6	6	6	5	6
5	5	5	5	5
5	5	5	5	5
4	5	4	4	4
6	5	6	6	6
7	7	7	7	7
6	5	6	5	6
6	6	6	6	6
5	5	5	5	5
5	4	4	5	4
5	6	6	5	6
6	6	6	6	6
6	5	5	4	5
5	5	5	5	4
5	4	5	5	5
6	6	6	7	6
6	4	6	4	4
7	7	6	5	6
6	5	6	5	6
5	5	5	5	5
5	4	4	5	4
4	4	5	5	4
5	5	5	5	5
4	4	4	4	4
7	7	6	5	5
7	7	7	7	7
5	5	4	4	4
4	6	6	5	6
6	6	6	6	6
6	5	6	5	5
5	7	5	6	5
4	4	4	4	4
6	6	6	5	7
6	6	6	5	6
6	5	5	5	2
2	1	3	3	1
4	4	5	5	4
5	6	6	6	5
4	4	4	5	6
6	5	7	5	6
7	7	7	7	7
7	7	7	7	7
7	6	7	7	7
7	7	6	6	5
6	6	6	6	5
4	4	4	4	4
3	3	5	4	5
5	6	5	4	1
5	5	6	6	5
6	6	7	6	5
5	5	5	5	5
7	7	5	5	5
5	5	5	5	5
5	3	5	3	3
4	4	3	4	3

4	4	4	4	4
6	6	6	6	6
6	6	6	6	6
6	5	5	5	6
7	6	6	5	5
6	2	2	2	2
4	4	6	5	7
5	4	6	6	5
5	5	5	6	4
6	6	7	7	7
4	4	4	4	4
7	6	5	5	3
4	4	5	5	6
5	6	5	5	6
6	6	6	6	6
4	4	5	3	5
4	4	4	4	5
6	6	6	6	6
7	7	7	7	7
4	7	7	4	7
6	6	6	6	6
6	7	6	6	5
6	6	6	6	6
5	5	3	4	5
5	6	6	5	3
5	6	5	4	4
4	5	5	5	4
6	6	7	6	6
6	6	6	6	4
6	5	6	5	3
6	6	6	6	6
6	6	6	6	5
5	5	5	5	1
5	6	5	5	5
5	6	7	6	7
5	5	5	5	5
5	7	5	5	5
7	7	6	7	7
5	5	5	5	4
5	6	5	6	5
5	5	4	4	3
5	5	5	4	3
6	6	6	6	5
6	6	6	6	4
6	6	6	5	5
6	6	6	6	6
6	7	6	6	6
6	6	7	5	5

16-4.Vegetation coverage	16-5.Overall coordination	17-1.Color richness	17-2.Visual effect	17-3.Cultural connotation
5	5	5	5	6
7	7	7	7	7
5	5	5	6	5
6	6	6	5	6
7	6	6	6	7
7	6	6	5	7
6	6	6	6	6
5	5	5	5	5
1	2	2	5	5
6	6	6	6	6
5	5	5	5	5
5	5	6	6	4
4	4	4	4	6
6	6	6	6	6
4	4	4	4	4
4	6	6	5	5
6	6	6	6	6
6	6	6	5	6
5	5	5	5	5
6	5	4	5	6
5	6	5	6	5
5	6	6	6	5
5	5	5	5	5
4	4	4	4	4
5	5	5	5	5
6	6	7	6	7
4	6	5	4	5
6	6	6	4	5
6	6	6	6	5
3	4	5	4	4
4	4	5	5	6
5	4	4	6	4
6	7	6	4	6
4	6	4	5	6
4	4	4	4	4
3	6	6	6	6
5	4	4	6	4
5	5	5	5	5
6	5	4	4	5
6	6	6	6	6
5	5	5	5	5
5	5	6	6	6
6	6	6	6	7
6	6	6	6	6
4	4	4	5	4
6	6	5	5	6
6	5	6	5	5
6	7	6	7	7
6	6	6	6	6
4	5	5	5	6
4	5	4	5	4
7	7	6	6	6
4	5	5	5	5

2	2	4	4	4
4	4	6	5	5
4	5	5	4	5
6	6	6	6	6
5	6	5	6	6
7	7	6	5	5
5	5	5	5	7
4	4	5	5	6
5	5	6	5	6
6	6	6	6	6
4	5	5	5	6
4	4	5	4	6
5	5	5	5	5
6	6	6	6	6
5	5	7	7	7
5	5	5	6	7
5	5	5	6	5
4	4	4	4	4
3	3	5	5	5
5	5	5	4	6
6	6	6	5	6
6	6	6	7	6
6	6	6	6	6
7	7	7	6	6
6	6	6	6	6
6	6	5	5	5
4	4	5	4	5
6	6	6	6	6
5	5	6	6	5
7	7	7	7	7
6	6	6	6	6
6	6	6	6	6
5	6	5	5	5
5	5	5	5	5
6	5	5	5	5
6	6	6	6	6
6	7	6	7	6
6	7	7	7	7
6	4	5	5	6
7	7	7	7	7
6	7	6	6	7
7	7	7	7	7
5	6	6	6	5
6	6	6	6	6
6	7	7	7	6
4	4	4	4	5
7	7	7	6	7
5	6	5	5	7
6	6	5	5	6
6	5	5	5	6
6	5	5	5	5
4	4	4	4	4
5	5	5	5	6
6	6	6	6	6
7	7	5	5	5
5	5	5	6	7

5	5	5	5	6
5	6	5	6	5
6	6	6	6	6
4	4	4	4	6
5	6	6	7	7
4	4	4	4	4
6	5	7	7	7
5	5	7	6	7
5	5	5	5	4
4	4	6	5	6
5	5	6	5	5
6	5	5	5	6
5	5	5	5	5
5	5	5	4	6
7	6	7	6	7
6	5	5	4	5
5	4	5	3	5
4	5	4	4	4
6	6	6	6	7
6	6	6	6	6
6	6	4	4	4
7	7	7	7	7
6	6	6	6	7
5	5	5	5	6
6	6	6	5	6
5	6	6	5	7
7	7	7	6	7
6	7	6	5	6
7	7	7	6	6
6	6	7	6	7
5	5	5	5	6
7	6	7	7	7
6	6	7	7	7
4	4	6	6	6
5	4	5	5	5
4	5	3	4	6
6	6	7	6	7
5	6	7	6	6
5	4	4	3	5
3	4	6	3	6
6	5	6	5	6
6	6	6	6	6
7	7	7	7	7
5	4	5	4	5
6	6	6	6	6
5	6	5	6	6
6	6	6	6	6
6	6	6	6	6
4	4	4	4	4
6	5	6	6	6
6	6	6	6	6
7	7	7	7	7
6	6	6	6	6
6	6	6	5	6
7	7	7	7	7
6	6	6	6	6

5	5	6	6	6
5	6	6	5	6
5	3	5	5	5
5	5	5	5	5
4	4	4	4	4
6	6	6	6	6
7	7	7	7	7
6	6	6	5	6
6	6	6	6	7
5	5	5	5	5
4	5	4	5	4
6	5	6	5	7
6	6	6	6	6
5	5	4	5	5
5	5	5	5	6
4	4	5	5	5
6	6	6	7	6
4	5	6	4	5
6	7	7	7	7
6	5	6	6	6
5	5	5	5	5
5	4	3	5	3
5	5	4	4	4
5	5	6	6	5
4	4	4	4	4
5	6	6	5	5
7	7	7	7	7
4	4	4	4	4
6	6	5	5	6
6	6	6	6	6
5	6	6	5	5
6	6	6	5	6
4	4	4	4	4
6	6	5	6	5
6	5	5	6	5
5	5	4	4	6
2	2	4	4	6
6	6	5	5	6
6	6	5	5	6
4	5	4	5	6
6	4	5	6	5
6	7	6	6	6
7	7	6	6	6
6	6	7	7	7
6	7	6	7	6
5	6	6	5	6
4	4	4	4	4
4	4	4	4	5
4	3	5	4	7
5	5	6	6	7
6	6	6	5	6
5	5	5	5	5
5	6	7	7	7
5	5	7	7	7
6	6	5	3	4
4	4	3	3	3

4	4	4	4	7
6	6	6	4	5
6	6	6	6	6
6	6	6	5	6
6	6	7	7	7
6	2	2	2	5
4	4	5	5	7
5	5	5	5	7
5	5	5	5	7
6	6	7	7	7
4	4	4	4	4
6	5	7	6	5
4	4	6	5	6
6	6	5	4	5
6	7	5	6	6
4	4	5	3	5
4	4	5	5	5
6	6	6	5	7
7	7	7	7	7
4	6	6	4	6
6	6	6	6	6
6	6	6	7	7
6	6	6	6	6
4	4	5	4	4
6	5	5	5	7
5	5	5	6	6
4	4	6	5	7
6	6	6	6	6
5	6	6	5	6
4	4	3	5	4
6	5	6	6	7
6	5	7	6	7
1	1	5	5	5
5	5	5	5	7
6	6	6	6	7
5	5	6	5	6
6	4	6	5	7
7	7	6	7	7
6	5	6	5	7
6	6	6	6	6
4	3	5	5	6
5	5	5	5	5
5	5	6	7	7
6	6	6	6	6
6	6	6	6	5
5	5	6	6	7
6	6	6	7	6
7	7	7	7	7

17-4.Vegetation coverage	17-5.Overall coordination	18-1.Color richness	18-2.Visual effect	18-3.Cultural connotation
5	5	5	5	6
7	7	7	6	6
5	5	5	6	5
6	6	5	6	6
7	6	6	7	6
5	7	5	6	5
6	6	4	4	4
6	5	6	5	5
3	5	4	6	3
6	6	7	7	6
5	5	5	5	5
5	6	6	6	4
4	4	5	4	5
6	6	6	6	6
4	4	4	4	4
6	5	6	5	6
6	6	6	6	6
6	6	6	6	5
5	5	5	5	5
5	5	6	5	5
6	6	5	5	6
6	5	6	5	6
5	5	5	5	5
4	4	4	4	5
5	5	5	5	5
5	6	6	7	6
4	6	6	4	5
6	5	6	6	6
5	5	7	7	7
5	5	4	4	4
4	4	4	5	5
5	5	6	4	4
5	5	6	6	6
4	5	5	5	7
4	4	5	4	6
3	6	6	6	6
3	5	4	5	5
5	5	5	5	5
5	5	4	6	6
6	6	6	6	6
5	5	5	5	5
6	6	6	5	5
6	7	6	6	7
6	6	7	7	6
4	4	4	4	4
5	6	5	5	5
5	4	6	4	5
6	7	6	7	5
6	6	6	6	6
6	5	5	6	2
5	5	4	5	4
6	6	6	6	6
4	5	5	5	4

4	4	4	6	6
4	4	6	6	5
5	5	6	6	6
6	6	6	6	6
6	6	6	5	6
6	6	6	5	4
5	6	6	7	5
4	4	5	5	4
5	5	5	5	4
6	6	6	6	6
5	6	6	6	4
4	4	5	5	3
5	5	5	6	4
6	6	6	7	6
5	5	6	6	7
5	5	6	6	7
5	5	5	5	5
4	4	4	4	5
5	5	3	3	4
6	5	6	7	6
6	6	7	7	7
7	6	6	6	7
6	6	6	6	6
7	7	7	7	7
6	6	6	7	5
3	5	5	5	4
4	4	5	5	5
6	6	6	6	6
6	7	6	5	6
7	7	7	7	7
6	6	6	6	6
6	6	6	6	6
6	5	5	5	6
5	5	5	5	5
5	5	6	6	5
6	6	6	6	6
7	6	6	7	5
6	6	7	6	7
6	4	5	4	6
7	7	7	6	7
5	6	7	7	5
7	7	7	7	7
6	6	6	5	6
5	5	7	7	7
6	7	7	6	7
4	4	3	3	3
6	7	7	7	7
5	5	4	6	6
6	6	6	6	6
5	5	5	5	5
6	6	5	6	6
4	4	4	4	4
5	5	7	7	6
6	6	6	6	6
5	5	5	6	6
5	5	5	6	7

4	5	5	5	6
6	5	5	5	6
6	5	6	6	6
4	4	5	5	5
6	6	5	5	4
4	4	4	5	4
7	7	7	7	4
6	6	7	7	7
4	4	4	5	4
6	5	5	5	5
5	5	5	5	5
5	6	5	6	5
5	5	6	6	4
5	4	3	4	4
6	6	7	6	6
4	4	5	6	6
5	4	6	5	6
4	4	5	4	5
6	6	6	7	6
5	6	5	6	6
4	4	4	4	4
7	7	7	7	7
7	7	7	7	7
5	5	5	6	5
5	5	5	5	5
6	6	5	6	7
6	6	6	7	6
6	5	6	7	6
7	7	6	7	7
7	6	7	7	7
6	5	5	5	4
7	6	7	7	7
6	6	6	6	5
5	5	5	5	5
4	5	5	5	5
4	3	7	7	5
6	6	7	6	6
6	7	7	6	7
4	3	4	3	4
4	4	6	3	6
6	5	6	6	6
6	6	6	6	6
7	7	7	7	7
3	4	3	3	4
6	6	6	6	6
5	6	5	6	4
6	6	6	6	6
6	6	6	6	6
4	4	4	4	4
7	6	6	6	7
6	6	7	7	7
7	7	7	7	7
6	6	6	6	6
6	6	6	6	6
7	7	7	7	7
5	6	6	6	6

6	6	6	6	6
5	6	6	5	5
5	5	5	5	4
5	5	5	5	5
4	4	6	6	6
6	5	6	6	6
7	7	7	7	7
6	6	6	5	6
6	6	6	6	6
5	5	5	5	5
5	4	5	4	5
6	5	6	6	5
6	5	6	7	7
5	5	5	6	5
5	5	5	5	5
5	5	5	5	6
7	5	7	6	5
5	4	6	5	6
6	6	7	7	6
5	5	6	5	6
5	5	5	5	5
5	6	6	4	6
4	4	5	5	5
5	6	5	5	5
4	4	4	4	4
6	6	6	6	6
7	7	5	4	5
4	4	6	6	6
6	6	6	5	5
6	6	6	6	6
6	5	5	5	6
6	6	5	5	6
4	4	4	4	4
6	6	6	5	6
6	5	6	6	7
5	5	6	4	1
3	3	4	4	4
6	6	5	5	6
5	5	6	5	5
4	5	4	5	6
5	6	6	5	5
6	6	6	6	6
6	6	7	6	6
7	7	7	6	6
7	7	7	6	7
6	5	5	5	5
4	4	4	4	4
4	4	3	2	5
5	5	5	5	6
5	5	5	6	6
6	6	6	5	6
5	5	5	5	5
7	7	7	7	6
6	6	7	7	6
6	3	5	5	3
4	4	3	3	4

4	4	4	4	5
4	4	5	5	6
6	6	6	6	6
5	7	7	6	6
7	7	7	7	7
5	2	2	2	2
4	5	5	5	7
4	4	6	4	5
5	5	6	5	5
6	6	7	7	7
4	4	4	4	4
5	5	4	7	5
4	4	5	5	5
5	5	5	6	6
6	6	6	5	6
3	4	5	4	5
4	4	4	4	5
6	6	6	6	4
7	7	7	7	7
7	7	7	7	7
6	6	6	6	6
6	6	6	7	5
6	6	6	6	6
4	4	4	5	4
4	5	5	4	5
6	5	3	4	4
5	5	5	6	6
6	6	6	6	6
5	5	6	6	6
5	3	4	5	4
6	6	6	7	6
7	7	6	6	6
5	5	4	4	4
5	5	5	5	4
5	5	7	7	7
5	5	5	5	5
5	6	6	7	7
7	7	7	7	7
5	6	5	6	4
5	6	6	5	5
5	5	5	7	4
5	5	5	4	5
5	6	6	7	7
6	6	6	6	3
6	5	5	6	5
6	6	5	4	5
6	7	5	5	6
7	7	7	7	7

18-4.Vegetation coverage	18-5.Overall coordination	19-1.Color richness	19-2.Visual effect
5	5	5	5
6	7	7	7
6	5	5	6
6	6	6	6
7	6	7	7
6	7	6	5
4	5	6	6
5	5	5	6
6	6	2	4
6	6	7	7
5	5	5	6
5	5	5	5
4	4	5	4
6	6	6	6
4	4	5	5
4	6	5	5
6	6	6	6
6	6	6	6
5	5	5	5
5	5	5	6
6	5	6	6
5	5	5	6
5	5	5	5
4	4	5	5
5	5	5	5
6	6	6	6
4	6	6	4
6	6	5	5
7	7	7	7
3	3	5	5
5	5	4	4
4	4	4	4
4	4	6	6
4	5	4	5
3	6	5	4
3	6	5	5
3	6	4	6
5	5	5	5
6	5	5	6
6	6	6	6
5	5	5	5
5	6	5	5
6	6	6	7
6	6	6	6
4	4	5	4
5	5	5	5
6	5	6	5
5	5	6	5
6	6	6	6
4	5	5	5
5	5	4	5
6	6	6	6
5	5	6	6

4	4	6	6
5	6	6	6
6	6	6	6
6	6	6	6
6	6	6	5
5	4	6	3
4	6	6	6
5	5	6	5
5	5	5	5
6	6	6	6
6	6	4	4
4	4	5	5
5	5	5	5
6	6	6	6
5	4	6	5
6	6	6	5
5	5	5	5
4	4	4	4
3	3	5	5
6	5	7	7
7	7	6	6
6	6	7	6
6	6	6	6
6	7	7	7
6	6	7	6
4	4	6	6
5	5	5	4
6	6	6	6
6	6	6	7
7	7	5	5
6	6	6	6
6	6	6	6
6	6	5	5
5	5	4	5
5	6	5	6
6	6	6	6
6	7	7	6
6	7	7	6
4	5	5	5
7	7	7	7
7	7	6	6
7	7	7	7
5	5	5	5
7	7	7	7
6	6	7	7
3	3	4	4
7	7	7	6
6	7	6	6
6	6	7	6
5	6	5	5
6	5	5	6
4	4	4	4
6	7	5	5
6	6	6	6
6	6	6	7
5	5	5	6

6	5	5	5
5	5	5	6
5	5	6	6
5	5	4	4
6	6	6	6
5	4	4	4
7	5	7	7
6	6	7	6
5	5	5	4
4	4	5	4
5	4	5	5
5	6	5	6
4	5	6	6
3	2	5	5
7	6	7	6
5	5	5	5
6	5	5	3
4	5	5	4
6	6	6	6
5	4	6	5
4	4	4	5
7	7	7	7
7	7	7	7
5	5	5	5
5	5	5	5
6	6	6	5
7	7	7	7
6	7	5	5
6	7	7	5
5	7	7	6
7	5	5	5
6	6	7	7
5	5	7	6
6	6	6	5
5	5	5	5
6	5	4	5
6	6	7	6
7	6	6	7
5	3	4	3
4	4	3	4
6	6	6	6
6	6	6	6
7	7	6	6
3	3	5	5
6	6	6	6
5	6	6	6
6	6	6	6
7	7	7	7
4	4	4	4
7	6	6	5
7	7	7	7
7	7	7	7
6	6	6	6
6	6	5	5
7	7	7	7
6	6	6	5

5	6	6	6
6	6	6	5
3	3	5	5
5	5	5	5
5	5	4	4
6	6	6	6
7	7	7	7
5	5	6	5
6	6	6	6
5	5	5	5
4	5	4	5
5	6	6	6
6	6	6	6
5	5	7	6
5	5	5	5
5	4	6	7
7	6	7	6
5	6	6	5
6	6	7	7
6	5	6	5
5	5	5	5
4	3	4	5
5	5	5	4
5	5	5	5
4	4	4	4
6	6	5	6
4	5	4	5
6	6	6	6
4	6	5	5
6	6	6	6
5	5	6	5
5	4	3	4
4	4	4	4
6	6	6	5
5	6	6	6
5	5	5	5
3	3	4	4
6	6	5	4
6	6	5	5
4	5	4	5
5	6	7	5
6	6	6	6
6	6	6	6
6	6	7	6
6	6	6	5
5	5	6	6
4	4	4	4
3	3	3	2
5	7	7	6
6	6	5	5
5	5	5	6
5	5	5	5
6	5	7	7
7	6	6	6
5	4	4	5
4	5	4	4

4	4	4	4
6	6	6	4
6	6	6	6
6	6	6	6
7	7	7	6
6	2	2	2
4	5	5	5
5	5	6	6
6	6	6	6
5	5	7	7
4	4	4	4
5	3	5	7
4	4	5	5
5	6	5	5
6	5	6	6
3	5	5	4
4	4	5	5
6	6	6	6
7	7	7	7
7	7	7	7
6	6	6	6
6	6	6	7
6	6	6	6
5	4	5	4
3	3	5	5
4	5	5	5
4	6	6	5
6	6	6	6
6	6	6	5
4	5	6	6
6	6	6	6
6	6	6	6
4	4	5	5
6	5	5	5
5	6	7	7
5	5	6	6
5	6	6	5
7	7	7	7
5	7	5	5
6	6	6	6
7	7	5	5
5	5	5	5
7	7	6	6
6	6	6	6
6	6	6	5
6	3	6	6
6	7	5	5
7	7	7	7

19-3.Cultural connotation	19-4.Vegetation coverage	19-5.Overall coordination	20-1.Color richness	20-2.Visual effect
6	5	5	5	5
7	7	7	7	7
5	6	5	5	5
6	6	6	6	6
6	7	7	6	7
7	5	7	7	4
6	6	6	5	5
6	5	5	5	5
3	5	3	3	5
7	5	6	7	7
5	5	5	6	5
5	5	5	5	6
5	4	4	5	4
6	6	6	6	6
5	5	5	5	5
6	6	5	4	4
6	6	6	6	6
6	6	5	7	7
5	5	6	5	5
7	5	6	5	5
5	6	6	5	5
6	5	5	6	5
5	5	5	5	5
5	5	5	5	5
5	5	5	5	5
6	6	6	6	6
6	5	5	6	4
5	5	5	5	6
7	7	7	6	6
5	5	5	5	5
4	5	5	5	5
4	4	5	6	4
7	6	6	4	4
4	6	4	5	5
6	7	5	3	7
6	3	6	6	6
4	6	4	5	4
5	5	5	5	5
6	5	6	5	6
6	6	6	6	6
5	5	5	5	5
6	6	6	6	6
7	7	7	7	7
5	6	6	6	7
4	4	4	4	4
6	6	6	6	5
4	5	5	6	5
7	5	6	6	6
6	6	6	6	6
5	6	5	5	5
4	5	4	4	5
6	6	6	6	5
5	5	5	5	5

6	6	6	7	7
6	5	5	6	6
5	5	5	6	6
7	6	6	6	6
6	6	6	5	6
5	3	4	4	5
6	5	7	6	5
5	5	6	5	5
5	5	5	6	6
6	6	6	6	6
6	5	6	5	6
6	3	3	5	5
5	5	5	5	5
6	6	6	6	6
7	5	5	6	5
6	6	6	5	5
5	5	5	5	5
4	4	4	5	4
5	5	5	7	6
7	6	5	7	7
7	6	6	6	6
6	7	6	6	6
6	6	6	6	6
7	7	7	7	7
6	6	6	6	6
6	6	6	5	5
4	5	4	5	4
6	6	6	6	6
6	5	7	7	6
5	5	5	5	5
6	6	6	6	6
6	6	5	6	6
5	7	6	6	6
3	5	4	6	5
6	5	5	6	5
6	6	6	6	6
6	6	7	7	5
6	7	7	6	6
5	6	4	6	5
7	7	7	7	7
7	6	6	7	7
7	7	7	7	7
6	5	5	6	5
7	7	7	7	7
6	7	7	7	7
4	4	4	5	6
7	6	7	7	7
4	5	6	6	6
6	6	6	6	6
5	6	6	5	5
6	6	6	5	6
4	4	4	4	4
6	5	5	5	5
6	6	6	6	6
7	5	5	7	7
7	5	5	5	6

6	5	5	5	5
5	5	5	5	6
6	6	6	6	6
7	4	4	4	4
6	6	6	5	6
5	4	4	5	4
7	6	6	7	7
7	6	6	7	7
5	5	5	5	5
5	5	6	5	6
6	5	5	6	5
6	6	5	4	5
6	6	6	7	7
6	5	5	5	5
6	6	7	7	6
6	5	4	6	5
6	5	4	6	6
5	5	5	5	4
6	6	6	6	6
6	6	5	6	6
5	4	4	5	5
7	7	7	7	7
7	7	7	6	6
6	5	5	5	5
5	5	5	6	6
7	5	6	6	6
7	7	7	6	6
5	5	5	6	6
7	6	5	7	7
7	6	6	5	7
6	5	6	5	5
7	6	6	7	7
6	6	6	7	6
6	5	5	6	6
5	5	5	4	4
5	5	4	6	6
7	6	6	7	7
7	6	7	7	4
4	5	4	4	5
6	5	3	6	5
6	6	6	6	6
6	6	6	6	6
7	7	7	7	7
5	5	4	5	5
6	5	6	6	6
6	6	6	6	6
6	6	6	6	6
7	7	7	7	7
4	4	4	4	4
7	7	6	7	6
7	7	7	7	7
7	7	7	7	7
6	6	6	6	7
5	5	5	5	5
7	7	7	7	7
6	6	5	6	5

5	6	6	7	7
6	5	6	6	5
5	4	4	6	6
5	5	5	5	5
4	4	4	4	4
6	6	6	6	6
7	7	7	7	7
5	5	6	6	6
6	6	6	6	6
5	5	5	5	5
4	5	4	4	5
7	7	6	6	6
6	7	7	7	6
6	7	6	3	3
6	5	5	6	6
6	5	6	6	6
6	6	5	6	7
5	4	5	6	5
7	7	7	6	6
6	5	6	6	5
5	5	5	5	5
4	5	5	6	5
5	5	5	5	4
5	5	5	5	5
4	4	4	4	4
6	6	6	6	6
4	5	4	4	5
6	6	6	5	5
6	6	5	6	6
6	6	6	6	6
6	6	6	6	5
5	4	4	4	5
4	4	4	4	4
6	6	6	6	6
5	6	7	5	6
6	6	5	6	5
5	3	4	5	5
6	6	6	6	5
6	5	5	5	5
6	4	5	4	5
6	5	6	6	5
6	6	6	7	6
6	6	7	7	6
6	6	6	7	7
7	7	7	7	6
6	6	6	6	7
4	4	4	4	4
2	3	3	4	3
5	6	6	7	5
6	5	5	6	6
5	5	5	5	6
5	5	4	5	5
7	7	7	7	7
6	6	6	6	6
4	5	6	5	4
5	5	4	4	4

7	4	4	4	4
5	6	4	5	5
6	6	6	6	6
6	6	6	7	6
7	7	7	7	7
6	5	2	2	2
7	4	4	4	5
5	5	4	5	5
7	6	6	5	5
7	5	5	7	7
4	4	4	4	4
7	6	6	7	6
6	4	4	6	5
6	6	6	6	5
5	6	6	5	6
5	5	3	5	5
5	5	5	5	5
7	6	6	6	6
7	7	7	7	7
7	7	7	7	7
6	6	6	6	6
7	6	6	7	7
6	6	6	6	6
4	4	4	4	4
5	5	5	6	6
5	4	5	5	5
7	5	5	6	6
6	6	6	6	6
6	6	6	6	6
6	5	5	5	5
7	6	5	6	6
6	6	6	6	6
5	5	5	4	4
6	5	6	6	6
7	6	7	6	6
6	6	6	6	6
7	5	6	6	7
7	7	7	7	7
6	5	5	5	5
6	5	6	6	6
6	4	4	6	5
5	5	5	5	5
7	6	7	6	7
6	6	6	6	6
6	6	6	6	5
6	6	6	6	6
6	6	6	5	5
7	6	7	7	7

20-3.Cultural connotation	20-4.Vegetation coverage	20-5.Overall coordination	Total points	Average value
6	5	5	123	1.23
7	7	7	287	2.87
5	6	6	121	1.21
6	6	6	177	1.77
6	7	7	260	2.6
6	7	5	221	2.21
5	5	5	154	1.54
6	6	4	51	0.51
3	5	3	2	0.02
7	7	7	208	2.08
6	6	6	115	1.15
6	4	5	93	0.93
5	4	4	40	0.4
6	6	6	209	2.09
5	5	5	45	0.45
4	4	4	129	1.29
6	6	6	222	2.22
7	7	7	234	2.34
5	6	6	138	1.38
6	6	6	71	0.71
5	6	5	150	1.5
6	5	5	127	1.27
5	5	5	99	0.99
5	5	5	-80	-0.8
5	5	5	87	0.87
7	6	6	219	2.19
5	4	6	63	0.63
6	5	6	154	1.54
6	6	6	173	1.73
5	5	5	31	0.31
5	5	5	109	1.09
4	5	4	73	0.73
6	4	6	148	1.48
4	4	6	106	1.06
6	4	3	72	0.72
6	5	6	132	1.32
6	4	4	51	0.51
5	5	5	103	1.03
6	5	6	74	0.74
6	6	6	183	1.83
5	5	5	89	0.89
6	5	5	141	1.41
7	6	7	231	2.31
7	6	6	214	2.14
4	4	4	21	0.21
6	5	5	126	1.26
5	6	5	127	1.27
6	6	6	220	2.2
6	6	6	185	1.85
4	6	5	137	1.37
4	5	4	66	0.66
5	5	5	171	1.71
5	4	5	90	0.9

7	7	7	71	0.71
6	5	6	127	1.27
6	5	6	113	1.13
7	6	6	219	2.19
6	6	6	164	1.64
3	5	5	92	0.92
5	2	4	92	0.92
5	4	4	111	1.11
6	6	6	112	1.12
6	6	6	201	2.01
4	5	6	123	1.23
5	3	4	64	0.64
5	5	5	86	0.86
6	6	6	206	2.06
7	6	5	172	1.72
7	5	5	148	1.48
5	5	5	111	1.11
4	4	4	-44	-0.44
5	6	6	76	0.76
7	7	7	149	1.49
7	7	6	207	2.07
7	6	6	227	2.27
6	6	6	204	2.04
6	7	7	266	2.66
6	6	6	193	1.93
4	5	6	114	1.14
4	5	5	56	0.56
6	6	6	190	1.9
7	7	5	187	1.87
5	5	5	245	2.45
6	6	6	202	2.02
6	6	6	210	2.1
5	6	5	110	1.1
4	5	4	55	0.55
5	5	5	132	1.32
6	6	6	205	2.05
7	5	7	261	2.61
7	7	7	283	2.83
4	5	4	87	0.87
7	7	7	297	2.97
7	6	6	242	2.42
7	7	7	299	2.99
6	5	6	153	1.53
6	6	6	193	1.93
7	7	6	278	2.78
5	5	5	38	0.38
7	7	7	261	2.61
5	7	6	174	1.74
6	6	6	194	1.94
5	6	5	123	1.23
6	6	5	156	1.56
4	4	4	-5	-0.05
6	5	5	133	1.33
6	6	6	190	1.9
7	6	6	199	1.99
7	5	5	190	1.9

6	5	5	92	0.92
5	5	6	140	1.4
6	6	6	191	1.91
6	4	4	50	0.5
6	6	6	194	1.94
4	4	4	24	0.24
7	6	6	243	2.43
7	6	7	225	2.25
4	4	4	59	0.59
5	4	5	125	1.25
5	5	5	96	0.96
6	6	6	177	1.77
6	6	6	155	1.55
6	5	4	47	0.47
6	7	6	224	2.24
6	5	5	96	0.96
5	6	5	94	0.94
4	5	4	49	0.49
7	6	6	212	2.12
6	5	5	134	1.34
5	1	5	170	1.7
7	7	7	251	2.51
6	6	7	246	2.46
6	5	5	121	1.21
6	5	5	141	1.41
7	6	6	189	1.89
6	7	7	262	2.62
7	6	7	159	1.59
6	6	5	255	2.55
6	6	7	237	2.37
6	6	6	138	1.38
7	6	6	257	2.57
6	6	6	198	1.98
6	6	6	145	1.45
4	5	4	92	0.92
6	6	7	108	1.08
7	6	6	240	2.4
7	7	7	229	2.29
3	4	5	-14	-0.14
5	5	5	65	0.65
6	6	6	174	1.74
6	6	6	196	1.96
7	7	7	282	2.82
5	5	4	48	0.48
6	6	6	176	1.76
6	6	6	155	1.55
6	6	6	205	2.05
6	6	6	231	2.31
4	4	4	29	0.29
6	6	6	153	1.53
7	7	7	275	2.75
7	7	6	291	2.91
6	6	6	230	2.3
5	5	5	173	1.73
7	7	7	280	2.8
6	6	5	179	1.79

6	7	6	161	1.61
6	5	6	167	1.67
6	5	5	104	1.04
5	5	5	105	1.05
4	4	4	66	0.66
6	6	5	166	1.66
7	7	7	223	2.23
5	6	6	161	1.61
6	6	6	195	1.95
5	5	5	132	1.32
4	5	4	55	0.55
5	7	5	167	1.67
6	7	6	167	1.67
5	6	5	104	1.04
6	5	5	144	1.44
5	6	6	113	1.13
5	6	5	196	1.96
5	4	6	106	1.06
7	7	6	232	2.32
6	6	5	187	1.87
5	5	5	84	0.84
4	5	4	81	0.81
5	5	5	68	0.68
5	5	5	128	1.28
4	4	4	45	0.45
7	6	6	201	2.01
4	5	4	54	0.54
5	5	5	59	0.59
7	7	7	118	1.18
6	6	6	196	1.96
6	6	6	149	1.49
6	6	5	179	1.79
4	4	4	52	0.52
6	5	6	179	1.79
5	5	7	193	1.93
5	6	5	130	1.3
6	3	4	28	0.28
6	5	4	128	1.28
6	5	5	135	1.35
6	4	5	82	0.82
5	6	5	126	1.26
7	6	6	170	1.7
7	7	6	214	2.14
7	7	7	257	2.57
7	6	7	233	2.33
6	6	6	166	1.66
4	4	4	-10	-0.1
5	3	3	-49	-0.49
5	6	6	140	1.4
6	5	5	172	1.72
5	6	5	151	1.51
5	5	5	79	0.79
7	7	7	277	2.77
6	5	6	126	1.26
4	5	6	95	0.95
4	4	3	-37	-0.37

7	4	4	63	0.63
5	5	5	119	1.19
6	6	6	173	1.73
6	6	6	195	1.95
7	7	7	280	2.8
5	5	2	-83	-0.83
7	5	5	112	1.12
6	5	5	150	1.5
7	5	5	160	1.6
7	7	7	273	2.73
4	4	4	-11	-0.11
7	7	7	174	1.74
6	4	4	84	0.84
6	6	6	72	0.72
6	5	5	172	1.72
3	5	3	40	0.4
5	5	5	22	0.22
7	6	6	187	1.87
7	7	7	279	2.79
7	7	7	167	1.67
6	6	6	198	1.98
7	6	6	225	2.25
6	6	6	215	2.15
4	4	4	17	0.17
7	5	6	130	1.3
5	5	5	145	1.45
6	5	4	150	1.5
6	6	6	201	2.01
6	6	6	165	1.65
6	5	4	128	1.28
7	6	5	207	2.07
7	7	7	248	2.48
4	4	4	29	0.29
7	5	6	160	1.6
7	5	6	203	2.03
6	6	6	145	1.45
6	5	5	200	2
7	7	7	271	2.71
6	5	4	136	1.36
6	6	6	174	1.74
5	5	5	122	1.22
5	5	5	66	0.66
7	6	7	203	2.03
6	6	5	176	1.76
6	6	5	172	1.72
6	6	6	167	1.67
6	6	6	203	2.03
7	7	7	229	2.29

Standard deviation	A1 standardization value	A2 standardization value	A3 standardization value
0.158429795	-0.189	1.073	1.073
0.202731349	0.641	-0.345	0.641
0.306431069	0.62	0.62	1.273
0.386134692	-0.44	0.596	-3.03
0.20976177	0	1.907	0.953
0.640234332	1.234	1.234	1.234
0.510294033	0.901	-1.058	-1.058
0.604896685	-2.496	-1.174	-1.174
0.885211839	-0.7	1.559	0.203
0.342928564	-0.233	-0.233	-0.233
0.536190265	1.585	-0.28	-0.28
0.324191302	-2.252	-1.018	-1.018
0.352136337	-0.568	-0.568	0.568
0.214242853	-0.42	2.38	-0.42
0.524880939	-0.095	-0.476	0.286
0.471062629	-0.616	1.083	0.658
0.619354503	-1.97	-1.001	-2.293
0.518073354	-1.042	-0.27	-1.428
0.394461658	1.572	1.572	1.572
0.66174013	1.345	-0.166	1.043
0.360555128	-0.277	0.832	1.387
0.364828727	-1.288	0.356	-2.933
0.417013189	0.024	0.024	-0.456
0.977752525	-0.818	0.818	0
0.58403767	1.935	-1.832	-0.462
0.232163735	0.043	0.043	-1.68
0.347994253	2.213	0.489	0.489
0.447660586	-1.206	0.134	-1.206
0.743706932	-1.519	-0.444	-0.175
0.6212085	-0.499	0.789	-0.177
0.534696175	0.206	-0.168	0.206
0.452879675	-1.17	-0.287	-0.287
1.070327053	0.673	1.42	-0.075
0.400499688	-0.649	-0.649	-0.15
1.208966501	1.224	1.39	-3.077
0.42142615	-1.708	-1.234	-0.759
0.511761663	-1.778	-0.606	-0.215
0.439431451	-0.979	-0.068	-1.434
1.014100587	-2.307	0.256	-2.307
0.386134692	0.44	-0.078	-2.15
0.204694895	0.537	-0.44	-2.394
0.462493243	-0.454	-0.022	-0.022
0.337490741	-0.919	-0.326	0.267
0.405462699	-0.839	0.148	1.628
0.337490741	2.341	2.933	-0.03
0.18	-1.444	-0.333	-0.333
0.58403767	-1.147	2.277	-2.175
0.404969135	-1.482	0	-0.988
0.550908341	-3.358	-0.454	-0.454
0.978314878	0.644	1.257	-1.4
0.237486842	-2.779	1.432	1.432
0.617980582	-0.825	-2.12	-0.178
0.204939015	0.488	0.488	1.464

1.513241554	-0.073	0.059	-0.469
0.452879675	-0.155	1.17	-0.596
0.522589705	0.134	0.134	0.517
0.204694895	0.049	-0.928	-0.928
0.149666295	-1.604	-0.267	1.069
0.851821578	-1.315	0.798	-0.376
0.752063827	-1.223	-0.16	0.106
0.337490741	1.452	0.859	0.267
0.470744092	-1.105	-0.255	-0.68
0.172916165	-0.058	3.412	-2.371
0.457274535	-0.503	0.809	1.247
0.349857114	1.601	2.172	0.457
0.276405499	0.507	1.23	0.507
0.323109888	2.909	1.052	-2.043
0.426145515	-1.22	0.188	-0.751
0.49959984	-2.562	-1.361	-1.761
0.471062629	-0.234	0.191	1.465
0.665131566	-1.143	-2.345	-0.842
0.728285658	0.879	0.055	-0.494
0.823346828	-1.81	-0.595	-0.352
0.430232495	-1.557	-0.163	-0.163
0.192613603	-0.363	1.713	1.713
0.241660919	-1.821	2.317	0.662
0.41521079	-3.035	-1.59	-1.59
0.212367606	-3.437	-0.612	-1.554
0.672606869	-1.695	0.387	1.279
0.233238076	1.029	0.171	1.029
0.387298335	0.258	0.258	-2.324
0.255147016	-2.626	-1.058	0.51
0.920597632	0.597	0.597	0.597
0.087177979	4.359	-0.229	-0.229
0.577927331	-1.903	-0.865	-0.173
0.5	-0.2	0.2	-0.2
0.428369	-1.284	-0.817	-0.817
0.312409987	-1.024	-1.024	-0.384
0.226936114	-0.22	-0.22	0.661
0.256709953	1.519	-0.039	-1.597
0.158429795	-0.189	-0.189	-0.189
0.347994253	2.098	1.523	0.374
0.071414284	0.42	0.42	0.42
0.309192497	0.582	-0.065	-0.065
0.043588989	0.229	0.229	0.229
0.262868789	0.266	1.027	1.788
0.457274535	-0.284	0.153	0.153
0.166132477	1.324	0.12	-1.083
0.606300256	-1.286	-0.297	-0.957
0.223383079	-0.94	-1.835	-0.045
0.518073354	-1.042	0.888	0.502
0.303973683	1.513	0.197	0.197
0.14525839	-0.207	-0.207	-0.207
0.174355958	-2.065	-0.918	0.229
0.34568772	2.459	-1.591	0.145
0.470212718	-0.276	1	0.574
0.240831892	0.415	-2.907	-0.415
0.365923489	0.027	0.027	1.12
0.495983871	-1.008	-1.008	0.202

0.285657137	0.98	-1.12	-1.12
0.282842712	0.707	-0.707	1.414
0.147986486	0.608	-2.095	0.608
0.264575131	0.378	-0.378	-1.134
0.410365691	-1.316	0.146	-0.829
0.631189354	-0.38	0.887	-0.38
0.370270172	-1.701	-0.621	0.459
0.579223618	-1.813	-0.086	0.95
0.349141805	-0.544	0.029	0.601
0.464219776	0.323	-0.108	0.323
0.32	1.375	1.375	-1.125
0.277668868	-0.612	-0.612	-1.333
0.596238207	-1.258	0.084	-0.922
0.639609256	-0.735	-0.422	0.516
0.412795349	-2.035	-2.035	-1.55
0.621610811	-0.579	-0.901	0.064
0.443170396	1.489	0.135	-0.316
0.608194048	-0.806	-2.45	-2.121
0.312409987	-1.024	-1.024	-0.384
0.790189851	0.329	1.088	-1.949
1.055461984	0.663	0.284	0.474
0.736138574	-2.051	-2.051	-2.051
0.469467784	-2.258	-0.98	-0.98
0.360416426	-2.802	0.527	1.082
0.3548239	-1.156	-0.028	-1.719
0.264386081	-1.097	1.929	0.416
0.218174242	-1.008	0.825	-1.008
0.786066155	-0.751	-0.242	-1.005
0.288963666	-1.211	0.865	0.173
0.401372645	0.573	-0.424	-2.417
0.351567917	-1.65	0.057	1.195
0.158429795	-2.335	-2.335	-1.073
0.45563143	-3.468	0.922	0.044
0.493457192	-0.912	0.304	-0.507
0.507543102	-1.419	0.552	1.34
0.713862732	-0.392	-0.112	-2.073
0.228035085	0.877	2.631	0.877
0.658710862	1.078	-2.566	0.167
0.58	-0.448	1.276	0.241
0.373496988	-0.134	-0.669	-2.276
0.603655531	-0.895	-0.563	-1.226
0.10198039	-3.53	0.392	0.392
0.198997487	-1.106	0.905	-0.101
0.479165942	-1.419	1.503	-1.002
0.28	-0.571	0.857	-2
0.315436206	-1.744	-0.476	-1.11
0.217944947	-0.229	4.359	-0.229
0.240624188	-1.288	1.205	0.374
0.574369219	2.629	-0.157	-0.157
1.284172886	-1.814	-0.88	-2.282
0.399374511	-1.878	-0.876	-1.878
0.204694895	-3.469	-1.514	0.44
0.42661458	-0.703	0.703	-0.234
0.284780617	0.948	0.948	-1.159
0.4	0.5	0.5	0.5
0.194679223	1.079	0.051	1.079

0.563826214	0.337	1.046	-0.372
0.192613603	1.713	0.675	1.713
0.527636238	-0.076	-0.076	1.061
0.23558438	-1.91	-0.212	2.335
0.657571289	0.517	2.038	0.213
0.395474399	-1.669	-1.669	-1.669
0.77272246	-0.298	-0.039	-0.039
0.204694895	-0.049	-2.98	0.928
0.198746069	-3.774	-0.755	0.252
0.64	0.125	-0.813	0.125
0.259807621	-1.347	0.962	-0.577
0.545068803	0.239	-0.495	-0.128
0.679043445	-0.692	1.37	0.486
0.889044431	-0.495	0.18	0.63
0.542586399	1.401	2.138	-0.442
0.386134692	-0.337	0.181	-0.337
0.52	-3.385	0.077	-0.692
0.797746827	-2.833	0.175	1.178
0.312409987	0.896	0.256	1.536
0.353694784	1.498	1.498	0.933
0.215406592	-1.114	-2.043	-0.186
0.453762052	-0.022	0.419	-0.022
0.49959984	1.041	0.641	-0.961
0.36	-2.444	-0.778	0.889
0.609507998	0.246	1.559	1.231
0.376696164	-0.557	-0.027	0.504
1.143853137	-0.822	-0.297	-1.171
0.773239937	0.53	-0.763	1.565
0.745385806	-1.851	-1.315	-1.046
0.224499443	0.178	-4.276	0.178
0.604896685	1.174	2.166	0.512
0.928385696	-1.713	-0.851	0.872
0.59126982	-1.556	0.474	-0.541
0.240624188	0.042	-3.283	-0.79
0.277668868	1.693	-0.468	0.972
0.608276253	-2.137	1.48	0.164
0.98264948	-1.099	0.733	0.936
0.453431362	0.706	-0.176	-1.5
0.327871926	0.762	0.152	-0.457
0.437721373	-1.416	-2.33	-0.96
0.31685959	-1.452	-0.821	-0.821
1.07424392	-1.583	-0.465	1.024
0.566038868	-0.954	-1.307	-0.601
0.277668868	0.108	-0.612	1.549
0.359304884	-2.032	0.195	-2.588
0.335261092	1.014	-0.776	-0.776
0.449444101	-0.667	0.222	0.667
0.545802162	-1.301	-0.934	-1.301
0.729383301	0	1.645	0.548
0.435430821	0.184	1.102	2.021
0.381968585	-0.812	-0.288	2.33
0.331511689	0.03	0.633	0.03
0.425558457	0.54	0.54	0.54
0.529528092	-0.491	-0.869	-0.491
0.493457192	-1.115	-1.52	-0.304
0.570175412	-0.403	-1.105	-1.105

0.499099189	-0.06	1.543	-0.06
0.614735716	-0.309	-0.309	1.643
0.664153597	-2.906	-2.605	0.407
0.267394839	-0.561	0.187	0.935
0.340587727	0.587	-0.587	-1.174
0.89280457	-0.414	0.258	0.482
0.263818119	-0.455	1.061	0.303
0.542217668	0.184	-0.553	-0.922
0.517687164	-1.159	-1.159	-0.773
0.291719043	-1.817	-1.131	0.24
0.407308237	-2.676	-0.221	-1.203
0.787654747	-1.955	-1.955	0.584
0.332264955	-0.722	-1.324	1.083
1.17541482	-2.314	-2.144	-1.804
0.256124969	1.093	-1.249	-2.03
0.57965507	0	1.38	0
0.625779514	-1.31	-0.032	-0.991
0.255147016	-1.842	0.51	0.51
0.39736633	0.025	0.528	-1.988
1.317990895	-1.115	-2.329	0.25
0.244131112	-1.557	0.901	0.901
0.243926218	-2.665	-1.025	-0.205
0.389230009	-1.927	-0.385	-0.385
0.212367606	-1.742	1.083	-0.8
0.893308457	-3.022	0.56	1.007
0.599583189	-0.751	0.917	-0.083
0.42661458	0.234	0.703	-0.234
0.271845544	-2.244	-1.508	-1.508
0.321714159	-1.399	0.466	1.088
0.611228272	-2.421	0.196	0.196
0.212367606	-2.213	0.612	-1.271
0.416653333	-1.632	0.288	-0.672
1.252956504	-1.349	-1.508	1.365
0.485798312	-1.235	1.235	-0.823
0.347994253	-0.086	-1.236	1.063
0.409267639	-1.588	-0.122	0.855
0.532916504	0.375	0	-0.751
0.256709953	-1.208	-2.766	-0.428
0.504380809	-0.714	1.269	1.269
0.228910463	-2.359	0.262	0.262
0.731846979	-0.301	0.519	-1.394
0.502394267	-1.712	0.677	-0.119
0.648922183	1.495	-0.663	-0.354
0.18547237	0.216	0.216	0.216
0.183303028	-2.837	-0.655	-0.655
0.63015871	0.206	-0.746	0.524
0.380919939	0.446	1.496	0.971
0.664755594	-2.241	0.165	0.165
SBE Value	-0.575	-0.025	-0.179

A4 standardization value	A5 standardization value	A6 standardization value	A7 standardization value
2.335	-0.189	-0.189	-0.189
-1.332	-0.345	-1.332	0.641
2.578	-1.991	-1.338	-0.685
0.078	0.078	0.596	1.114
-1.907	0	0	-0.953
1.234	1.234	1.234	1.234
-1.058	0.118	0.901	0.901
0.479	-0.182	-0.182	-0.182
1.107	0.429	0.203	0.429
2.1	-1.4	-1.4	0.35
1.212	-0.28	-2.145	0.466
-1.018	-0.401	-0.401	-1.018
3.408	-1.136	-0.568	-1.136
3.314	0.513	-0.42	0.513
2.572	1.048	0.286	1.048
1.507	0.658	-0.616	0.658
0.614	-0.678	0.936	1.259
1.274	-0.27	0.502	0.502
1.572	1.572	0.051	-0.456
-1.375	-1.98	0.438	0.136
1.387	0.832	-2.496	0.832
-1.288	-0.192	-0.192	0.356
2.902	-1.894	0.024	0.024
0.818	0	-1.227	-1.227
2.277	-1.49	0.565	-0.12
2.627	0.043	0.043	0.905
0.489	-0.661	-1.81	-0.086
1.474	0.581	-1.653	-0.313
0.363	0.363	0.094	-0.444
1.111	0.467	1.755	0.467
2.076	-0.542	-1.29	-0.916
0.155	-0.287	-0.729	-0.287
1.42	-1.383	-1.196	-1.009
0.849	-0.15	2.347	-0.15
1.39	-0.099	-0.43	1.555
1.139	0.664	-1.234	-1.234
2.912	-1.387	-0.215	-0.606
3.118	-0.523	-0.068	1.297
-0.73	-0.138	0.256	1.045
-0.078	1.476	-2.15	0.44
2.492	-0.44	-1.417	-0.44
1.708	3.005	-0.886	0.411
1.452	-0.919	-0.919	-0.326
-0.345	-0.345	0.641	-0.839
-0.622	1.156	-0.03	-0.03
-0.333	-0.333	-0.333	-1.444
1.935	-0.462	0.223	0.565
0	0.494	0	0.988
-0.091	-1.543	0.272	1.361
1.053	-0.174	-0.174	0.44
0.59	-0.253	-1.095	-0.253
0.469	-1.149	-1.149	-1.149
0.488	-0.488	-0.488	0.488

1.117	-1.13	-1.13	0.852
0.729	-0.155	0.729	0.287
2.047	-0.249	-0.631	-0.249
2.98	0.049	1.026	1.026
-0.267	-0.267	-0.267	1.069
1.268	-0.61	-2.254	-1.08
0.106	-1.489	0.904	-0.425
0.267	1.452	0.267	-0.919
1.02	-0.255	-0.255	-0.255
-0.058	1.099	-1.214	-0.058
-0.94	-1.815	0.372	0.809
1.601	-0.686	-1.258	0.457
-0.217	0.507	0.507	0.507
-0.186	-0.186	-0.186	1.052
1.126	0.188	-1.69	-0.282
0.641	0.24	-0.56	-0.16
0.616	1.04	-1.507	-1.932
-0.842	-0.842	-0.842	-0.842
-0.769	-0.494	0.604	-0.494
1.348	0.134	1.348	0.862
0.302	-0.163	-2.022	-0.628
-0.363	1.713	0.675	-0.363
2.317	-0.166	-0.166	1.49
-0.145	0.819	0.337	0.337
-0.612	0.33	0.33	0.33
1.279	0.684	-0.208	-0.803
1.029	-0.686	-0.686	1.029
2.324	-2.324	-1.291	0.258
0.51	0.51	0.51	0.51
0.597	0.597	0.597	0.597
-0.229	-0.229	-0.229	-0.229
1.557	-0.173	-0.173	-0.173
1.8	-1	-1.4	-0.6
-0.35	1.05	-0.35	-1.284
2.817	1.536	-1.024	0.896
4.186	-0.22	-0.22	-0.22
-0.818	1.519	1.519	-0.039
1.073	-0.189	1.073	1.073
1.523	-1.351	-1.351	-0.201
0.42	0.42	0.42	0.42
1.229	-1.358	-2.652	1.229
0.229	0.229	-4.359	0.229
1.027	1.788	-0.495	-0.495
-1.596	-1.596	0.153	0.153
0.12	-1.083	0.12	0.12
0.693	0.693	-0.297	0.033
0.851	-0.94	-0.045	-0.94
2.432	0.502	1.66	0.116
2.829	0.855	-0.461	-0.461
1.17	-1.583	-0.207	-1.583
-0.918	1.376	0.229	0.229
-3.327	0.145	0.145	0.145
2.701	-0.702	-0.702	-0.702
0.415	-2.907	0.415	0.415
0.027	0.027	-1.612	1.667
1.411	-0.605	2.218	1.411

-2.521	-1.12	-0.42	0.28
-1.414	0.707	0	-1.414
0.608	0.608	0.608	0.608
2.646	-0.378	-0.378	0.378
0.634	1.121	-1.316	-0.341
1.204	-1.965	0.887	0.57
-1.701	0.459	-0.621	-0.621
0.95	-1.813	-0.086	0.604
2.893	0.029	-1.117	0.029
1.185	-2.693	2.477	-0.108
-0.5	-0.5	-1.75	-1.125
0.828	1.549	0.108	1.549
2.096	-0.587	0.084	0.755
1.767	-1.673	1.454	-0.109
0.872	-1.066	-1.55	0.388
1.351	-1.223	-0.901	-0.579
1.489	0.587	1.038	-0.316
0.839	-1.135	1.167	-0.148
0.256	-1.664	-0.384	-0.384
-3.214	0.076	0.076	0.329
1.232	-0.663	1.232	0.284
0.666	0.666	0.666	-0.693
-0.98	-0.98	-0.98	-0.98
-1.692	0.527	2.192	1.082
1.099	-1.156	-0.028	0.535
1.173	0.416	-0.34	0.416
-0.092	-0.092	0.825	1.742
1.794	0.776	-0.242	-0.496
0.865	1.557	0.173	-0.519
0.573	0.075	-1.918	-0.922
1.764	-1.081	-1.081	-1.081
0.189	0.189	0.189	0.189
0.044	-0.395	-0.834	0.483
-2.128	-0.101	1.115	1.115
2.522	0.158	0.158	0.158
0.728	-0.672	-0.392	1.289
-0.877	0.877	0	-1.754
1.078	1.078	-1.351	0.167
3.345	0.241	0.241	-0.103
0.402	-0.669	1.473	-0.134
0.431	-0.563	-1.226	-1.226
-1.569	-1.569	0.392	0.392
0.905	-1.106	-0.101	0.905
1.92	-0.167	0.25	0.25
0.857	0.857	0.857	0.857
1.427	-1.11	0.159	0.793
-0.229	-0.229	-0.229	-0.229
-1.288	-0.457	-0.457	-0.457
2.629	1.236	-0.157	0.54
1.145	-1.191	0.366	-1.191
0.626	0.125	0.626	0.626
0.44	0.44	0.44	-1.514
1.641	1.641	-0.703	-0.703
0.948	0.948	0.246	0.246
0.5	0.5	0.5	0.5
1.079	1.079	-0.976	-0.976

-1.791	-0.372	-0.018	0.337
1.713	1.713	-1.402	0.675
1.819	-0.834	-0.455	-1.213
3.184	-0.212	-0.212	-0.212
2.038	0.213	-0.395	-0.395
0.354	-1.669	0.86	-0.152
0.479	-1.333	0.479	-0.298
0.928	-0.049	-0.049	-1.026
-0.755	0.252	0.252	0.252
2.313	1.375	2.625	0.75
3.272	1.732	-0.577	0.192
2.44	-1.596	-1.596	-0.495
0.486	-0.103	-0.987	-0.692
0.405	1.08	0.855	0.405
0.295	0.663	-0.074	0.295
-0.337	-1.891	-1.373	0.181
1.615	-0.308	-0.692	-0.692
1.68	-0.075	-1.078	-0.326
1.536	-1.024	-1.024	0.256
0.933	-0.198	0.368	0.368
-2.043	-0.186	0.743	-1.114
3.063	0.419	-0.022	-0.463
2.642	1.041	0.641	0.24
0.333	-0.222	0.889	2
1.559	1.559	-0.738	0.246
1.566	-1.619	-0.027	0.504
0.752	-0.647	-0.472	-0.647
-0.763	1.823	-0.763	-0.763
-0.241	-1.851	-1.315	0.295
0.178	0.178	0.178	0.178
1.835	0.182	-0.81	0.182
1.303	-0.851	-0.42	0.657
1.488	-0.203	-0.203	0.812
0.873	0.873	0.042	0.042
1.693	-0.468	-0.468	0.972
1.151	1.48	-0.164	1.151
1.954	0.122	1.343	1.14
2.47	-0.618	-1.059	-0.176
1.372	-0.457	0.762	0.152
1.325	-0.503	1.782	0.411
-0.189	0.442	-0.821	-0.821
-0.279	-0.465	-0.279	-0.093
0.106	-0.954	-0.954	-0.954
1.549	-0.612	1.549	-0.612
0.751	-0.918	0.195	-0.362
0.418	-1.372	-0.179	-0.179
0.667	2.447	-2.002	0.222
1.997	-0.568	-0.202	-0.202
0.548	-0.548	-0.548	0.274
2.48	0.643	0.184	-1.194
1.283	0.759	-1.859	-0.812
1.237	0.03	-0.573	1.84
0.54	0.54	0.54	0.54
-0.491	-0.491	-0.491	-0.491
0.507	-0.304	0.912	1.317
2.403	-1.105	-1.105	-2.157

1.543	-0.06	1.543	1.543
1.968	-0.309	-0.309	-0.96
0.407	-1.4	0.407	0.407
-1.309	0.935	0.935	1.683
0.587	0.587	0.587	0
2.274	0.482	-0.19	-0.414
2.578	-0.455	0.303	0.303
1.66	-0.553	0.922	0.922
1.159	1.159	1.932	0.773
0.24	0.926	0.926	0.926
2.725	0.27	-0.221	0.27
-0.178	0.33	0.33	-0.178
0.482	-1.324	2.287	-0.12
0.579	-0.272	-0.783	-0.442
-0.469	0.312	-0.469	0.312
3.45	0	-0.345	0.345
1.886	-0.032	-0.032	-1.31
-1.058	-0.274	-0.274	0.51
0.528	0.528	-1.988	-1.988
1.009	-1.115	1.009	1.009
3.359	-0.737	0.082	-1.557
0.615	0.615	0.615	-0.205
-0.385	0.128	2.184	2.184
2.025	0.141	-0.8	0.141
0.56	-0.112	0.784	1.231
-1.084	1.584	0.917	1.251
1.172	-0.703	0.703	-0.234
-0.037	-0.037	2.17	0.699
1.088	-2.02	0.466	1.088
1.178	0.196	0.196	0.524
1.554	-0.33	0.612	0.612
0.768	-0.672	1.248	0.768
2.163	-0.71	0.567	-0.87
1.647	0.823	-0.412	0.412
-0.086	-0.661	-0.661	0.489
-1.588	0.367	-1.1	-0.611
1.876	1.126	0.751	1.126
1.13	0.351	0.351	0.351
-0.317	-1.903	0.476	0.872
-0.612	-1.485	1.136	1.136
1.612	1.339	0.246	0.793
1.473	-0.916	0.677	0.677
0.262	-0.354	-0.971	-0.354
0.216	1.294	-0.863	0.216
-0.655	1.528	0.436	1.528
-2.333	0.524	0.524	0.524
0.971	0.446	0.446	0.971
-1.038	-0.737	0.767	-1.038
0.82	-0.114	-0.096	0.096

A8 standardization value	A9 standardization value	A10 standardization value	A11 standardization value
2.335	-0.189	-1.452	-1.452
0.641	-0.345	0.641	0.641
0.62	-0.033	-0.685	-0.685
-1.994	-1.476	0.596	0.596
0.953	-1.907	-0.953	0
0.297	-1.265	-0.64	-1.578
0.118	-0.274	-0.274	0.118
-0.843	0.149	0.81	0.81
0.655	0.429	-0.023	-0.023
-0.233	-0.233	-0.233	-0.233
-0.28	-1.772	-0.28	-0.653
0.216	0.216	0.216	2.067
-1.704	0	0	0
-0.42	-0.42	-0.42	-0.42
0.286	1.048	-1.238	-0.857
0.234	0.658	0.658	1.083
1.259	0.614	0.936	1.259
-0.656	-1.428	1.274	1.274
-0.456	0.558	0.051	-0.963
-0.468	-0.771	-0.771	-1.375
-0.277	0.277	-0.832	-0.832
0.905	1.453	0.905	0.356
0.024	0.024	0.024	-2.374
-0.205	-1.227	-1.227	-1.227
-0.805	0.223	-1.49	0.223
0.043	0.043	1.766	-1.68
-0.661	-1.236	-0.661	-1.236
-0.313	0.581	1.474	0.134
0.363	-2.595	1.708	0.094
0.467	-1.143	0.789	-0.177
0.206	1.702	0.58	-0.916
-0.729	1.921	1.479	-0.287
-1.756	-0.075	1.42	-0.075
-0.15	-1.149	1.848	0.35
0.397	0.232	0.232	-0.596
-0.759	-1.234	1.139	1.139
0.176	-0.215	-0.606	-0.606
1.297	-0.068	-0.068	-0.068
-0.335	-0.73	-0.73	-0.532
0.958	-1.632	0.44	0.44
0.537	-0.44	-0.44	-0.44
-0.022	-0.022	-0.886	-0.886
-0.919	-0.326	0.859	0.267
-1.825	-1.332	2.121	-0.345
-0.622	-0.622	0.563	-0.622
-0.333	-0.333	-0.333	-0.333
-1.147	-0.462	0.565	1.25
-0.988	-1.482	1.482	0
0.272	1.724	-0.454	0.272
0.44	-2.627	1.462	-0.174
0.59	0.59	-0.253	0.59
-1.149	0.793	0.146	0.469
-2.44	0.488	-0.488	-1.464

1.117	-0.734	1.381	-0.866
-0.155	-1.038	1.17	-0.596
-0.249	-0.631	-1.014	-0.249
-0.928	-1.905	0.049	0.049
-1.604	-0.267	-0.267	-1.604
-1.08	0.798	1.737	0.329
0.372	1.436	0.106	0.904
0.267	0.859	0.267	-0.919
-0.255	-1.954	-0.255	-0.255
-0.058	-0.058	-0.058	-0.058
0.372	-2.252	1.247	-0.066
-0.114	-0.114	0.457	-1.258
-0.941	-0.941	-0.941	-1.664
1.052	-0.805	-0.805	-0.186
0.188	-0.751	-0.282	0.188
1.041	0.641	0.641	-0.16
-0.658	-1.932	-0.234	-0.234
-0.842	-0.842	0.662	0.962
1.153	0.055	0.33	0.33
-0.838	0.377	-0.595	-1.324
0.302	0.767	-0.163	-0.628
-0.363	-1.402	-0.363	-1.402
-0.166	-0.993	-0.993	-0.166
-0.145	0.819	-0.626	0.819
0.33	-0.612	0.33	0.33
-1.398	-1.695	0.684	-0.208
1.886	-1.543	0.171	-1.543
0.258	0.775	0.258	0.258
0.51	0.51	-0.274	-0.274
0.597	-2.661	-1.575	-0.489
-0.229	-0.229	-0.229	-0.229
1.557	1.557	1.557	1.557
-1.4	0.2	-1.8	-0.6
-1.284	-1.284	-1.284	1.05
-0.384	0.896	-1.024	0.256
-1.102	-0.22	-0.22	-0.22
-0.039	-0.039	-0.039	-0.039
-0.189	-0.189	-0.189	1.073
-0.776	-0.776	-0.776	-0.776
-2.38	0.42	0.42	0.42
-0.065	0.582	-0.712	1.229
0.229	0.229	0.229	0.229
0.266	-2.016	0.266	-1.255
0.153	-0.284	-0.284	-0.284
1.324	1.324	-1.083	1.324
-0.297	2.342	0.363	-0.297
-0.94	-0.94	0.851	-0.94
0.502	0.116	0.116	-1.428
-0.461	-1.776	-0.461	-1.119
-1.583	1.17	1.17	-0.207
1.376	1.376	-0.918	0.229
0.145	0.145	0.145	0.145
-0.702	-0.702	-0.276	-0.276
0.415	0.415	0.415	0.415
0.574	-0.519	-0.519	-0.519
0.202	-1.008	0.202	0.202

0.28	-0.42	0.98	0.28
-1.414	2.121	-1.414	1.414
-0.743	0.608	0.608	0.608
-0.378	-0.378	0.378	-1.134
0.146	0.634	-0.829	0.634
-1.965	-0.38	-0.063	-0.063
-0.081	-0.621	-0.081	0.999
1.295	0.604	0.259	-0.432
-0.544	-2.263	-0.544	0.601
0.323	-0.108	0.754	0.323
-1.125	-1.125	1.375	1.375
-0.612	1.549	0.108	0.108
1.426	-0.922	-0.587	-0.587
-0.735	-1.36	0.203	-0.422
0.388	0.388	1.357	0.388
-0.901	0.708	-0.901	-0.901
0.587	0.587	-0.767	-1.218
-0.148	0.839	0.839	0.839
-0.384	2.177	0.256	-0.384
0.582	-0.683	0.835	0.835
-0.474	1.232	1.232	0.284
-0.693	-0.693	0.666	0.666
-0.128	1.15	1.15	1.15
-0.028	-0.583	-0.028	-0.028
-0.028	-1.156	0.535	-0.028
-0.34	-1.097	-0.34	-1.097
-0.092	-1.008	0.825	-1.008
-0.496	-1.514	0.013	-0.496
0.173	-1.903	0.173	-1.211
0.573	-0.922	0.075	-0.424
-1.081	1.764	0.057	0.057
0.189	-1.073	0.189	0.189
0.922	-0.395	0.483	-0.395
0.304	-1.317	1.115	0.304
0.552	-0.63	0.158	-0.63
-0.112	-0.953	0.728	-1.233
0.877	0	-0.877	0.877
-1.655	1.078	-0.744	-0.137
-1.828	-0.793	-0.448	-0.793
-1.205	1.473	0.402	-0.669
-1.226	1.756	0.431	-0.895
0.392	0.392	0.392	0.392
0.905	-1.106	-1.106	0.905
-0.167	-0.167	-0.584	0.25
0.857	-1.286	-1.286	-2
0.159	-0.476	1.427	-1.11
-0.229	-0.229	-0.229	-0.229
-0.457	-0.457	-0.457	-0.457
-0.505	-1.201	-0.505	-0.505
-0.724	0.989	1.145	1.145
0.626	0.626	0.626	0.626
0.44	0.44	0.44	0.44
1.641	1.641	1.641	-0.703
0.246	0.246	0.246	0.246
-2	-2	0.5	0.5
0.051	-0.976	-0.976	1.079

-0.018	-1.437	-0.727	-0.372
-0.363	-0.363	-0.363	-1.402
1.44	0.682	0.303	-0.076
-0.212	-0.212	-0.212	-0.212
-1.004	-0.395	-1.004	0.517
-1.163	-0.657	0.86	0.86
-1.333	-1.592	-1.333	-1.333
0.928	0.928	-0.049	-0.049
0.252	-0.755	0.252	0.252
0.125	-0.5	-0.188	-1.438
-0.577	0.192	0.192	0.192
-0.862	-0.495	-0.495	-0.495
-2.754	-1.576	-0.692	-0.692
-0.045	0.18	-0.045	0.18
1.401	-2.285	0.295	-1.18
-0.337	0.699	1.217	0.181
-0.692	0.846	0.077	0.846
-0.577	1.429	0.426	1.429
-0.384	-1.024	-1.024	0.256
-0.763	-0.198	-1.894	-0.763
-1.114	-1.114	0.743	0.743
-0.022	-0.022	2.182	-1.344
-0.961	-0.16	-0.56	-1.361
0.333	0.333	1.444	-0.222
-0.082	-1.066	1.887	0.574
-1.088	-0.027	-0.027	1.035
0.052	-0.122	0.052	-0.472
0.53	-0.504	-0.763	-0.763
0.027	0.832	-0.241	0.563
0.178	0.178	1.069	0.178
-2.463	-0.479	-0.149	-0.149
-1.066	1.303	1.303	0.011
0.812	-0.541	1.15	0.474
-0.79	-0.79	0.873	1.704
0.972	0.252	0.252	0.972
0.822	-0.822	-0.164	-0.493
0.936	-1.099	-0.081	-0.081
-1.059	-0.176	0.706	0.265
1.982	-2.287	-0.457	-1.677
-0.046	-0.503	-0.503	0.868
-0.821	-0.189	1.073	-0.821
-3.258	0.465	0.279	-0.093
-0.601	-0.601	-0.954	-0.601
-0.612	-0.612	0.108	-1.333
-0.362	0.195	-0.362	0.751
-0.179	-1.372	-1.372	0.418
-2.002	0.222	-2.002	0.222
-0.202	0.531	-0.202	-0.202
-0.548	-2.194	1.097	0.548
-1.194	-0.276	0.643	-0.735
-0.812	-0.288	-0.288	-0.288
-1.78	-2.383	-1.176	-0.573
-0.399	0.54	-0.399	0.54
0.264	-0.491	-0.491	-0.491
1.723	1.317	0.912	0.101
0.649	0.649	0.649	0.298

-0.06	-0.06	1.543	-0.461
-0.634	-1.285	-0.309	-0.309
0.407	0.407	0.407	0.407
0.187	-2.057	0.935	-1.309
0.587	0	0.587	0.587
-1.31	-1.31	-0.638	-0.638
-0.455	-0.455	-1.213	-0.455
0.184	0.553	0.184	0.184
1.159	0	0	-1.545
0.926	-0.446	0.926	0.926
-0.221	-1.694	0.27	0.27
-0.686	0.33	1.6	0.33
0.482	1.685	0.482	-1.926
0.408	0.068	-0.102	0.579
0.312	-1.249	0.312	1.093
-1.38	-1.725	0.345	-0.345
-1.31	0.288	0.927	-1.31
1.293	-1.058	1.293	-1.842
0.528	-1.988	0.528	0.528
-1.267	1.009	-1.267	-0.508
0.082	-0.737	0.082	-0.737
-0.205	-0.205	-0.205	0.615
0.128	-0.385	0.128	2.184
-0.8	-0.8	0.141	1.083
0.112	0.56	-0.112	-0.56
-0.083	-0.083	0.25	0.917
-0.703	-1.172	1.641	-0.703
-1.508	-0.037	0.699	-0.037
0.466	-0.155	1.088	-0.155
0.851	0.524	0.196	0.524
-0.33	-1.271	0.612	-1.271
-0.192	0.288	0.768	0.288
-0.71	0.567	0.567	0.567
0.412	-1.235	1.235	-0.823
-1.236	-1.81	-0.661	0.489
-0.611	0.367	0.367	1.344
-0.375	-1.876	0.751	-0.375
0.351	-0.428	0.351	0.351
0.872	-1.507	0.476	-0.714
0.262	-1.485	-0.612	1.136
-0.574	-2.214	-0.027	0.246
-1.712	0.677	-2.11	0.677
-0.354	-2.82	-0.354	0.262
0.216	-0.863	0.216	-0.863
0.436	0.436	0.436	1.528
0.524	-2.333	0.524	-0.746
-1.654	-2.179	0.971	-0.079
1.068	-1.941	-0.436	-0.436
-0.177	-0.324	0.124	-0.101

A12 standardization value	A13 standardization value	A14 standardization value	A15 standardization value	A16 standardization value
-1.452	-0.189	-0.189	-0.189	-0.189
-2.318	0.641	0.641	0.641	0.641
-0.685	-0.685	-0.685	-0.685	0.62
0.596	0.596	0.596	0.078	0.078
0.953	0.953	0.953	0	0
-1.578	-0.64	-0.016	-0.64	-0.64
-1.058	0.901	0.901	0.901	0.901
0.81	-0.843	-0.843	-0.512	0.479
-0.474	-2.056	1.333	-0.7	-2.508
-0.233	-1.4	-0.233	-0.233	-0.233
-0.653	1.585	1.585	-0.28	-0.28
1.45	-0.401	0.216	-0.401	0.216
0	0	0.568	1.136	0
-0.42	-0.42	-0.42	-0.42	-0.42
-0.857	-0.857	-0.857	-0.857	-0.857
0.234	-1.465	-1.04	-1.04	-0.616
0.614	0.936	-0.355	-0.355	-0.355
0.502	-0.27	1.274	1.274	-0.656
-0.963	-0.963	-0.963	-0.456	-0.963
0.136	-1.073	0.136	-0.468	1.043
-0.277	-1.387	1.387	-0.832	0.277
-0.192	-1.288	0.356	-0.192	0.905
0.024	0.024	1.463	0.024	0.024
-0.818	-0.614	0.205	0.409	0.818
0.223	0.223	-1.147	0.223	0.565
-0.818	0.043	-0.818	0.905	-0.818
-0.661	-0.086	-0.661	-0.661	0.489
0.134	0.134	-1.653	1.474	1.028
-0.444	0.094	0.363	-0.713	-0.444
-1.465	-0.499	-0.177	-2.109	-1.143
-0.916	0.206	1.328	1.702	-0.916
0.596	-1.612	2.363	0.596	0.596
-1.009	-0.822	1.42	1.046	0.486
-1.648	1.348	0.849	-0.649	-0.15
0.066	-0.43	-0.596	-0.596	-0.596
0.664	0.664	1.614	0.19	0.19
0.957	-0.606	0.176	1.739	0.176
-1.889	-0.068	-0.068	-0.068	-0.068
0.059	1.242	0.848	1.242	0.651
-1.632	0.44	0.44	0.44	0.44
0.537	-1.417	0.537	0.537	0.537
-0.886	-0.454	-0.886	-0.886	-0.886
-0.326	-0.326	2.045	-0.326	-2.104
1.628	-0.345	-0.839	0.641	0.148
-0.622	-0.622	-0.622	-0.622	-0.622
-0.333	-0.333	1.889	0.778	1.889
0.223	-0.462	-0.462	0.223	-0.12
0	0.988	1.975	0	0.988
0.272	0.272	0.272	0.272	0.272
0.031	0.235	1.666	0.031	-1.196
0.59	-0.253	1.432	0.59	-0.253
0.793	0.793	2.087	0.469	1.117
-0.488	0.488	0.488	-0.488	0.488

-0.866	1.513	0.192	-1.13	-1.791
-1.921	-1.479	1.612	0.287	-1.479
-1.78	-0.631	1.665	-0.249	-1.397
0.049	0.049	1.026	0.049	0.049
1.069	1.069	-1.604	-0.267	-0.267
0.329	0.094	0.329	0.329	1.503
-1.755	-0.691	1.436	-0.957	-1.223
-0.919	-0.326	1.452	0.267	-2.104
-0.255	1.02	2.719	-0.255	-0.255
-0.058	-0.058	-0.058	-0.058	-0.058
0.372	-0.066	-0.94	1.684	-0.94
-0.114	-0.686	1.029	0.457	-1.258
-3.111	0.507	0.507	0.507	0.507
-1.424	-0.186	0.433	-0.186	-0.186
2.065	1.126	1.596	-0.751	-1.69
0.641	0.24	1.441	-0.56	-0.56
0.191	1.04	1.04	1.889	-0.234
0.962	0.962	0.962	0.962	0.662
-0.22	0.33	1.703	-1.318	-1.867
-0.352	-0.595	1.105	-1.324	-0.595
-0.628	-0.628	2.162	0.767	-0.163
-1.402	-1.402	0.675	-0.363	0.675
-0.166	-0.166	-0.166	-0.993	-0.166
-0.145	0.819	0.819	0.337	0.819
0.33	0.33	0.33	0.33	1.271
0.089	1.279	0.387	1.279	-0.505
0.171	-0.686	-0.686	-0.686	-0.686
0.258	0.258	0.258	-0.775	0.258
-0.274	-1.058	0.51	-1.058	-1.058
-0.489	0.597	0.597	0.597	0.597
-0.229	-0.229	-0.229	-0.229	-0.229
-1.211	-0.865	-0.173	-0.173	-0.865
-1	0.6	1.8	0.2	0.2
0.584	1.05	1.05	1.05	1.05
-0.384	-1.024	-0.384	0.256	0.256
-0.22	-0.22	-0.22	-0.22	-0.22
0.74	-0.039	1.519	0.74	-0.039
1.073	1.073	1.073	1.073	-1.452
-0.776	-0.776	-0.776	0.948	0.948
-2.38	0.42	0.42	0.42	0.42
-0.065	0.582	1.229	-0.712	-0.065
0.229	0.229	0.229	0.229	0.229
-1.255	0.266	-0.495	-0.495	0.266
0.153	-1.159	-0.722	0.153	0.153
-1.083	0.12	0.12	1.324	0.12
0.033	-0.297	0.033	1.682	-0.297
0.851	0.851	0.851	-0.94	0.851
0.502	-1.428	-1.042	-1.428	-0.27
0.197	-0.461	0.197	0.197	-1.119
-0.207	-0.207	-0.207	-0.207	2.547
1.376	0.229	-0.918	-0.918	-0.918
0.145	0.145	0.145	0.145	0.145
-0.276	-0.702	-0.276	-0.702	0.149
0.415	0.415	-0.415	0.415	0.415
-0.519	0.574	0.027	1.12	0.027
1.008	-1.815	1.008	0.605	-0.605

-1.12	-0.42	0.98	0.28	0.28
0.707	0	0	0.707	0
0.608	-0.743	-2.095	0.608	0.608
-0.378	0.378	1.134	-0.378	-1.89
-0.341	0.634	2.583	0.146	-0.829
0.253	-0.38	2.788	-0.38	-0.38
0.999	-0.081	1.539	1.539	-1.161
-0.777	-0.777	1.295	-0.086	-2.158
-1.117	0.601	0.601	0.601	0.601
-0.539	-0.539	0.323	0.323	-0.539
-0.5	-0.5	0.75	-0.5	0.75
0.108	0.828	1.549	0.108	-0.612
0.084	0.755	1.09	-0.252	-1.593
0.203	-0.109	1.141	-0.735	0.516
0.872	0.388	0.872	0.388	0.388
-0.579	0.064	2.638	0.708	1.351
-1.218	-1.218	-0.316	-0.316	-1.218
0.51	-0.148	0.839	1.167	0.181
-1.024	0.256	2.817	0.256	0.256
0.076	-0.936	0.582	0.582	0.329
0.284	-0.095	1.232	-1.421	0.095
0.666	0.666	0.666	-0.421	0.666
0.298	0.724	1.15	0.298	0.298
0.527	-0.583	-0.028	0.527	-0.583
-0.028	-0.028	1.663	1.663	1.663
0.416	0.416	1.173	-1.853	-1.853
-1.008	-1.008	1.742	-0.092	0.825
-1.514	-1.005	1.794	0.776	1.285
0.173	0.173	0.865	0.865	1.557
1.57	1.57	0.573	1.071	-0.424
-1.081	0.057	1.764	0.057	0.057
0.189	1.452	0.189	0.189	1.452
0.044	0.044	0.483	0.483	0.483
-0.101	-0.507	1.115	1.115	-2.128
-1.419	-0.63	1.734	-0.63	-0.63
-0.112	0.448	1.569	0.168	0.448
0	-0.877	-0.877	-0.877	-0.877
0.774	0.167	0.774	0.167	-1.351
-1.138	-0.103	-0.448	-0.103	0.241
1.473	0.937	-0.134	0.402	-0.669
1.425	2.087	1.093	-0.232	-0.232
0.392	0.392	0.392	0.392	0.392
0.905	-1.106	-0.101	0.905	-2.111
0.25	0.668	1.085	-0.167	-0.584
-0.571	0.857	0.143	-0.571	-0.571
0.793	0.793	0.159	-1.11	-0.476
-0.229	-0.229	-0.229	-0.229	-0.229
0.374	1.205	1.205	-0.457	-0.457
0.192	-0.505	-0.505	-0.505	-0.505
0.833	0.055	-0.413	0.21	0.366
0.626	0.626	0.626	0.125	-1.878
0.44	0.44	0.44	0.44	0.44
-0.703	-0.703	-0.703	-0.703	-0.703
-0.456	0.246	0.246	-0.456	0.246
-2	0.5	0.5	-2	0.5
-2.003	0.051	-0.976	1.079	1.079

-1.082	-0.372	0.337	2.465	-1.082
-0.363	-1.402	-0.363	-0.363	-0.363
0.682	-1.592	1.061	-0.076	-0.834
-0.212	-0.212	-0.212	-0.212	-0.212
-0.7	0.517	1.125	-0.7	-1.004
0.86	0.86	0.86	-0.152	0.86
-1.333	0.996	0.996	0.996	0.996
-0.049	0.928	-1.026	-0.049	0.928
0.252	0.252	0.252	1.258	0.252
-0.5	-0.5	-0.5	-0.5	-0.5
-0.577	0.192	-0.577	-0.577	-0.577
-0.495	0.239	2.073	0.605	-0.128
0.486	0.486	0.486	0.486	0.486
0.63	-3.644	-0.495	-0.27	-0.27
1.032	-0.442	-0.442	-0.074	-1.18
0.699	-0.337	0.699	-0.855	-1.373
0.846	0.846	0.462	-0.308	0.462
0.175	-0.326	-0.827	-0.326	-0.577
-1.024	-1.024	-1.024	0.256	-1.024
-0.198	2.064	0.933	-0.763	-0.763
0.743	0.743	0.743	0.743	0.743
-0.463	-0.904	0.419	-0.463	-0.904
-0.56	-0.961	0.641	-1.361	0.24
-0.778	0.333	0.889	-0.778	-0.778
-1.066	-0.738	-0.738	-0.738	-0.738
-0.557	1.566	-0.557	2.097	-1.619
-0.822	-0.647	-0.997	2.151	2.151
-0.763	-0.763	-0.763	0.272	-0.763
0.832	0.027	1.368	0.295	0.832
0.178	0.178	0.178	0.178	0.178
-0.479	-0.149	-1.471	-0.149	-0.149
0.226	1.088	1.303	0.442	-0.205
-0.203	0.812	2.503	-0.879	-0.879
0.873	0.042	0.042	0.042	0.873
-0.468	0.252	-1.188	-1.188	-1.188
0.164	0.164	0.493	0.493	-1.48
0.733	-0.285	-0.896	-1.303	-2.117
-1.059	-0.618	2.029	-1.059	-0.176
-0.457	-0.457	-0.457	0.762	1.372
1.782	0.868	0.411	-0.96	-0.046
-1.452	-0.189	2.335	0.442	1.073
0.279	-0.465	1.21	1.21	1.024
1.519	1.519	1.519	1.519	1.519
-0.612	0.108	-0.612	0.108	0.108
0.751	0.195	1.865	0.751	-0.918
1.014	1.014	1.014	1.014	-0.179
0.222	0.222	0.222	0.222	0.222
-0.202	1.264	1.264	-0.568	1.631
-0.274	-0.274	0.548	0.548	-2.742
-0.276	-0.735	0.184	-1.194	-0.735
-0.812	-0.812	0.759	1.283	1.283
-1.176	0.03	0.633	0.633	0.633
-0.399	-0.399	0.54	-0.399	-3.689
-0.491	-0.491	-0.491	-0.491	-0.491
-0.304	1.723	-0.304	-0.709	-0.709
1	-0.053	-0.053	-0.053	-0.053

-0.862	-1.262	-1.262	-1.262	-1.262
-1.936	0.342	1.318	0.992	1.318
0.407	0.407	0.407	0.407	0.407
-0.561	0.935	-0.561	-0.561	-1.309
0.587	-1.174	0.587	-0.587	-3.523
0.034	-0.638	2.946	-0.414	-0.414
-1.971	-1.213	1.819	-0.455	0.303
-1.66	0.184	2.766	-0.922	-0.184
-1.159	-0.386	1.159	-0.386	-1.159
0.926	-0.446	0.926	-0.446	-0.446
0.27	0.27	0.27	0.27	0.27
-0.432	0.076	1.6	0.838	-1.193
-0.722	-0.722	0.482	-0.12	-0.12
0.919	0.579	1.089	0.408	0.749
-0.469	1.874	-0.469	0.312	1.874
0	0	0	-0.345	-0.345
-1.31	-0.032	1.566	-0.032	-0.032
0.51	-1.058	0.51	-0.274	0.51
0.528	0.528	0.528	0.528	0.528
1.009	-0.508	-0.205	-0.205	-0.053
-0.737	0.082	0.082	0.082	0.082
-0.205	0.615	0.615	1.435	-1.845
-0.385	-0.385	-0.385	-0.385	-0.385
1.083	-1.742	0.141	1.083	-0.8
-0.56	-0.56	1.679	-0.112	-0.336
0.584	-0.083	1.251	-0.083	-1.418
0.703	0.703	1.641	-1.172	-2.578
0.699	0.699	0.699	0.699	0.699
-0.777	-2.02	-0.155	-0.155	-0.777
0.524	0.851	0.524	0.851	-1.44
0.612	0.612	0.612	1.554	-1.271
0.768	1.248	1.248	-1.152	-2.112
-1.668	0.567	0.567	0.567	-1.349
-0.412	-0.412	2.058	0	-1.235
-0.661	1.638	-0.086	-0.086	1.063
1.344	-0.122	1.344	-0.611	-1.1
-1.126	-0.751	1.501	0.375	-1.876
-0.428	-1.987	0.351	-0.428	0.351
-1.507	1.665	0.872	-0.317	-0.714
-0.612	1.136	1.136	0.262	-0.612
0.519	0.246	0.793	-0.027	-2.214
0.677	0.677	-1.314	-0.119	-0.518
1.187	-0.663	1.495	0.262	-0.971
-1.941	-0.863	1.294	1.294	-0.863
0.436	-0.655	0.436	0.436	-0.655
1.158	0.524	0.524	0.524	-0.111
-1.129	0.446	0.446	-0.604	-0.079
1.068	-0.135	1.068	-0.135	-0.135
-0.144	-0.019	0.529	0.056	-0.226

A17 standardization value	A18 standardization value	A19 standardization value	A20 standardization value
-0.189	-0.189	-0.189	-0.189
0.641	-2.318	0.641	0.641
-0.033	0.62	0.62	0.62
0.078	0.078	0.596	0.596
-0.953	-0.953	0.953	0
-0.328	-0.64	-0.328	-0.64
0.901	-2.626	0.901	-1.058
1.141	1.141	1.471	1.141
-0.023	1.107	-0.7	-0.249
-0.233	0.933	0.933	2.683
-0.28	-0.28	0.093	1.212
1.45	0.833	0.216	0.833
0	0	0	0
-0.42	-0.42	-0.42	-0.42
-0.857	-0.857	1.048	1.048
0.234	0.234	0.234	-2.738
-0.355	-0.355	-0.355	-0.355
-1.042	-1.042	-1.042	1.274
-0.963	-0.963	-0.456	0.051
0.438	0.74	1.647	1.345
0.277	-0.277	0.832	-0.832
0.905	0.356	0.356	0.356
0.024	0.024	0.024	0.024
0.818	1.023	1.841	1.841
0.223	0.223	0.223	0.223
0.043	0.043	-0.818	0.043
0.489	1.063	1.638	1.063
-0.76	1.028	-1.206	0.134
-0.444	1.708	1.708	0.363
0.467	-1.143	1.111	1.111
-0.542	-0.542	-1.29	-0.168
0.155	-0.729	-1.17	-0.287
-0.262	-0.262	0.673	-0.635
-0.649	0.35	-1.149	-0.649
-0.596	0.066	0.562	-0.099
0.19	0.19	-0.759	1.139
-0.215	0.176	0.567	0.176
-0.068	-0.068	-0.068	-0.068
-0.138	0.651	0.848	0.848
0.44	0.44	0.44	0.44
0.537	0.537	0.537	0.537
1.276	-0.022	0.411	0.411
0.267	-0.326	1.452	1.452
-0.345	0.641	-0.839	0.641
-0.03	-0.622	-0.03	-0.622
0.778	-1.444	1.889	0.778
-0.462	-0.12	-0.462	0.223
0.988	-1.482	-0.988	-0.494
0.272	0.272	0.272	0.272
0.031	-0.992	-0.174	-0.378
-0.253	-0.253	-1.095	-1.095
0.469	0.469	0.469	-0.825
-0.488	-0.488	2.44	-0.488

-0.469	0.059	0.852	1.513
-1.038	0.729	0.729	1.17
-0.631	1.665	0.517	1.282
-0.928	-0.928	0.049	0.049
1.069	1.069	1.069	1.069
0.798	-0.141	-0.845	-0.61
0.904	0.904	1.436	-0.691
-0.919	-0.919	0.859	-1.511
0.595	-0.68	-0.255	1.869
-0.058	-0.058	-0.058	-0.058
0.372	0.809	-0.503	-0.066
-0.114	-1.258	-0.686	-0.686
0.507	0.507	0.507	0.507
-0.186	0.433	-0.186	-0.186
1.126	-0.282	-0.282	0.188
0.24	1.441	0.641	-0.16
0.191	-0.234	-0.234	-0.234
0.662	0.962	0.662	0.962
0.33	-2.142	0.33	1.703
-0.352	0.619	1.105	1.834
-0.628	2.162	0.302	0.767
0.675	-0.363	0.675	-0.363
-0.166	-0.166	-0.166	-0.166
-0.145	0.337	0.819	0.337
0.33	0.33	1.271	0.33
-0.803	-1.1	1.279	-0.208
-0.686	1.886	-0.686	0.171
0.258	0.258	0.258	0.258
0.51	-0.274	1.293	2.077
0.597	0.597	-1.575	-1.575
-0.229	-0.229	-0.229	-0.229
-0.173	-0.173	-0.519	-0.173
0.2	1	1	1
1.05	1.05	-0.817	0.584
-1.024	0.896	0.256	-0.384
-0.22	-0.22	-0.22	-0.22
-0.818	-1.597	-0.818	-1.597
-1.452	-1.452	-1.452	-1.452
0.948	-0.201	0.374	-0.201
0.42	-2.38	0.42	0.42
-1.358	0.582	-0.712	0.582
0.229	0.229	0.229	0.229
1.027	-0.495	-1.255	0.266
-0.722	2.34	2.34	1.028
-1.083	-2.287	0.12	0.12
-0.297	-2.276	-0.627	1.352
-0.045	1.746	-0.045	1.746
-0.656	0.116	-0.656	0.502
-1.119	0.197	0.855	0.197
-0.207	-0.207	1.17	-0.207
-0.918	0.229	1.376	0.229
0.145	0.145	0.145	0.145
-0.276	2.701	-0.276	-0.276
0.415	0.415	0.415	0.415
-2.705	-0.519	0.027	1.667
-0.605	-0.605	-0.605	-0.605

0.28	1.68	0.98	0.98
0	-0.707	-0.707	0
-0.743	-2.095	0.608	0.608
-0.378	1.89	0.378	-0.378
1.121	-1.803	0.146	-0.341
-0.38	0.253	-0.063	-0.063
1.539	-1.161	0.459	0.459
0.259	0.604	0.259	0.95
-0.544	0.029	0.601	-0.544
0.754	-1.4	-0.539	-0.539
0.75	-0.5	0.75	0.75
-1.333	-1.333	-0.612	-1.333
-0.922	-0.922	0.755	1.426
0.516	-1.986	1.141	0.829
0.388	0.388	0.388	0.388
-0.901	0.708	0.064	0.708
-1.218	1.489	-0.767	1.489
-0.806	0.181	0.51	-0.148
0.256	0.256	-0.384	0.256
0.582	-0.177	0.329	0.329
-1.611	-1.611	-1.232	-1.421
0.666	0.666	0.666	0.666
0.298	1.15	1.15	-0.554
-0.028	-0.028	-0.028	-0.028
-0.028	-1.156	-1.156	0.535
0.416	0.416	-0.34	1.173
-1.008	-0.092	1.742	-1.008
0.013	1.03	-0.751	1.03
0.173	0.173	-1.903	-1.211
0.573	0.573	0.075	-0.424
0.057	-0.512	0.057	0.626
1.452	0.189	0.189	0.189
1.361	-1.273	0.483	0.483
0.304	-0.101	-0.101	1.115
-0.236	0.158	0.158	-1.419
-1.513	1.289	-0.672	1.569
0	-0.877	0	0.877
0.167	0.471	0.471	0.167
-0.103	-0.103	0.241	0.586
-0.134	-0.134	-1.205	1.473
-0.232	0.431	0.431	0.431
0.392	0.392	0.392	0.392
0.905	0.905	-1.106	0.905
-0.584	-2.671	0.668	0.668
0.857	0.857	0.143	0.857
0.159	-1.11	1.427	1.427
-0.229	-0.229	-0.229	-0.229
-1.288	0.374	2.868	0.374
-0.505	-0.505	-0.505	-0.505
0.522	0.677	0.522	0.522
-1.878	0.626	0.626	0.626
0.44	0.44	0.44	-0.537
-0.703	-0.703	-0.703	-0.234
0.246	0.948	-2.563	-2.563
0.5	0.5	0.5	0.5
0.051	1.079	-0.976	-0.976

0.692	0.337	0.337	1.756
-0.363	-0.363	-0.363	-0.363
-0.076	-1.971	-0.834	1.061
-0.212	-0.212	-0.212	-0.212
-1.004	1.43	-1.004	-1.004
0.354	0.86	0.86	0.354
0.996	0.996	0.996	0.996
0.928	-1.026	-1.026	0.928
1.258	0.252	0.252	0.252
-0.5	-0.5	-0.5	-0.5
-0.577	0.192	-0.577	-0.577
0.239	-0.128	1.339	0.239
0.191	1.075	1.075	1.075
-0.27	0.18	1.53	-0.72
-0.442	-0.811	-0.442	0.295
-0.337	-0.337	2.253	1.735
0.462	0.462	0.077	-0.308
-0.326	0.677	-0.075	0.175
0.896	0.256	2.177	0.256
-0.763	-0.763	-0.763	-0.763
0.743	0.743	0.743	0.743
-0.904	-0.463	-0.463	-0.022
-1.361	0.641	0.24	0.24
0.889	-0.778	-0.778	-0.778
-0.738	-0.738	-0.738	-0.738
-1.088	-0.027	-0.557	0.504
2.151	0.052	-0.122	-0.122
-0.763	1.823	1.823	0.53
0.563	0.027	0.295	1.905
0.178	0.178	0.178	0.178
-0.149	-0.479	0.512	0.512
0.011	-0.851	-1.928	-0.636
-0.879	-0.879	-0.879	-0.879
-0.79	0.042	0.042	0.042
-1.909	0.252	0.252	-1.188
-0.822	-1.808	0.164	0.164
-0.285	-0.692	-0.285	0.326
0.706	0.706	0.265	-0.176
-0.457	0.762	-0.457	-0.457
-0.046	-0.046	-0.046	-0.046
0.442	0.442	1.704	0.442
0.279	0.279	0.279	0.652
-0.247	0.106	0.106	0.813
1.549	-1.333	-1.333	1.549
0.751	0.195	0.195	0.751
-0.179	-1.969	1.014	1.611
0.222	0.222	0.222	0.222
1.264	-0.568	-1.667	0.165
-0.274	0.274	0.823	0.548
0.184	0.184	-1.194	-0.276
0.759	-0.288	-0.812	-0.288
0.633	0.633	0.03	0.633
0.54	-1.339	0.54	0.54
2.531	2.531	1.397	1.02
-1.52	-1.115	-0.304	-0.304
-0.403	0.298	1.35	0.298

-0.06	-0.862	-0.06	-0.06
-0.96	0.667	-0.309	-0.309
0.407	0.407	0.407	0.407
-0.561	0.935	0.187	0.935
0.587	0.587	0	0.587
0.034	-0.414	0.258	0.034
0.303	0.303	-0.455	0.303
-0.922	-0.922	-0.553	-0.553
-0.386	0	1.159	-0.386
-0.446	-1.817	-1.817	0.926
0.27	0.27	0.27	0.27
-0.178	-1.193	0.584	1.346
0.482	-0.722	-0.12	0.482
0.068	0.749	0.749	0.919
0.312	-0.469	0.312	-1.249
-0.69	0	0	-0.345
0.607	-0.032	1.246	1.246
0.51	-1.058	1.293	1.293
0.528	0.528	0.528	0.528
0.25	1.009	1.009	1.009
0.082	0.082	0.082	0.082
0.615	-1.025	0.615	1.435
-0.385	-0.385	-0.385	-0.385
0.141	1.083	0.141	-0.8
-0.112	-1.455	-0.336	0.784
0.25	-2.418	-1.084	-0.751
0.234	-0.234	0.234	-0.234
-0.037	-0.037	-0.037	-0.037
-0.777	1.088	0.466	1.088
-2.094	-1.44	0.524	-0.458
0.612	0.612	-0.33	-0.33
0.768	-1.152	-1.152	0.288
0.567	-0.231	0.567	-0.231
-0.412	-1.235	-0.412	0.823
-0.661	1.063	2.213	-0.086
-0.122	-1.1	1.344	1.344
-0.375	0.375	-0.375	-0.375
0.351	1.13	1.13	1.13
0.872	0.079	-0.317	-0.714
0.262	-0.612	0.262	1.136
-0.027	1.066	-0.574	-0.027
0.677	0.279	0.677	0.677
0.262	1.187	0.57	0.878
1.294	-1.941	1.294	0.216
-0.655	-0.655	0.436	-0.655
0.841	-1.698	0.524	0.524
0.971	-0.604	-1.129	-1.129
1.068	1.068	0.767	1.068
0.004	-0.049	0.164	0.237
